# Metazoan Remaining Genes for Essential Amino Acid Biosynthesis: Sequence Conservation and Evolutionary Analyses

**DOI:** 10.3390/nu7010001

**Published:** 2014-12-24

**Authors:** Igor R. Costa, Julie D. Thompson, José Miguel Ortega, Francisco Prosdocimi

**Affiliations:** 1 Instituto de Bioquímica Médica Leopoldo de Meis, Universidade Federal do Rio de Janeiro, Rio de Janeiro 21941-902, RJ, Brazil; E-Mail: igorc@ufrj.br; 2 Department of Computer Science Research, ICube Laboratoire des sciences de l’ingénieur, de l’informatique et de l’imagerie, CNRS/Université de Strasbourg, 11 rue Humann, Strasbourg F-67000, France; E-Mail: thompson@unistra.fr; 3 Departamento de Bioquímica e Imunologia, Instituto de Ciências Biológicas, Universidade Federal de Minas Gerais, Belo Horizonte 31270-901, MG, Brazil; E-Mail: miguel@icb.ufmg.br

**Keywords:** comparative genomics, essential amino acids, molecular evolution

## Abstract

Essential amino acids (EAA) consist of a group of nine amino acids that animals are unable to synthesize via *de novo* pathways. Recently, it has been found that most metazoans lack the same set of enzymes responsible for the *de novo* EAA biosynthesis. Here we investigate the sequence conservation and evolution of all the metazoan remaining genes for EAA pathways. Initially, the set of all 49 enzymes responsible for the EAA *de novo* biosynthesis in yeast was retrieved. These enzymes were used as BLAST queries to search for similar sequences in a database containing 10 complete metazoan genomes. Eight enzymes typically attributed to EAA pathways were found to be ubiquitous in metazoan genomes, suggesting a conserved functional role. In this study, we address the question of how these genes evolved after losing their pathway partners. To do this, we compared metazoan genes with their fungal and plant orthologs. Using phylogenetic analysis with maximum likelihood, we found that acetolactate synthase (ALS) and betaine-homocysteine *S*-methyltransferase (BHMT) diverged from the expected Tree of Life (ToL) relationships. High sequence conservation in the paraphyletic group Plant-Fungi was identified for these two genes using a newly developed Python algorithm. Selective pressure analysis of ALS and BHMT protein sequences showed higher non-synonymous mutation ratios in comparisons between metazoans/fungi and metazoans/plants, supporting the hypothesis that these two genes have undergone non-ToL evolution in animals.

## 1. Introduction

It has been known for decades that most animal cells are incapable of growing in a medium lacking amino acid supplements [1]. This means that pathways for *de novo* amino acid biosynthesis are missing in their genomes, characterizing the Essential Amino Acid (EAA) phenotype. There is no consensus over the exact number of essential amino acids, but it is normally accepted that His, Ile, Leu, Lys, Met, Phe, Thr, Trp and Val belong to this group. Although this phenotype was discovered in 1932, it was only recently that a two research groups independently studied a large number of animal genomes in an attempt to identify precisely which genes encoding enzymes for the EAA pathways are absent in animal genomes [2,3].

The EAA phenotype was found to be apomorphic in the metazoan clade, indicating that a number of genes were lost in the genome of an ancestral heterotroph organism. Moreover, it has been reported that the loss of an entire pathway for amino acid biosynthesis could also be observed in a number of other groups of organisms, mainly in bacteria and protists [2] capable of obtaining that specific amino acid from their surroundings.

The metazoan ancestor must have had a considerable supply of amino acids in order for it to survive after the complete loss of many EAA biosynthesizing enzymes. Three non-exclusive explanations could justify the increased availability of dietary amino acids: (i) the development of heterotrophy, after the acquisition of a digestive cavity or unicellular predation; (ii) the association with symbiotic organisms that provided the amino acid supply [2]; or (iii) the acquisition of efficient transmembrane transporters. In the metazoan lineage, no organism has been found that produces an intermediate number of essential amino acids, with possible exceptions in *Cnidaria* [3,4], suggesting a unique deletion event.

Hypothetically, when organisms lose key enzymes in a pathway, genes encoding their upstream or downstream pathway partners should experience relaxation in the selection pressure. Such relaxation has been observed in whole genome duplication events, since many gene copies turn out to be unnecessary [5,6,7]. In other extreme cases, such as in the metazoan EAA pathways, we suggest that this relaxation may lead to a pseudogenization cascade that might result in the deletion of each and every gene participating in a given pathway. However, when performing the evolutionary analysis of EAA biosynthetic pathways [2,8,9] in animals, our group found that some genes from these pathways are still present in metazoan genomes. In this manuscript, we analyze the sequence conservation and evolutionary fate of those metazoan REmaining GENes (ReGens).

Two different hypotheses could explain the presence of these genes in metazoan genomes: (i) the first and most obvious reason would be that they continue to perform the same functions they once did, or a subset of those. Most of the biochemical reactions originally performed by enzymes in the EAA biosynthesis pathway might be performed by symbiotic bacteria. This might provide the animal cells with the intermediate metabolites for the missing steps and the enzymes could use these as substrates to complement their biosynthesis pathways. It is also known that some proteins are able to perform different reactions at the same catalytic site, using similar substrates and generating distinct, but related products. The enzymes for the EAA synthesis might participate in such anaplerotic pathways and perform the same biochemical reaction. Moreover, it is well known that enzymes involved in biosynthetic pathways are often capable of working in the reverse reactions and at least some of the remaining enzymes might be used in the degradation steps for their respective amino acid. Thus, the selective pressure relaxation caused by the loss of pathway partners might not be enough to modify these proteins considerably or cause their complete pseudogenization.

An alternative hypothesis is that these remaining genes may have evolved in some particular way only in the metazoan clade, as a consequence of losing their partners. We can imagine a scenario in which entire regions or protein domains related to the lost function would accumulate neutral substitutions and/or undergo positive selection. It is also possible that the remaining genes could accumulate mutations and acquire a new function. In the evolution after gene duplication framework such events would be called subfunctionalization and neofunctionalization, respectively.

As we know from gene duplication studies, genes may change their function with only a small number of mutations, characterizing neofunctionalization. This makes neofunctionalization events especially difficult to infer based solely on sequence analyses of distant clades. Nevertheless, we believe that an EAA remaining gene showing strong signals of non-ToL evolution in metazoans is unlikely to have exactly the same functions as their autotrophic homologs.

**Figure 1:**
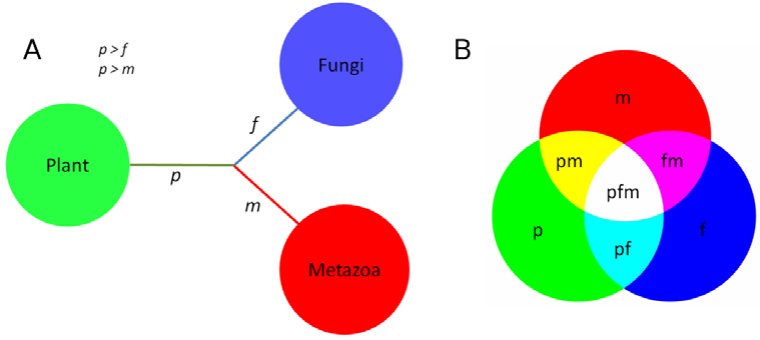
Clade comparison and color code definitions. (**A**) Tree of Life topology of the clades compared here: a more recent common ancestor is known between fungi and metazoans (*Opisthokonta* clade); (**B**) color code for clade comparison.

Using a three clade comparison model (plants, fungi and metazoans), we investigated the effect of the loss of pathway partners on the conservation of the ReGens. We took advantage of the fact that (i) plants and fungi are known to be autotrophic organisms capable of producing all amino acids by *de novo* pathways and (ii) fungi and metazoans share a most recent common ancestor and group together in the *Opisthokonta* clade according to the Tree of Life (ToL) (Figure 1). Therefore, we used a set of molecular evolutionary metrics, including nucleotide and amino acid conservation, phylogeny using maximum likelihood, and non-synonymous mutation (Ka) ratio, as well as newly developed bioinformatics strategies to determine the fate of metazoan ReGens. Genes will be classified under ToL or non-ToL evolutionary models whether the observed results respect or not the *Opisthokonta* clade topology.

Here we study the sequence conservation of the whole set of ReGens, consisting of eight enzymes originally involved in the EAA biosynthetic pathways and shown to be present in the human and most animal genomes [2].

## 2. Experimental Section

### 2.1. Database Searches for Genes Involved in Essential Amino Acid Biosynthesis

Based on previous articles [2], we performed sequence searches to find all the enzymes responsible for the biosynthesis of essential amino acids in yeast (*Saccharomyces cerevisiae*). Each gene in this set was used as a query for a BLAST-based similarity search [10] in metazoan genomes (i) to retrieve putative homologs and (ii) to search for deleted genes. Genes were considered homologs based on annotation and on being able to retrieve the original query using BLAST in the yeast genome.

### 2.2. Curation of Metazoan Homologs of Yeast EAA Biosynthetic Enzymes

All of the eight metazoan homologous genes involved in EAA biosynthesis were manually investigated in databases such as UNIPROT [11], BRENDA [12] and KEGG [13], for their known molecular function.

### 2.3. Phylogenetic Analysis of ReGens

BLAST searches in the NCBI Refseq database were conducted, in order to find putative homologs from other species of plants, fungi and metazoans for each of the eight curated genes. These protein sequences were multiply aligned using ClustalW [14], Muscle [15] and MAFFT [16] using default parameters. Phylogenetic analyses were then performed using the PhyML software [17]. The evolutionary history was inferred using the Maximum Likelihood method based on the JTT matrix [18] of amino acid substitution. Five-hundred bootstrap replicates were used to evaluate the phylogenetic consistency.

### 2.4. Conservation Pattern

A Python script was developed to visualize the amino acid conservation profiles of ReGens and their putative homologs in fungi and plants. Amino acid sequences from all clades were multiply aligned using ClustalW and each residue was compared, using the BLOSUM 62 matrix, with all residues located in the same alignment column (representing the same molecular character) (Supplementary material 1). Amino acid conservation scores were divided into groups for pairwise clade comparisons: Metazoan X Fungi, Fungi X Plants and Plants X Metazoan. The character-to-character BLOSUM value is summed for each alignment column and finally averaged for each pairwise clade comparison. This average value is further filtered to remove the high frequency noise using a low pass filter (See formula below).

*y_n_* = *αx_n_* + (1 − *α*)*y*_*n*−1_ (1)

This filter is used to observe local tendencies of conservation, instead of the score of a single position. It filters the average comparison score for every position in the multiple alignment (*x_n_*), multiplying this value by a constant alpha, 0 < α < 1 (we used α = 0.05, higher means less noise and less resolution) to produce the final value shown on the *y*-axis at the *n*^th^ position (*y_n_*). As this filter is directional, we plot the average results for both the original alignment and the reverse alignment. This program uses the graphical library MatPlotLib [19] to plot the results.

### 2.5. Back Translation

The ClustalW multiple alignments of proteins from all clades were back-translated to nucleotide sequences using a Python script that searches and replaces amino acids by their original codons.

### 2.6. Ka/Ks Estimation Using PAML

Pairwise and branch specific Ka/Ks estimation was done using the codeml program of the PAML package, using the PhyML maximum likelihood tree and the back translated multiple alignment as inputs. Pairwise comparisons were separated into three categories, Fungi X Metazoans, Fungi X Plants and Metazoans X Plants. Finally, the Ka/Ks values were averaged for each clade comparison.

## 3. Results

### 3.1. Finding and Describing Human ReGens

A complete dataset containing all the yeast (*Saccharomyces cerevisiae*) protein sequences for EAA biosynthesis, consisting of 49 proteins, was produced through manual curation in the UniProt database [11] (see Supplementary material 2). All these sequences were used as BLAST queries to search the RefSeq [20] database for putative homologs in metazoan genomes. Eight of the 49 enzymes were found to have clearly identifiable homologs in metazoans, as estimated by sequence identity and protein coverage (Table 1). They were carefully annotated and their molecular function was manually investigated in both the literature and databases such as UNIPROT [21], KEGG [13], GO [22], Brenda [12] and CDD [23].

We confirmed that five of the eight ReGens are currently annotated as members of other non-EAA metabolic pathways. For instance, besides participating in the biosynthetic pathway for the EAA methionine, cystathionine gamma-lyase also participates in the biosynthesis of the non-EAA cysteine. We also verified that at least one ReGen, the aromatic/aminoadipate aminotransferase 1 was not found in plants, since organisms in this clade use a different pathway to synthesize lysine (Table 1). This enzyme was therefore excluded from subsequent analyses.

Thus, saccharopine dehydrogenase (SD), betaine-homocysteine *S*-methyltransferase (BHMT) and acetolactate synthase (ALS) were the three remaining genes with no other known function assigned besides EAA biosynthesis.

**Table 1:** Essential amino acids (EAA) biosynthesis enzymes with homologs in metazoans.

Enzyme Name	Acronym	Yeast ID	Human ID	EC Number	EAA	Pathway Functions
Acetolactate synthase	ALS	P07342.1	NP_006835.2	2.2.1.6	Val, Leu, Ile	Second reaction of the branched-chain amino acid biosynthesis pathway
Betaine-homocysteine *S*-methyltransferase	BHMT	Q12525.1	NP_001704.2	2.1.1.5	Met	Last reaction of the Met biosynthesis pathway
				2.1.1.10		
Branched-chain-amino acid aminotransferase cytosolic	BCA	P47176.1	NP_005495.2	2.6.1.42	Val, Leu, Ile	Last reaction of the branched-chain amino acid biosynthesis pathway
Saccharopine dehydrogenase	SD	P38999.1	NP_005754.2	1.5.1.7	Lys	Last reaction of the Lys biosynthesis pathway
Cystathionine gamma-lyase	CTH	P31373.2	NP_001893.2	4.4.1.1	Met	Biosynthesis of Cys and Met
Aspartate aminotransferase, mitochondrial	AATm	Q01802.2	NP_002071.2	2.6.1.1	Phe	Ala, Asp, Glu, Cys, Met, Arg, Pro, Tyr, and Phe metabolism
Aspartate aminotransferase, cytoplasmic	AATc	P23542.3	NP_002070.1	2.6.1.1	Phe	Ala, Asp, Glu, Cys, Met, Arg, Pro, Tyr, and Phe metabolism
Aromatic/aminoadipate aminotransferase 1	AadAT *	P53090	NP_057312.1	2.6.1.39	Lys	Fifth reaction of the Lys biosynthesis pathway

* AadAT is absent in plants, since they use an alternative pathway to produce Lys. This last enzyme was not analyzed here.

### 3.2. Known Functions of the SD, BHMT and ALS Proteins

The human homolog of saccharopine dehydrogenase (SD) was first described in 2000 [24] and confirmed to function in the degradation pathway of lysine in metazoans and plants [25,26]. SD in these clades is bi-functional and catalyzes the first and second reactions of the lysine degradation pathway. In fungi, this enzyme is encoded as two independent genes, each of them being responsible for one reaction.

Betaine-homocysteine *S*-methyltransferase (BHMT) catalyzes the reversible methylation of homocysteine to methionine [27] and has been the subject of gained renewed interest after the discovery that the blood concentration of homocysteine is related to human health problems such as: thrombosis, vascular [28,29,30,31,32] and congenital diseases, such as spina bifida [33,34], as well as Alzheimer disease [35,36]. The inhibition or deletion of BHMT causes hyperhomocysteinemia in mice [37,38]. Functional assays of BHMT in metazoans have shown that it uses betaine and l-homocysteine as substrates, generating l-methionine and dimethylglycine [39]. However, in fungi and plants, it uses *S*-methyl-l-methionine and l-homocystheine as substrates producing 2 l-methionine; or uses *S*-adenosyl-l-methionine and l-homocysteine generating *S*-adenosyl-l-homocysteine and l-methionine [40,41]. Moreover, a paralogous gene of BHMT has been found in therians, called BHMT2 (see Supplementary material 3). This gene performs the same molecular reaction as the plant and fungi orthologs, suggesting that BHMT may have duplicated in a therian ancestor allowing one of the copies to neofunctionalize and stop using betaine as substrate [42].

Acetolactate synthase (ALS) has been extensively studied in plants, since it is the target of four classes of high spectrum herbicides: (i) sulfonylureas [43]; (ii) imidazolines [44]; (iii) triazolopyrimidines and (iv) pyrimidyl-oxy-benzoates [45]. These herbicides are considered relatively safe for humans, since it is assumed we lack the ALS enzyme due to our inability to synthetize branched-chain amino acids [46]. Joutel *et al.* [47] described a human ALS homolog for the first time, while looking for the genetic cause of a mental disorder called CADASIL (Cerebral Autosomal Dominant Arteriopathy with Subcortical Infarcts and Leucoencephalopathy). They subsequently found that this gene is conserved in every metazoan they tested and it is ubiquitously expressed in human tissues [47]. We were unable to find a definitive source of information about human ALS functions. One group published an attempt to express the human homolog of the ALS in *E. coli* [48], but they failed to measure significant acetolactase synthase activity of the recombinant enzyme and proposed that the human gene does not encode ALS. However, due to incorrect folding and lack of posttranslational modifications and other limitations of the bacterial expression model, this experiment did not determine whether or not the human ALS has the original acetolactate synthase function. To our knowledge, there have been no other studies published using knockout animal models, or any other attempts to characterize metazoan ALS homologs.

### 3.3. Conservation of Protein Sequences between Clades

The sequences encoding the homologs of ReGens in a number of selected organisms were retrieved from the RefSeq database (Table 2). Manually curated orthologous groups were created for each ReGen. In the case where a given ReGen presented multiple family members (paralogs) in a given genome, we chose the version that was the most similar to the fungi sequence used as query.

All multiple alignment tools provided efficient and congruent sequence alignments (data not shown) and we proceeded with ClustalW data (see Supplementary material 4).

**Figure 2:**
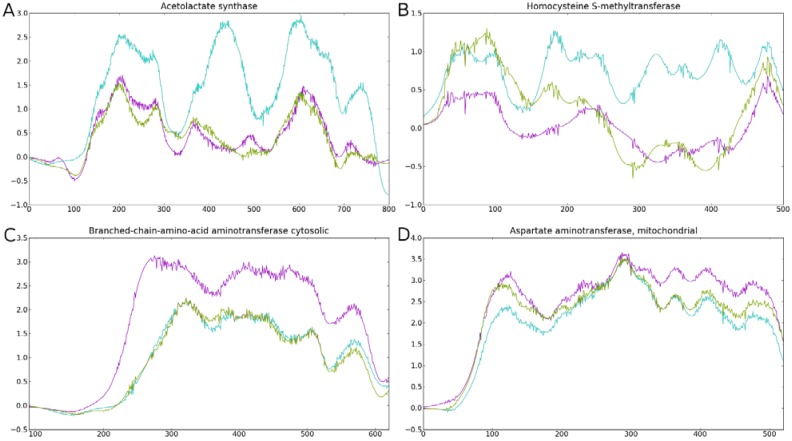
Amino acid conservation between clades for four ReGens. Lines are colored according to the clade comparison code defined in Figure 1B. Pairwise comparisons of proteins from each clade were plotted along their multiple alignment extension (*x*-axis). Higher values (*y*-axis) represent more similar regions in the interclade comparison. The *y*-axis represents the proportion of amino acid substitutions in a given position of the multiple alignments (*x*-axis); positions with gaps were removed. (**A**) ALS; (**B**) BHMT; (**C**) BCA; (**D**) AATm.

Using the Python programming language, together with Biopython and MatProtLib libraries, we developed a visualization script to provide an easy and intuitive view of sequence conservation between tens of proteins derived from distant clades. Figure 2 and Supplementary material 5 show the amino acid conservation profiles of metazoan ReGens and their putative orthologs in fungi and plants and Supplementary material 6 presents the graphs containing standard deviation information. The profiles showed higher conservation in the *Opisthokonta* clade for the BCA, AATm and CTH (Figure 2C,D and Supplementary material 5C, magenta line), in line with the ToL-like model, although it did not identify any clear significant differences between clades for the SD and AATc (Supplementary material 5A,B).

Surprisingly, we found that some regions in ALS and BHMT protein sequences were more conserved between fungi and plants (Figure 2A,B; cyan line) than between metazoans and fungi (magenta). This result contradicts the known ToL relationships and might suggest that these two ReGens have diverged from their original function as EAA biosynthesis enzymes. Less conserved *C*-terminal and *N*-terminal protein regions produced a smaller number of comparisons due to a higher number of gaps and were removed from the plot (original alignments are provided in Supplementary material 4). In a more detailed analysis, we focused on the highly conserved regions between plants and fungi in the ALS and BHMT proteins (Supplementary material 7). The Weblogo plots [49] show that fungi and plant proteins share a larger number of differentially conserved amino acids than the other pairwise clade comparisons in the selected regions.

**Table 2:** Organisms studied.

Organism Name	Clade
*Neurospora crassa OR74A*	Fungi
*Pyrenophora tritici-repentis*	Fungi
*Sclerotinia sclerotiorum 1980 UF-70*	Fungi
*Schizosaccharomyces pombe*	Fungi
*Gibberella zeae PH-1*	Fungi
*Aspergillus niger*	Fungi
*Fusarium oxysporum f.* sp. *lycopersici*	Fungi
*Puccinia graminis f.* sp. *Tritici*	Fungi
*Saccharomyces cerevisiae S288c*	Fungi
*Ustilago maydis 521*	Fungi
*Homo sapiens*	Metazoan
*Pan troglodytes*	Metazoan
*Mus musculus*	Metazoan
*Monodelphis domestica*	Metazoan
*Taeniopygia guttata*	Metazoan
*Anolis carolinensis*	Metazoan
*Xenopus tropicalis*	Metazoan
*Danio rerio*	Metazoan
*Drosophila melanogaster*	Metazoan
*Ciona intestinalis*	Metazoan
*Caenorhabditis elegans*	Metazoan
*Solanum tuberosum*	Plant
*Vitis vinifera*	Plant
*Populus trichocarpa*	Plant
*Arabidopsis thaliana*	Plant
*Physcomitrella patens*	Plant
*Chlamydomonas reinhardtii*	Plant
*Selaginella moellendorffii*	Plant
*Oryza sativa*	Plant
*Sorghum bicolor*	Plant
*Zea mays*	Plant

### 3.4. Phylogenies of ReGens Using Maximum Likelihood

Phylogenetic analysis was performed for all eight ReGens except AadAT (absent in plants), using maximum likelihood (ML) approaches based on the JTT method implemented in the PhyML package [17]. As most gene trees agree with the species tree, metazoan ReGens were expected to cluster with their fungi orthologs. If non-ToL evolution occurred in the metazoan genes, fungi and plant enzymes (still being used for amino acid biosynthesis and therefore subject to similar functional constraints) might retain a higher level of similarity. Thus, a phylogenetic analyses of these genes would result in the autotrophic (plant and fungi) genes being clustered as sister groups, while metazoan ones would be seen as an outgroup.

Corroborating the conservation analysis results, phylogeny data showed that most observed ReGens fit the ToL model, and metazoan proteins were found to cluster together with fungal proteins in the expected *Opisthokonta* clade. Table 3 resumes the tree topology information and Supplementary material 8 provides the ML trees with bootstrap values.

However, we found that the genes with considerable conservation between Fungi and Plants (Figure 2), such as ALS and BHMT, also showed tree topologies that did not support the *Opisthokonta* clade (Table 3), further supporting the hypothesis of non-ToL evolution. The BHMT and ALS trees showed the greatest ratio between the metazoan-fungi and fungi-plant relative branch lengths and represent clear examples of molecular anagenesis in the metazoan lineage. The ALS tree showed that most fungi proteins were found as a sister group of the plant clade, supporting an autotrophic paraphyletic group and suggesting non-ToL evolution of metazoan orthologs. Finally, the SD phylogenetic tree topology showed fungi as an outgroup, probably due to the existence of two distinct genes coding each of the functions.

**Table 3:** Summary of maximum likelihood (ML) tree topology.

Topology Name	Topology Schema	ReGen	mf/fp Branch Length Ratio
ToL-like topology	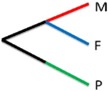	BCA	0.17
		CTH	0.31
		AATm	0.34
		AATc	1.30
Autotrophic paraphyly	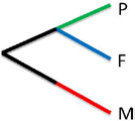	ALS	3.80
		BHMT	3.59
Fungi as outgroup	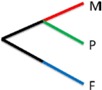	SD	0.73

### 3.5. Synonymous and Non-Synonymous Mutation Rates

Another standard metric for analyzing the effects of natural selection on genes and proteins is the ratio between synonymous (Ka) and non-synonymous (Ks) substitutions [50]. In this evaluation, Ka/Ks scores less than one indicate that the sequences under analysis were subject to purifying selection, while values close to one indicate neutral variation and scores greater than one are evidence of positive selection.

Here, all-against-all Ka/Ks ratios for all ReGens were calculated for each pair of species using PAML pairwise algorithms and then averaged within each clade. We found that both ALS and BHMT had the highest Ka ratios for comparisons involving the metazoan clade, suggesting a faster rate of evolution (Table 4) [51]. Ks estimation was saturated mainly because of the early divergence between the clades (>1 billion years). Thus, Ka/Ks could not be precisely estimated for the complete sequences of ReGens in this timescale.

We also performed a branch test with PAML using the maximum likelihood phylogenetic trees of the previous section (Table 4), in which we tested whether the Ka/Ks ratio for the metazoan branch was significantly higher than for the rest of the tree. We found *p*-values < 0.05 for ALS, BHMT and for both the cytoplasmic and mitochondrial orthologs of AAT.

**Table 4:** Ka/Ks ratio as calculated by PAML.

ReGen	Clades Compared	Color Code	Ka	Ks
ALS	*fp*	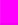	0.58	57.29
	*mf*	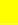	1.15	37.93
	*mp*	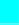	1.26	28.65
BHMT	*fp*	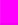	0.89	54.54
	*mf*	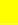	1.74	31.84
	*mp*	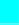	1.45	36.84
BCA	*fp*	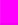	0.70	48.79
	*mf*	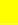	0.45	57.63
	*mp*	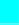	0.70	61.02
SD	*fp*	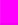	0.64	35.62
	*mf*	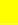	0.66	44.35
	*mp*	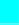	0.67	17.31
CTH	*fp*	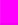	0.74	48.33
	*mf*	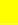	0.53	53.39
	*mp*	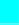	0.92	34.77
AATm	*fp*	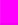	0.46	64.12
	*mf*	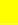	0.34	61.14
	*mp*	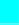	0.40	62.58
AATc	*fp*	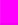	0.47	65.63
	*mf*	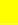	0.43	56.60
	*mp*	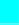	0.46	56.57

## 4. Discussion

Here we have performed a conservation study of all genes involved in EAA biosynthesis that remained in metazoan genomes long after the deletion of their pathway partners. We performed manual curation of the enzymes involved in biosynthetic pathways for EAA in autotrophic organisms [2], selected the eight homologous genes present in metazoan genomes and studied the evolutionary fate of seven of them (excluding AadAT). Why are these genes retained in the metazoan genomes if they no longer participate in amino acid biosynthesis? We used standard molecular evolution metrics and developed new strategies to understand the evolutionary fate of these remaining genes.

We have shown that five of the analyzed ReGens show evidence of standard ToL-like evolution in metazoans. These genes most likely act in the biosynthesis of other amino acids or can hypothetically be used together with metabolites provided by symbionts or commensals to complement the biosynthetic pathway. Interactions between proteins from hosts and symbionts have been shown in a number of organisms, sometimes in a given tissue or during a brief development stage [52,53].

We observed favored permanence of genes at the start or end of EAA pathways, possibly because these are usually responsible for the regulation of the entire pathway. Moreover, the last genes of a biosynthetic pathway might also take part in the degradation of the final product, as is the case with BHMT and SD.

On the other hand, several of our experiments suggested non-ToL evolution of the ALS and BHMT genes in metazoan genomes (Table 5). These results are further supported by the observation that amongst the 13 amino acids annotated as belonging to the catalytic site of *Arabidopsis thaliana*’s ALS protein structure (deposited as 1YI1 [54] in the PDB database [55] and verified using the Catalytic Site Atlas [56]), 10 were found to be conserved in fungi and only 5 in metazoan proteins. Further experimental studies on ALS might indicate whether this enzyme has neofunctionalized or not.

Concerning BHMT, we found a complex evolutionary history. The eukaryotic ancestor used *S*-methyl-l-methionine as a methyl donor, while the metazoan ancestror used betaine instead. The gene seems to be duplicated as BHMT and BMHT2 in the Therian clade, allowing BHMT2 to change its substrate from betaine back to *S*-methyl-l-methionine [42].

**Table 5:** Summary of results.

ReGen	Tree Topology *	Conservation Diagram	Ka/Ks Branch Test (*p* < 0.05)	Ka/Ks Clade Average > 1 for Metazoans
ALS	Non-ToL	Non-ToL	+	Yes
BHMT	Non-ToL	Non-ToL	+	Yes
BCA	ToL	ToL	−	No
SD	FO	ToL	−	No
CTH	ToL	ToL	−	No
AATm	ToL	ToL	+	No
AATc	ToL	ToL	+	No

* ToL, Tree of Life topology; FO, fungi outgroup

## 5. Conclusions

Eight of the 49 genes participating in the EAA biosynthetic pathways in autotrophs have been retained in metazoans (ReGens). Two of them (ALS and BHMT) show phylogenetic evidence, conservation profiles and non-synonymous mutation rates that suggest non-ToL evolution in metazoans.

## Supplementary Files

Supplementary File 1
