# Supplementary material for: Metazoan Remaining Genes for Essential Amino Acid Biosynthesis: Sequence Conservation and Evolutionary Analyses
Source: Nutrients. 2014 Dec 24;7(1):1–16. doi: 10.3390/nu7010001 (PMC4303824; doi:10.3390/nu7010001)
Supplement: Supplementary File 1 [file nutrients-07-00001-s001.docx]

Supplementary Information

**Supplementary Material 1**. Brief description of the conservation plot algorithm.


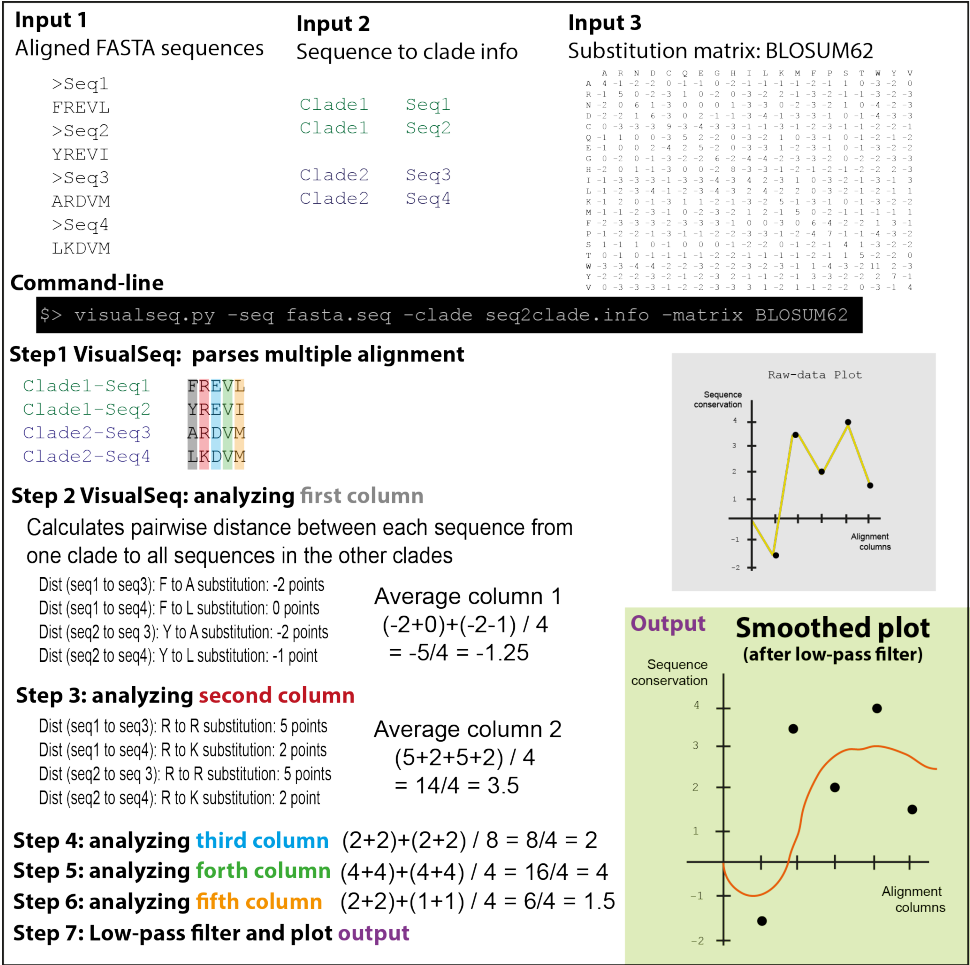


In order to present a visual analysis of the conservation of ReGens amongst the three clades studied (metazoans, fungi and plants), we developed a Python script named visualseq.py. This script counts amino acidic conservation amongst clades and plots an average conservation value allowing easy visualization. VisualSeq works using as input a FASTA file containing multiple sequence alignments and a sequence-to-clade file, describing which sequences in the FASTA file correspond to a given clade. VisualSeq observes each column of the alignment and calculates the pairwise distance between all sequences from a clade to all sequences from another clade tabulated by a substitution matrix. Here we used BLOSUM62 matrix.

**Supplementary Material 2**. Yeast enzymes for EAA biosynthesis. Names and identifiers for all enzymes involved in the metabolism of essential amino acids.

| **Amino Acid** | **Enzyme** | **Pathway Step** | **UniProt Accession Number** | **Found in Metazoans** |
| --- | --- | --- | --- | --- |
| Histidine | ATP phosphoribosyltransferase | 1 | P00498.1 | No |
|  | Histidine biosynthesis trifunctional protein | 2 | P00815.3 | No |
|  | Phosphoribosylformimino-5-aminoimidazole carboxamide ribotide isomerase | 3 | P40545.1 | No |
|  | Glutamine amidotransferase | 4 | P38625.4 | No |
|  | Imidazoleglycerol phosphate synthase | 5 | P06633.2 | No |
|  | Histidinol-phosphate aminotransferase | 6 | P07172.2 | No |
|  | Histidinol-phosphatase | 7 | P38635.2 | No |
|  | Histidinol dehydrogenase | 8 | P00815.3 | No |
| Lysine 1 | Homocitrate synthase | 1 | P48570.1; Q12122.1 | No |
|  | Homoaconitate hydratase | 2 | P49367.1 | No |
|  | Homoisocitrate dehydrogenase | 3 | P40495.1 | No |
|  | Aromatic/aminoadipate aminotransferase 1 | 4 | P53090.1 | Yes |
|  | Aminoadipate-semialdehyde dehydrogenase | 5 | P07702.2 | No |
|  | Saccharopine dehydrogenase  (NAD^+^. l-glutamate forming) | 6 | P38999.1 | Yes |
|  | Saccharopine dehydrogenase  (NAD^+^. l-lysine forming) | 7 | P38998.3 | No |
| Lysine 2 | Aspartate kinase | 1 | P10869.2 | Yes |
|  | Aspartate-semialdehyde dehydrogenase | 2 | P13663.1 | No |
|  | Dihydrodipicolinate synthase | 3 | only in plants | No |
|  | Dihydrodipicolinate reductase | 4 | only in plants | No |
|  | LL-diaminopimelate aminotransferase | 5 | only in plants | No |
|  | Diaminopimelate epimerase | 6 | only in plants | No |
|  | Diaminopimelate decarboxylase | 7 | only in plants | No |
| Isoleucine/Leucine/Valine | Threonine dehydratase | 1 | P00927.2 | No |
|  | Acetolactate synthase | 2 | P25605.2; P07342.1 | Yes |
|  | Ketol-acid reductoisomerase | 3 | P06168.1 | No |
|  | Dihydroxyacid dehydratase | 4 | P39522.2 | No |
| Isoleucine/Valine | Branched-chain amino acid aminotransferase | 5 | P47176.1; P38891.1 | Yes |
| Leucine | Alpha-isopropylmalate synthase | 5 | P06208.1 | No |
|  | Isopropylmalate isomerase | 6 | P07264.3 | No |
|  | Beta-isopropylmalate dehydrogenase | 7 | P04173.4 | No |
|  | Leucine aminotransferase | 8 | P53090.1; P47176.1; P38891.1 | No |

**Supplementary Material 2.** *Cont.*

| **Amino Acid** | **Enzyme** | **Pathway Step** | **UniProt Accession Number** | **Found in Metazoans** |
| --- | --- | --- | --- | --- |
| Methionine 1 | Serine *O*-acetyltransferase | 1 | P08465.2 | No |
|  | Cysteine synthase A | 2 | P53206.1 | No |
|  | Cystathionine gamma-lyase | 3 | P31373.2 | Yes |
|  | Cystathionine beta-lyase | 4 | P53101.1 | No |
|  | Betaine-homocysteine *S*-methyltransferase | 5 | P05694.4 | Yes |
| Methionine 2 | Aspartate kinase | 1 | P10869.2 | Yes |
|  | Aspartate-semialdehyde dehydrogenase | 2 | P13663.1 | No |
|  | Homoserine dehydrogenase | 3 | P31116.1 | No |
|  | Homoserine *O*-acetyltransferase | 4 | P08465.2 | No |
|  | Cystathionine gamma-synthase | 5 | P47164.1 | Yes |
|  | Cystathionine beta-lyase | 6 | P53101.1 | No |
|  | Betaine-homocysteine *S*-methyltransferase | 7 | P05694.4 | Yes |
| Phenylalanine | 3-Deoxy-d-arabinoheptulosonate 7P-synthase | 1 | P32449.2 | No |
|  | Dehydroquinate synthase | 2 | P08566.1 | No |
|  | 3-Dehydroquinate dehydratase | 3 | P08566.1 | No |
|  | Shikimate dehydrogenase | 4 | P08566.1 | No |
|  | Shikimate kinase | 5 | P08566.1 | No |
|  | 3-Phosphoshikimate 1-carboxyvinyltransferase | 6 | P08566.1 | No |
|  | Chorismate mutase | 7 | P32178.1 | No |
|  | Chorismate synthase | 8 | P28777.1 | No |
|  | Prephenate dehydrogenase | 9 | P20049.2 | No |
|  | 4 Aminotransferases | 10 | P23542.3; Q01802.2; P07172.2; | Yes |
| Threonine | Aspartate kinase | 1 | P10869.2 | Yes |
|  | Aspartate-semialdehyde dehydrogenase | 2 | P13663.1 | No |
|  | Homoserine dehydrogenase | 3 | P31116.1 | No |
|  | Homoserine kinase | 4 | P17423.4 | No |
|  | Threonine synthase | 5 | P16120.1 | No |
|  | Threonine aldolase | 6 | P37303.2 | No |
| Tryptophan | 3-Deoxy-d-arabinoheptulosonate 7-phosphate synthase | 1 | P32449.2 | No |
|  | Dehydroquinate synthase | 2 | P08566.1 | No |
|  | 3-Dehydroquinate dehydratase | 3 | P08566.1 | No |
|  | Shikimate dehydrogenase | 4 | P08566.1 | No |
|  | Shikimate kinase | 5 | P08566.1 | No |
|  | 5-Enolpyruvylshikimate 3-phosphate synthase | 6 | P08566.1 | No |
|  | Chorismate synthase | 7 | P28777.1 | No |
|  | Anthranilate synthase | 8 | P00899.4; P00937.2 | No |
|  | Anthranilate phosphoribosyltransferase | 9 | P07285.1 | No |
|  | *N*-(5’-phosphoribosyl)-anthranilate isomerase | 10 | P00912.2 | No |
|  | Indole-3-glycerol phosphate synthase | 11 | P00937.2 | No |
|  | Tryptophan synthase | 12 | P00931.1 | No |

**Supplementary Material 3**. BHMT and BHMT2 phylogenetic tree.


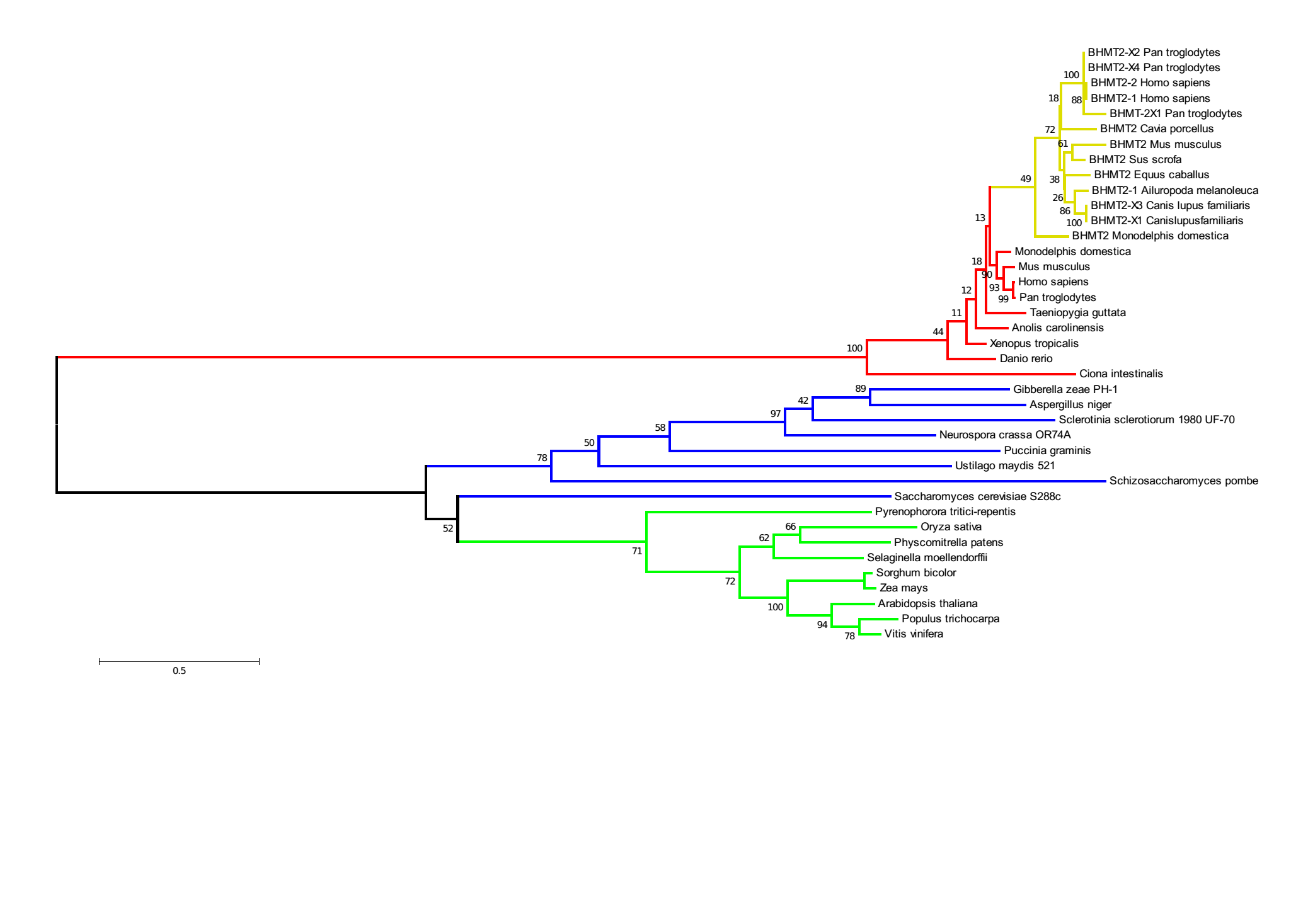


**Supplementary Material 4**. ClustalW alignment of ReGens in FASTA format.

**ALS.fasta**

>Gibberella_zeae_PH-1

---MLRSRP-TRQAIRAFG-------HARAR---TTLPAT---------QRTKATATT--

-------------------SAAPQTRPVPSPAFNLEPESRRH--------VQPLVNR---

-SNPEMDESFIGKSGGEIFHEMMLRHDVKHIFGYPGGAILPVFDAIYNSKHFDFILPRHE

QGAGHMAQGYARATGKPGVVLVTSGPGATNVITPMQDALSDGTPIVVFTGQVVTSAIGSD

AFQECDTVGISRSACKWNCMVTSIAELPRRINEAFEIATSGRPGPVLIDLPKDITAGILR

KAIPTQT----------------RLPSIPSMASQTAKQVMEKQ---------LNASLSRA

ADLIKIAKQPIIYAGQGIIQS-EGGTELLRELADKCSIPVTTTLQGLGAFDERDEKSLQM

LGMHGMAYANMSMQEADLIIALGARFDDRVVLNTSKFAPNAKAAAAEKRGGIIHFDILPK

NINKVIQATEAIEGDVATNLKMLMPMLTPT--S-MEQRSGWFNKISEWKAKWPLSNYEHS

DAPGGSGLIKPQTLIEELS---DLTSKIDKKTIISTGVGQHQMWVAQHYLWREPRSFVTS

GGLGTMGFGLPSAIGIAVAK------PDALVIDIDGDASFNMTLTELSTAAQFNIGVKVI

ILNNEEQGMVTQWQNLFYEDRYAHTHQ---------------KNPDFIKLADAMGVQARK

VTKPDDVRASLEWLINTDG--PALLEVVTDKKVPLLPMVPAGAGLHEFLVYDKAFDYREG

QGSTSANARANQWSSRFLIKK----------

>Sclerotinia_sclerotiorum_1980_

---MLRSIP-ARRAIRTIGSNSRWMEVAGARGLSNRVKAAGGIMVEKKGTRGQATAAP--

-------------------QVSNQARESPSPAFNNEPSKNDS--------RKELTGQGH-

-KYSEMDESFIGMTGGEIFHEMMLRQDVKHIFGYAGGAILPVFDAIYNSKHFEFILPKHE

QGAGHMAEGYARACGKPGVVLVTSGPGATNVITPMQDALSDGTPMVVFTGQVVTTAIGSD

AFQEADIVGISRACTKWNVMVKNIAELPRRIKEAFEIATSGRPGPVLVDLPKDVTAGILR

KAIPSHH----------------IMPSLPSQASRAAQAIQKKQ---------LQASIDRV

AKLINIAKKPIIYAGQGVISL-PEGPKLLKELADKASIPVTTTLQGLGGFDELDEKSLHM

LGMHGSAYANLSMQEADLIIALGARFDDRVTGSIPKFAPAAKAAAAEGRGGIVHFEIMPK

NINKVVQATEAVEGDVVTNLAQLLPAVEKK--S-MDERKEWFDQIRFWKEKFPLSHYEKA

EP---DGLIKPQTLIEELS---NLTADRKHETIIATGVGQHQMWTAQHFRWRHPRTMITS

GGLGTMGYGLPAAIGAKVAK------PDALVIDIDGDASFNMTLTELSTASQFNIGVKVI

VLNNEEQGMVTQWQNIFYQDRYAHTHS---------------TNPDFMKLADSMGVQHRR

CIKPDDVRDSLKWLIDSDG--PALLEVVTDKKVPVLPMVPAGNALHDFLVYDPVKDKERR

KMIKERTCGLHG-------------------

>Neurospora_crassa_OR74A

---MLRSRQ-AARALKALT-------EAQHISQRTTLSASQRAAVLLTNRSVQTRNQS--

-------------------TAAAATRPTPSQHFQPEPTPSN---------VQPLAGR---

--KPDMDESFIGKTGGEIFHEMMLRHGVKHIFGYPGGAILPVFDAIYNSPHFDFVLPRHE

QGAGHMAEGYARASGKPGVVLVTSGPGATNVVTPMADALADGTPMVVFSGQVPTTAIGSD

AFQEADVIGISRACTKWNVMVKSVAELPRRINEAFEIATSGRPGPVLVDLPKDITAGILR

RAIPTDT----------------AIPTSPSAASRAAIELSRKQ---------LDASIQRV

AKLVNIAKKPVIYAGHGVVQS-KGGPALLRALSEKASIPVTTTLHGLGAFDELDEKSLHM

LGMHGAAYANMAVQEADLIICLGGRFDDRVTLNLNKFAPAAKAAAAEGRGGIVHFEILPK

NINKVVQATEAVEGDVATNIELLIPQVDAK--T-MADRKEWFGKINEWKSKWPLSDYERA

ER---TGLIKPQTLIEELS---KLTEGRKENTYIATGVGQHQMWTAQHFRWRHPRTMITS

GGLGTMGFGLPAAIGAKVAK------PDALVIDIDGDASFGMTLTELSTAAQFNIGVKVI

VLNNEEQGMVTQWQNLFYDDRYSHTHQ---------------KNPDFVKLADAMGVQSKR

IIKPEEVVEGLKWLIDSEG--PALLEVVTDKKVPVLPMVPAGCGLDEFITFDQERDRKRR

ELMVKRTGGVHGL------------------

>Pyrenophora_tritici-repentis

---MQQSRTRAVNALKGTRNFSTTAQRCGISPFRRPAKVVPSKVTKDEAKRPQSTAAA--

-------------------ATQTQSRSRPSPAFNRE--DYSE--------VQPLKQYR--

--SPAMDHSFVGMTGGEIFHEMMLRQGVKHIFGYPGGAILPVFDAIYQSKHFDFILPRHE

QGAGHMAEGYARASGKPGVVLVTSGPGATNVVTPMQDALMDGTPLIVFCGQVVTSAIGSD

AFQEADMIGITRPCTKWNVLVKNVAELPRRINEAFEIATSGRPGPVLVDLPKDVTASVLQ

RAIPMSS----------------SLPTTVSAATIAARELSKQQ---------LDNSIRRS

ANLINMAKKPVIYAGQGIMQT-ENGPQLLKELADKVNIPVTTTLQGLGGFDELDPKSLHM

LGMHGSAYANMAMQEADLILALGARFDDRITGAISKFAPAAKAAAAEGRGGIIHFEIMPK

NINKVIQATEAVEGDLGVNLKHLLPLVN----K-VEDRPEWLGQIAAWKERFPWAYEKEG

EN----GLIKPQTVMETLS---NLTADRKEETILTTGVGQHQMWCAQHYRWRYPRTMITS

GGLGTMGYGLPAAIGAKVAR------PDCMVIDIDGDSSFSMTLTELSTAAEFNIGVKVI

ILNNEEQGMVTQWQTLFYTDRFAHTHQ---------------RNADFVKLGDAMGVQAKR

CTKPDELEASLKWLLATDG--PALLEIVTDQKVPVLPMVPAGSALHEFMVYDEKATKERR

AQTKSRSGR----------------------

>Aspergillus_niger

---MMPMRP-SKSAMRALHYQR---YIASGRMTFTTASVAATAPHRFSAQKRFQSTAT--

-------------------APAEGTRPVPSPAFNQEP-HRNE--------VSPLQNRQ--

--VPELDDSFVGLSGGEIFHEMMLRLGVKHVFGYPGGAILPVFDAIYNSKHFDFVLPRHE

QGAGHMAEGYARASGKPGVVLVTSGPGATNVITPMQDALSDGTPMVVFCGQVPTSLIGTD

SFQEADVIGISRACTKWNVMVKSVAELPRRIQEAFEIATSGRPGPVLVDLPKDITAGILR

KPIPMNS----------------TLPSLPSAATMAARELSMQQ---------LQGTINRV

ARLVNVSKKPILYVGQGLLAR-PEGPQILKELADKACIPVTTTLQGLGGFDETDPKALHM

LGMHGSAYANMAMQEADLIIAIGARFDDRVTGNISKFAPQAKLAASENRGGIVHFEIMPK

NINKVVQANEAVEGDCAENIRLLLPHVEP-----VAERKEWFEQINDWKARFPFSLYEKQ

TA---EGPIKPQAVIEKLS---DLTAHMKDRTVITTGVGQHQMWAAQHFRWRHPRTMITS

GGLGTMGYGLPAAIGAKVAR------PDALVVDIDGDASFNMTLTELTTAAQFNIGVKVL

LLNNEEQGMVTQWQNLFYEDRYSHTHQ---------------KNPDFVPMAQSMGVAADR

CTKPSEVEAKLKWLIEQDG--PALLEVFTDRKVPVLPMVPAGCALHEFLVYDEAKEKERK

ALMKKRKVPGF--------------------

>Saccharomyces_cerevisiae_S288c

---MIRQSTLKNFAIKRCF--------QHIAYRNTPAMRSVALAQRFYSSSSRYYSAS--

-------------------PLPASKRPEPAPSFNVDPLEQPAE-------PSKLAKKLR-

-AEPDMDTSFVGLTGGQIFNEMMSRQNVDTVFGYPGGAILPVYDAIHNSDKFNFVLPKHE

QGAGHMAEGYARASGKPGVVLVTSGPGATNVVTPMADAFADGIPMVVFTGQVPTSAIGTD

AFQEADVVGISRSCTKWNVMVKSVEELPLRINEAFEIATSGRPGPVLVDLPKDVTAAILR

NPIPTKT----------------TLPSN--ALNQLTSRAQDEF---------VMQSINKA

ADLINLAKKPVLYVGAGILNH-ADGPRLLKELSDRAQIPVTTTLQGLGSFDQEDPKSLDM

LGMHGCATANLAVQNADLIIAVGARFDDRVTGNISKFAPEARRAAAEGRGGIIHFEVSPK

NINKVVQTQIAVEGDATTNLGKMMSKIFP-----VKERSEWFAQINKWKKEYPYAYMEET

PG----SKIKPQTVIKKLS---KVANDTGRHVIVTTGVGQHQMWAAQHWTWRNPHTFITS

GGLGTMGYGLPAAIGAQVAK------PESLVIDIDGDASFNMTLTELSSAVQAGTPVKIL

ILNNEEQGMVTQWQSLFYEHRYSHTHQ---------------LNPDFIKLAEAMGLKGLR

VKKQEELDAKLKEFVSTKG--PVLLEVEVDKKVPVLPMVAGGSGLDEFINFDPEVERQQT

ELRHKRTGGKH--------------------

>Ustilago_maydis_521

---MTSFAVQFARQQRHAFQAASKFSAASTSSIAVSAAASTSYASTPCATRAFSTARNNL

SRSAQLARSQRSQLALTRHISTNQPKEQAAAAAQTAASVRPEPSPAFQQSPQNIAPLASR

YDANALDESFVGLSGGAIFHEMMLRHNVKHVFGYPGGAILPVFDAIYNSKHFDFVLPRRE

DGAGHMAEGYARVTGKPGVILVTSGPGATNVITPMQDAMSDGTPLVVFCGQVATSAIGSD

AFQEADVVGISRSCTKWNVMVKDIAELPRRINEAFKIATSGRPGPVLVDLPKDVTAGILR

QAIPYSQTQ--------PNMIT-NLPSRQVARNRRGATFQQSANLRSSEQDILTSELQEA

ARLIGIAKRPIIYAGQGILSS-PEGPKLLAKLAKEHNIPVTTTLQGLGAFDELDSLSLHM

LGMHGSAYANLAMQDADLIIALGARFDDRVTGKIDKFAPHARAAALENRGGIIHFEVMPK

NINKVVQATCAIEGDVVTNLGKLMPYL--K--SNAPDRSEWHQLIKSWKEAYPFTYEPSK

EG----ELMKPQEVIEALD---AAVADQKDNVIISTGVGQHQMWAAQHYRWRHPRTWVSS

GGLGTMGFGLPSCIGAKVAA------PEKIVVDIDGDASFSMTAMELATAAQYNIGVKVL

VLNNEFQGMVVQWQDLFYEKRYSHTEM---------------HNPDFVKLAESMGVKAIR

CDTIAELPAKMKEFIEYDNNKPVLFEARITKNEHVYPFCPANAGLHELIVHPSLKPVPKR

S------------------------------

>Puccinia_graminis

MIKPTKLTSNLILNSRQNIKLPSILNLHHQTPASPSPSPTSASASSSYSHRSISSTNS--

--------------------KSNLPRPNPAPAFQQKPSTLTTPTP----TPQPLNQQTSI

INGEVMDHSFIGMSGGQIFHEMMLRHNVKHVFGYPGGAILPVFDAIHNSKHFDFILPRHE

QGAGHMAEGYARVSGKPGVVLVTSGPGATNTITPLQDAYSDGTPLIVFTGQVATSAIGSD

AFQEADVIGISRSCTKWNVMVKDVTDLPRRINEAFKIATSGRPGPVLVDLPKDMTAGILR

TPIPLKYTQ--------PDLIERVLPSNPLRSRPWSSSPDQTPNSKSS--SFETRELRQA

AQMILNAKRPVIYAGQGILSS-DDGPKLLRQLADQWNIPVTTTLMGMGAFDEEDPKSLHM

LGMHGSAYANLAMQDADVLIALGARFDDRVTGKVNTFAPHALAAAQQGTGGIIHFEIQPK

NINKVVEATCSVEGDVIQSLTHLLNILPAR--SSPPERREWLETIQGWKEKYPFVYEPSE

SS----EAIKPQEVIEALNDWSKAVPGRKDKLVISTGVGQHQMWAAQHYRWTAPRSIVTS

GGLGTMGYGLPAAIGAKVAR------PDQFVVDIDGDASFSMTAMELATAHEFNIGVKVI

VLDNEFQGMVLQWQDLFYESRYSGTRM---------------TNPDFAQLAKAMRCHAIY

CDQKDDLKAKIDELMAFDNSRPVLLHVKVTDKEHCFPMVPAGKALHEQILHPILRKKTP-

-------------------------------

>Schizosaccharomyces_pombe

---MTVLAPLRRLHTRAAF---------SSYGREIALQKRFLNLNSCSAVRRYGTGFS--

---------------------NNLRIKKLKNAFGVVRANSTKS-------TSTVTTAS--

--PIKYDSSFVGKTGGEIFHDMMLKHNVKHVFGYPGGAILPVFDAIYRSPHFEFILPRHE

QAAGHAAQAYSRVTKKPGVVLVTSGPGATNVITPIADALADGTPLVVFSGQVATSAIGSD

AFQEADMVGISRSCTKWNVMVKDVADLPRRIDEAFEIATSGRPGPVLVDLPKDVTASVLK

EPIPILS----------------SVPSMNRRMKEVLEEGSKNV----------TAKIDRV

ANLLKLAKKPVIFCGHGVLAN-PECPTLLRKFSERLQIPVTTSLLGLGAVDERSDLSLHM

LGMHGSGYANMAMQEADLILALGVRFDDRVTGNVSLFAPQARLAAAEERGGIIHFDISPK

NIGKVVQPTEAIEGDVYESLKLLDSATKNI--K-IPSRFDWLSQIQTWKERFPFTFTRSA

PG----ELVKPQEVIQELD---KQTSDIKDKVTITTGVGAHQMWAATFYRWTKPSSLVTS

GGLGTMGFGLPAAIGASVAA------PKDIVIDIDGDASFSMTGMELATVRQFDIPVKIL

ILNNEEQGMVTQWQNLFYEKRYSHTHQ---------------KNPNFVKLADAMGIKALR

VEKREDLAKKMKEFLSTKG--PVLMEVLVAQKEHVYPFVPGGKALHQFILHESLS-----

-------------------------------

>Zea_mays

------------------------------------------------------------

------------------------------------------------------------

---------------------------------------MEIHQALTRSPVIANHLFRHE

QGEAFAASGYARSSGRVGVCIATSGPGATNLVSALADALLDSVPMVAITGQVPRRMIGTD

AFQETPIVEVTRSITKHNYLVLDVDDIPRVVQEAFFLASSGRPGPVLVDIPKDIQQQMAV

PVWDKPMS----------------LPGYIARLPKPPAT----------------ELLEQV

LRLVGESRRPVLYVGGGCAAS-GEE---LRRFVELTGIPVTTTLMGLGNFPSDDPLSLRM

LGMHGTVYANYAVDKADLLLALGVRFDDRVTGKIEAFASRAK---------IVHVDIDPA

EIGKNKQPHVSICADVKLALQGMNALLEGSTSKKSFDFGSWNDELDQQKREFPLGYK--T

SN----EEIQPQYAIQVLD------ELTKGEAIIGTGVGQHQMWAAQYYTYKRPRQWLSS

AGLGAMGFGLPAAAGASVAN------PGVTVVDIDGDGSFLMNVQELAMIRIENLPVKVF

VLNNQHLGMVVQLEDRFYKANRAHTYLGNPEN-------ESEIYPDFVTIAKGFNIPAVR

VTKKNEVRAAIKKMLETPG--PYLLDIIVPHQEHVLPMIPSGGAFKDMILDGDGRTVY--

-------------------------------

>Oryza_sativa

----MATTAAAAAAALSAAATAKTGRKNHQRHHVLPARGRVG------------------

-----------------------------AAAVRCSAVSPVTP-------PSPAPPATP-

---LRPWGPAEPRKGADILVEALERCGVSDVFAYPGGASMEIHQALTRSPVITNHLFRHE

QGEAFAASGYARASGRVGVCVATSGPGATNLVSALADALLDSVPMVAITGQVPRRMIGTD

AFQETPIVEVTRSITKHNYLVLDVEDIPRVIQEAFFLASSGRPGPVLVDIPKDIQQQMAV

PVWDTSMN----------------LPGYIARLPKPPAT----------------ELLEQV

LRLVGESRRPILYVGGGCSAS-GDE---LRWFVELTGIPVTTTLMGLGNFPSDDPLSLRM

LGMHGTVYANYAVDKADLLLAFGVRFDDRVTGKIEAFASRAK---------IVHIDIDPA

EIGKNKQPHVSICADVKLALQGLNALLQQSTTKTSSDFSAWHNELDQQKREFPLGYK--T

FG----EEIPPQYAIQVLD------ELTKGEAIIATGVGQHQMWAAQYYTYKRPRQWLSS

AGLGAMGFGLPAAAGASVAN------PGVTVVDIDGDGSFLMNIQELALIRIENLPVKVM

VLNNQHLGMVVQWEDRFYKANRAHTYLGNPEC-------ESEIYPDFVTIAKGFNIPAVR

VTKKSEVRAAIKKMLETPG--PYLLDIIVPHQEHVLPMIPSGGAFKDMILDGDGRTVY--

-------------------------------

>Sorghum_bicolor

------------------------------------------------------------

------------------------------------------------------------

---------------------------------------MDLRKSD--------------

-----------------GVFTVPPAPEAG---------LSLPPPPLLILRAVP-------

-----PAVSLCCGS--------------RVGQEAFFLASSGRPGPVLVDIPKDIQQQMAV

PVWDTPMS----------------LPGYIARLPKPPAT----------------ELLEQV

LRLVGESRRPVLYVGGGCAAS-GEE---LRRFVEMTGIPVTTTLMGLGNFPGDDPLSLRM

LGMHGTVYANYAVDKADLLLAFGVRFDDRVTGKIEAFASRAK---------IVHIDIDPA

EIGKNKQPHVSICADVKLALQGMNALLEGSTSKKSFDFGSWQAELDQQKREFPLGYK--T

FD----DEIQPQYAIQVLD------ELTKGEAIIATGVGQHQMWAAQYYTYKRPRQWLSS

AGLGAMGFGLPAAAGAAVAN------PGITVVDIDGDGSFLMNIQELAMIRIENLPVKVF

VLNNQHLGMVVQWEDRFYKANRAHTYLGNPEN-------ESEIYPDFVTIAKGFNIPAVR

VTKKSEVHAAIKKMLETPG--PYLLDIIVPHQEHVLPMIPSGGAFKDMILDGDGRTVY--

-------------------------------

>Physcomitrella_patens

------------------------------------------------------------

------------------------------------------------------------

---MSRYADDEPRKGSDILVEALEREGVRTTFAYPGGASMEIHQALTRSNVIRNVLCRHE

QGEIFAAEGYAKSSGRVGVCIATSGPGATNLVTGFADALLDSVPLVAITGQVPRRFIGTD

AFQETPIVEVTRSITKHNYLVMTVEDIPRVIREAFFLAASGRPGPVLVDIPKDIQQQMAI

PDWNQPMK----------------LQSYLSRLPPPPQI----------------SLMQQI

IRLLATAKKPVIYSGGGCLHA-SKE---LREFVELTGIPVTSTLMGLGTFPASDKRYLSM

LGMHGTVYANYAVDNADMLLAFGVRFDDRVTGKLESFASRAS---------IVHIDIDPA

EIGKNKQPHISICADVQLALAGLNKLIKEG-PAERPDFSAWRAELDGVKLKWPMKFP--K

FDN---SVIVPQLAIQTLC------ELTGGNAIISTGVGQHQMWAAQWYEYEQPRRWLTS

GGLGAMGFGLPSALGAAATH------PDIPVVDIDGDGSFIMNIQELATIHVEKLPVKIM

ILNNQHLGMVVQWEDRFYKANRAHTYLGDPEA-------EADIYPDFCKIAEGCKVPSAR

VTRREHLRDAITKMLETPG--PYLLDVIVPHQEHVLPMIPGGASFKEIITEGDGRQIY--

-------------------------------

>Selaginella_moellendorffii

------------------------------------------------------------

---------------------------------MAPFSFFITN-------EASPPASLF-

---QSRYGDSEPRKGADILVEALERQGVDIVFAYPGGASMEIHQALTRSTCIRNVLCRHE

QGEVFAAEGYAKASGKVGVCIATSGPGATNLVTGLADAALDSVPMVAITGQVPRRMIGTD

AFQETPIVEVTRSITKHNYLVMNVEDIPRVIEEAFFLASSGRPGPVLVDIPKDVQQQFAI

PNWSQPIK----------------LNAYMQRLPRFPEV----------------AQLQQI

VRLIANSKRPVVYCGGGCMNS-SEE---VREFVKLTGIPITMTLMGLGIFPQSDSRSLHM

LGMHGTVYANYAVDGADLLLAFGVRFDDRVTGKLEAFASRAS---------IVHIDIDPA

EIGKNKQPHVSLCADVKPALQSLNLLLRE--QKVKPDVGSWIAELEEQKLKWPLKYV--E

FGD---DCIAPQHAIQTLC------EITQGNAIISTGVGQHQMWAAQWFKYDKPRRWLTS

GGLGAMGFGLPSAIGAAATF------PDVPVIDIDGDGSFLMNVQELATIHVEKLPVKIM

VLNNQHLGMVVQWEDRFYKANRAHTYLGDPDH-------EDEIFPDFLKFAEGCKIPCAR

VTKRSNLKAAIQKMLDTPG--PYLLDVIVPHQEHVLPMIPGGSSFKDIITEGDGRKTY--

-------------------------------

>Populus_trichocarpa

----MAAAAAATSTS-----TTPIRKPSSIYPLP-ISKFTLPFAFNPKKPCS--------

---------------------------LHISNSLSKPASTTTA-------SPATTEKFS-

---FSRFAPDQPRKGADILVEALEREGVKDVFAYPGGASMEIHQALTRSNIIRNVLPRHE

QGGVFAAEGYARASGLPGVCIATSGPGATNLVSGLADALLDSVPIVAITGQVPRRMIGTD

AFQETPIVEVTRSITKHNYLVLDVDDIPRIVREAFFLATSGRPGPVLIDIPKDIQQQLAV

PNWDVPMK----------------LPGYLSRLPKNPNE----------------LHLEQI

VRLISESKKPVLYVGGGCLNS-SEE---LRRFVELTGIPVASTLMGLGAFPVGDELSLQM

LGMHGTVYANYAVDKSDLLLAFGVRFDDRVTGKLEAFASRAK---------IVHIDIDSA

EIGKNKQPHVSVCGDVKVALQGMNKILESRGAKGKMDFRAWREELNEQKMNNPLSFK--T

FG----EAIPPQYAIQVLD------ELTDGNAIVSTGVGQHQMWAAQFYKYKRPRQWLTS

GGLGAMGFGLPAAIGAAVAN------PDAIVVDIDGDGSFIMNVQELATIRVENLPIKIM

LLNNQHLGMVVQWEDRFYKANRAHTYLGDPSN-------ESEIFPNMLKFADACGIPAAR

VTRKDDLRAAIQKMLDTPG--PYLLDVIVPHQEHVLPMIPSGGAFKDVIIEGDGRRKY--

-------------------------------

>Arabidopsis_thaliana

----MAAATTTTTTSSSISFSTKPSPSSSKSPLP-ISRFSLPFSLNPNKSSSSSRRRGI-

-------------------KSSSPSSISAVLNTTTNVTTTPSP-------TKPTKPETF-

---ISRFAPDQPRKGADILVEALERQGVETVFAYPGGASMEIHQALTRSSSIRNVLPRHE

QGGVFAAEGYARSSGKPGICIATSGPGATNLVSGLADALLDSVPLVAITGQVPRRMIGTD

AFQETPIVEVTRSITKHNYLVMDVEDIPRIIEEAFFLATSGRPGPVLVDVPKDIQQQLAI

PNWEQAMR----------------LPGYMSRMPKPPED----------------SHLEQI

VRLISESKKPVLYVGGGCLNS-SDE---LGRFVELTGIPVASTLMGLGSYPCDDELSLHM

LGMHGTVYANYAVEHSDLLLAFGVRFDDRVTGKLEAFASRAK---------IVHIDIDSA

EIGKNKTPHVSVCGDVKLALQGMNKVLENRAEELKLDFGVWRNELNVQKQKFPLSFK--T

FG----EAIPPQYAIKVLD------ELTDGKAIISTGVGQHQMWAAQFYNYKKPRQWLSS

GGLGAMGFGLPAAIGASVAN------PDAIVVDIDGDGSFIMNVQELATIRVENLPVKVL

LLNNQHLGMVMQWEDRFYKANRAHTFLGDPAQ-------EDEIFPNMLLFAAACGIPAAR

VTKKADLREAIQTMLDTPG--PYLLDVICPHQEHVLPMIPSGGTFNDVITEGDGRIKY--

-------------------------------

>Vitis_vinifera

----MASTAASAAANLS---ISKPSPTLSFQSLTSHSRLILPGSKAKITVSVAHR-----

-------------------ITASLSDPKPKPSSAVSASISTAA-------PVATSSETF-

---ISRFAPDEPRKGCDVLVEALEREGVKHVFAYPGGASMEIHQALTRSNIIRNVLPRHE

QGGVFAAEGYARASGRPGVCIATSGPGATNLVSGLADALLDSIPIVAITGQVPRRMIGTD

AFQETPIVEVTRSITKHNYLVLDVEDIPHIVREAFFLATSGRPGPVLIDVPKDIQQQLVV

PNWNKGLR----------------LPGYTFRLPKSPEK----------------AHLEQI

VRLISESKRPVLYVGGGCMNS-SEE---LKRFVDLTGIPVASTLMGLGAFPCTDKLSLQM

LGMHGTMYANYAVDRSDLLLAFGVRFDDRVTGKIEAFASRAK---------IVHIDIDPA

EIGKNKQPHVSVCADVKQALTGMNSLLEETGAKSKYDFSSWRDELNEQKAKYPLSFK--T

FG----EAIPPQYAIQVLD------ELTDGNAIISSGVGQHQMWAAQFYKYRGPRQWLSS

SGLGAMGFGLPAAMGAAVAK------PDAVVVDIDGDGSFIMNVQELATIRVENLPVKIM

LLNNQHLGMVVQWEDRFYKANRAHTYLGNPSN-------EAEIFPNMLKFAEACDIPAAR

VTKKSEVREAIKKMLETPG--PYLLDVIVPHQEHVLPMIPSGGAFKDAINEGDGREAY--

-------------------------------

>Chlamydomonas_reinhardtii

---MKALRSGTAVARGQAGCVSPAPRPVPMSSQAMIPSTSSPAARAPARSGRRALAVSA-

-------------------KLADGSRRMQSEEVRRAKEVAQAA-------LAKDSPADW-

---VDRYG-SEPRKGADILVQALEREGVDSVFAYPGGASMEIHQALTRSDRITNVLCRHE

QGEIFAAEGYAKAAGRVGVCIATSGPGATNLVTGLADAMMDSIPLVAITGQVPRRMIGTD

AFQETPIPRTSSSS---------------------WRCRTG-------------------

---SAPMS----------------ITGYISRLPPPVEE----------------SQVLPV

LRALQGAAKPVIYYGGGCLDA-QAE---LREFAARTGIPLASTFMGLGVVPSTDPNHLQM

LGMHGTVFANYAVDQADLLVALGVRFDDRVTGKLDAFAARAR---------IVHIDIDAA

EISKNKTAHVPVCGDVKQALSHLNRLLAAEPLPAD-KWAGWRAELAAKRAEFPMRYP--Q

RD----DAIVPQHAIQVLG------EETQGEAIITTGVGQHQMWAAQWYPYKETRRWISS

GGLGSMGFGLPAALGAAVAFDGKNGRPKKTVVDIDGDGSFLMNVQELATIFIEKLDVKVM

LLNNQHLGMVVQWEDRFYKANRAHTYLGKRESEWHATQDEEDIYPNFVNMAQAFGVPSRR

VIVKEQLRGAIRTMLDTPG--PYLLEVMVPHIEHVLPMIPGGASFKDIITEGDGTVKY--

-------------------------------

>Danio_rerio

--------------------------------------------MDISMYLGCSLG----

------------------------AALGGVIFASYKLGLLYQL-----------------

---FHKTERQSPRHGGESVAEVLRSHGVKFVFTLVGGHISPILVACEKLG-IRIVDTRHE

ATAVFAADAVARLSGTVGVAAVTAGPGLTNTVTAVKNAQMAESPLLLIGGAAATLLQGRG

ALQDIDQMSLFKPLCKFCASVRTVREIVPTVRKALAIAQSGTPGPVFIEFPIDTLYPYHV

VEKEFAPKN--TPKGLMGKIIAWYLKNHLSNLFAGAWESRDLSPLPVHIPHATDDQVQRC

VELVSRAKKPVILLGSQATLP-PTPADDIRKALESLGIPCFLGGMSRGLLGKNSPLHIRQ

NRRDALKDADLVLLAG-TVCDFRLSYGRVLNRRSKIIAVNRD------------KSQLLK

NSDMFWKPTVAIQGDAGSFLLNLSKALKG--HRCPEEWPQSLKEGDNVKEKANRAKADEK

TER----HLNPLSVLHRVD------ELLAEDSIIVADGGDFVGSAAYIMRPRGPLCWLDP

GAFGTLGVGGGFALGAKLCR------PESEVWIVYGDGSLGYTVAEFDTFTRHKTPVIAL

VGNDACWSQISREQVPMLGSNVACGLA----------------FTDYHVVADGYGGKGYL

IGR--EDESQLEDIIKKAQ-----KECKEGK-AVLLNVLIGKTNFREGSISV--------

-------------------------------

>Anolis_carolinensis

--------------------------------MNPDTIASASALGGTTTWLVAVLG----

------------------------TILVSLLGAAHKLGLIYQL-----------------

---WHQVDPKSTRHGGEIVAEVLKAHGVRYVFTLVGGHISPILVASEKLG-IRIVDTRHE

ATAVFAADAVARLSGTVGVAAVTAGPGVTNTITAVKNAQMAESPVLLIGGAAATLQKGRG

ALQDIDQLSLLKPLCKFCASVKSVRDISPVLRKAIAVAQCDTPGPVFVEFPLDVLYPYHL

IERELIKKT--TSKGVLGKAVNWYLRSYLGNLFAGAWAVRDLSPLPVKIPKATKKEVQQC

VELISRAKKPVILLGSQVTLP-PTPVKKLREALEELGIPCFLGGMARGMLGKNSPLHIRQ

NRREALKEADVVILAG-TVCDFRLSYGRILSKGSKIIAVNRN------------REQLLK

NAHIFWKPHVAIKGDAGSFLVHLSQELRG--YSCPRDWAISLKETDHVKEKENREKAEEP

TEH----HLNPLKLLHHVD------CLLPEDSLLVADGGDFVASAAYIVKPRGPLCWLDP

GAFGTLGVGGGFALGAKLCR------PESEVWILYGDGSLGFSVMEFDTFVRHKTPVIAL

VGNDACWSQISREQVPMLGSNVACGLD----------------YLDYHKVAEGLGGKGFL

LDR--QNEDQIDDIIRTAQ-----SGCRSGK-PFLLNALIGKTNFREGSISV--------

-------------------------------

>Homo_sapiens

---------------------------------METPAAAAPAGSLFPSFLLLACG----

------------------------TLVAALLGAAHRLGLFYQL-----------------

---LHKVDKASVRHGGENVAAVLRAHGVRFIFTLVGGHISPLLVACEKLG-IRVVDTRHE

VTAVFAADAMARLSGTVGVAAVTAGPGLTNTVTAVKNAQMAQSPILLLGGAASTLLQNRG

ALQAVDQLSLFRPLCKFCVSVRRVRDIVPTLRAAMAAAQSGTPGPVFVELPVDVLYPYFM

VQKEMVPAK--PPKGLVGRVVSWYLENYLANLFAGAWEPQPEGPLPLDIPQASPQQVQRC

VEILSRAKRPLMVLGSQALLT-PTSADKLRAAVETLGVPCFLGGMARGLLGRNHPLHIRE

NRSAALKKADVIVLAG-TVCDFRLSYGRVLSHSSKIIIVNRN------------REEMLL

NSDIFWKPQEAVQGDVGSFVLKLVEGLQG--QTWAPDWVEELREADRQKEQTFREKAAMP

VAQ----HLNPVQVLQLVE------ETLPDNSILVVDGGDFVGTAAHLVQPRGPLRWLDP

GAFGTLGVGAGFALGAKLCR------PDAEVWCLFGDGAFGYSLIEFDTFVRHKIPVMAL

VGNDAGWTQISREQVPSLGSNVACGLA----------------YTDYHKAAMGLGARGLL

LSR--ENEDQVVKVLHDAQ-----QQCRDGH-PVVVNILIGRTDFRDGSIAV--------

-------------------------------

>Pan_troglodytes

---------------------------------METPAAAAPARSLFPSFLLLACG----

------------------------TLVAALLGAAHRLGLFYQL-----------------

---LHKVDKASVRHGGENVAAVLRAHGVRFIFTLVGGHISPLLVACEKLG-IRVVDTRHE

VTAVFAADAMARLSGTVGVAAVTAGPGLTNTVTAVKNAQMAQSPILLLGGAASTLLQNRG

ALQAVDQLSLFRPLCKFCASVRRVRDIVPTLRAAMAAAQSGTPGPVFVELPIDVLYPYFM

VQKEMVPAK--PPKGLVGRVVSWYLENYLANLFAGAWEPQPEGPLPLDIPQASPQQVQRC

VEILSRAKRPLMVLGSQALLT-PTSADKLRAAVETLGVPCFLGGMARGLLGRNHPLHIRE

NRSAALKKADVIVLAG-TVCDFRLSYGRVLSHSSKIIIVNRN------------REEMLL

NSDIFWKPQEAVQGDVGSFVLKLVEGLQG--QTWAPDWVEELREADRQKEQTFREKAAMP

VAQ----HLNPVQVLQLVE------ETLPDNSILVVDGGDFVGTAAHLVQPRGPLRWLDP

GAFGTLGVGAGFALGAKLCR------PDAEVWCLFGDGAFGYSLIEFDTFVRHKIPVMAL

VGNDAGWTQISREQVPSLGSNVACGLA----------------YTDYHKAAMGLGARGLL

LSR--ENEDQVVKVLHDAQ-----QQCRDGH-PVVVNILIGRTDFRDGSIAV--------

-------------------------------

>Mus_musculus

---------------------------------METSAAAASAGGFFPSFLLLAFG----

------------------------TLVAAVLGVAHRLGLFYQL-----------------

---MHKVDKTSIRHGGESVAAVLRAHGVRFVFTLVGGHISPLLVACEKLG-IRVVDTRHE

VTAVFAADAVARLTGTVGVAAVTAGPGLTNTVTAVKNAQVAQSPVLLLGGAASTLLQKRG

ALQAIDQMSLFRPLCKFCASVRRVRDIVPTLRTAIAAAQSGTPGPVFVELPLDVLYPYFM

VEKEMIPTK--LPNSLMGRVVVWYLQNCLANLFVGAWEPRPEGPLPLDIPQASPQQVQRC

VEILSRAKRPLLVLGSQALLP-PTPANKLRAAVETLGVPCFLGGMSRGLLGRNHPLHIRQ

NRSAALKKADVVVLAG-AVCDFRLSYGRVLNRKSSIIIVNRN------------RDDLLL

NSDIFWKPQEAVQGDVGSFMIKLVEGLQG--QMWSSDWAEELRKADQQKEQTYRDKALMP

VLQ----HLNPVWVLQQVE------ETLPDNALLVVDGGDFVATAAYLVQPRGPLRWLDP

GAFGTLGVGAGFALGAKLCQ------PEAEVWCLFGDGAFGYSLIEFDTFVRHKVPVIAL

VGNDAGWTQISREQVPRLGSDVACSLA----------------YTDYHKAAMGLGAQGLI

LSR--DNKDQVVKVLREGQ-----QLCQDGH-AVVVNILIGRTDFRDGSISV--------

-------------------------------

>Monodelphis_domestica

---------------------------------MEASWSSAFY------YVLLACG----

------------------------TLGTISLGVAYRFGLLYQL-----------------

---LHQVDKSSSRYGGENVASVLKAHGIRFIFTLVGGHISPLLVAAEKLG-IRVVDTRQE

ATAVFAADAVARLSGTVGVAAVTAGPGLTNAVTAVKNAQMAQSPVLLLGGAASTLLQGRG

PLQGIDQMSLFRPLCKFCASVHRVRDIIPTLRAALAAAQSGTPGPVFVELPIDVLYPYFL

VQREVMPSKSKPPKSLIGKLVSWYLENHLANLFAGAWEPQPEGPLPLNIPQASSQQVQRC

VELVSRAKKPLLLLGSQALLP-PTPAESLRIAVESLGIPCFLGGMSRGLLGRNSPIHIRQ

NRRDALKEADVVILAG-AVCDFRLSYGRVLSHSSKIIAVNRD------------REQLLL

NSDLFWKPQEAVQGDVGSFLLRLSEGLRG--QTWNQEWIEQLQKADQQKEQTNREKGAEP

TAH----HLNPLWVLELVE------KTLPDNSVLVADGGDFVGSAAYLVRPRGPLRWLDP

GAFGTLGVGGGFALGAKLCR------PDAEVWVLFGDGALGYSIIEFDTFARHKVPIIAV

VGNDACWTQISREQVPILGSNVACGLA----------------YTDYHVVAEGLGSKGLL

LSR--ENEEKAEEVLHTAQ-----QLCRKGQ-PVLINVLIGKSDFRDGSISV--------

-------------------------------

>Caenorhabditis_elegans

---------------------------------MVLFLIIAAIIIGLLLWKWLDVRSTD-

----------------------ELTSMVKLLGSGNGQHVLSN------------------

---AFQVDEKSKRHGGELVASVLKAHDVEEIFVLCGGHISPILVAAEKLG-IKIVDTRHE

VTAVFAADAVARLRQSIGVAAVTAGPGLTNTITAVKNAQMAESPLLLIGGAAPTLLKGRG

ALQDIDQMVLFRPLCKYVARVERLRDIVPTVREAIKAAKSGCPGPVFVEFPVDVLYPYEL

VVKEIGFNP--NAKGFIQRALNFYLRCHVSRQFGNAWAPQTITPLPTNIPMPKSEKIQEI

VQLVKSAKRPVLLIGSQATLP-PVKPADLVKAVEALGCPVFLGGMARGLLGKDHPLQMRQ

VRRDALKDADLTILAG-TVCDFRLSYGRTLSKKSKIVALNRN------------SSQLTK

NEKAFWNSDVSVQADVATSLVQVANALGANHTTTPTEWVKSLREKDDEKESANAKKMEQK

LTNG---FLNPLNFLRTLD------QSLPDDAILVADGGDFVGSAAYIVRPRGPLQWLDP

GAFGTLGVGGGFALGAKTVY------PKRPVYIIWGDGSCGYSLMEYDTFARHKLPVIGI

VGNDACWTQIAREQVPMFQSSVAVDLA----------------RTRYDNVAKSLGSWGET

IDE--SNADSARKILDEAL-----AVCRSGEQSALVNVLIGKTDFREGSISV--------

-------------------------------

**BHMT.fasta**

>Homo_sapiens

---------MPPVGG-KKAKKGILERLNAGEIVIGDGGFVFALEKRG-----YVKAGPWT

PEAAVEH----PEAVRQ---------LHREFLRAGSNVMQTFTFYASEDKLENRGNYVLE

---------------KISGQEVNEAACDIARQVADEGD-----------ALVAGGVSQTP

SYLSCKSETE-----------------------VKKVFLQQLEVFMKK---------NVD

FLIAEYFEHVEEAVWAVETLIASG--------------------------KPVAATMCIG

PEG--DLHGVPP------------------------GECAVRLVKAG-----ASIIGVNC

HFDPTISLKTVKLMKEGLEAARLKAHLMS--------------QPLAYHTPDCNK--QGF

IDLPEFPFGLE----------PRVATRWDIQKYAREAYNLGVR---------YIGGCCGF

EPYHIRAIAEELAPERGFLPPASEKHGSWGSGLDMHTKPWVRARARKEYWENLRIASGRP

YNPSMSKPDGWGVTKGTAELMQQKEATTEQQLKELFEKQKFKSQ---

>Pan_troglodytes

---------MPPVGG-KKAKKGILERLNAGEIVIGDGGFVFALEKRG-----YVKAGPWT

PEAAVEH----PEAVRQ---------LHREFLRAGSNVMQTFTFYASEDKLENRGNYVLE

---------------KISGQKVNEAACDIARQVADEGD-----------ALVAGGVSQTP

SYLSCKSEIE-----------------------VKKVFLPQLEVFMKK---------NVD

FLIAEYFEHVEEAVWAVETLIASG--------------------------KPVAATMCIG

PEG--DLHGVPP------------------------GECAVRLVKAG-----ASIIGVNC

HFDPTISLKTVKLMKEGLEAARLKAHLMS--------------QPLAYHTPDCNK--QGF

IDLPEFPFGLE----------PRVATRWDIQKYAREAYNLGVR---------YIGGCCGF

EPYHIRAIAEELAPERGFLPPASEKHGSWGSGLDMHTKPWVRARARKEYWENLRIASGRP

YNPSMSKPDGWGVTKGTAELMQQKEATTEQQLKELFEKQKFKSQ---

>Mus_musculus

---------MAPVAG-KKAKKGILERLNAGEVVIGDGGFVFALEKRG-----YVKAGPWT

PEAAVEH----PEAVRQ---------LHREFLRAGSNVMQTFTFYASEDKLENRGNYVAE

---------------KISGQKVNEAACDIARQVADEGD-----------ALVAGGVSQTP

SYLSCKSEVE-----------------------VKKIFRQQLEVFMKK---------NVD

FLIAEYFEHVEEAVWAVEALKASG--------------------------KPVAATMCIG

PEG--DLHGVPP------------------------GECAVRLVKAG-----ASIVGVNC

HFDPSVSLQTVKLMKEGLEAARLKAYLMS--------------QPLAYHTPDCGK--QGF

IDLPEFPFGLE----------PRVATRWDIQKYAREAYNLGVR---------YIGGCCGF

EPYHIRAIAEELAPERGFLPPASEKHGSWGSGLDMHTKPWIRARARKEYWQNLRIASGRP

YNPSMSRPDAWGVTKGAAELMQQKEATTEQQLRELFEKQKFKSAQ--

>Monodelphis_domestica

---------MVPAGG-KKMKKGILQRLDSGEIVIGDGGFVFALEKRG-----YVKAGPWT

PEAAVEH----PEAVRQ---------LHREFLRAGSNVMQTFTFYASEDKLENRGNYVAE

---------------KISGQKVNEAACDIALQVAAEGD-----------ALVAGGVSQTP

SYLSCKSETE-----------------------VKKIFRQQLTVFMKK---------NVD

FLIAEYFEHVEEAVWAVEALKESG--------------------------KPVAATMCIG

PEG--DLHGVTP------------------------GECAVRLVKAG-----ASIVGVNC

HFDPTISLKTVKLMKEGLEAAKLKAYLMT--------------QPLAYHTPDCGK--QGF

IDLPEFPFGLE----------PRVATRWDIQKYAREAYKLGVR---------YIGGCCGF

EPYHIRAIAEELAPERGFLPDASEKHGSWGSGLNMHTKPWVRARARREYWESLPLASGRP

YCPSMSKPDAWGVTKGTAELMQQKEATTDQQLKELFEKQKFKSSVA-

>Xenopus_tropicalis

---------MAPTG----AKKGLLERLDAGEVVIGDGGFVFALEKRG-----YVKAGPWT

PEAAVEH----PEAVRQ---------LHREFLRAGANVMQTFTFYASDDKLENRGNYVAK

---------------KISGQKVNEAACDIAREVANEGD-----------ALVAGGVSQTP

SYLSCKSEVE-----------------------VKGIFRKQLDVFIKK---------NVD

FLIAEYFEHVEEAVWAVEVLKESG--------------------------KPVAATLCIG

PQG--DLNGVTP------------------------GECAVRLAKAG-----ASVVGVNC

HFDPMTCIATVKLMKEGLVAAKVKAHLMT--------------QPLAYHTPDCGK--QGF

IDLPEFPFALE----------PRIVTRWDIHKYAREAYNLGVR---------YIGGCCGF

EPYHTRAIAEELAPERGFLPPGSEKHGSWGSGLEMHTKPWVRARARRDYWEKLPPASGRP

CCPSMSKPDAWGVTKGDADLMQQKEATTEQQLIDLFAKQCIKSN---

>Anolis_carolinensis

---------MAPVGGTAKAKKGILERLNAGEVVIGDGGFVIALEKRG-----YVKAGPWT

PEATVEH----PEAVRQ---------LHREFLRAGANVLQTFTFYASDDKLENRGNYVAD

---------------KISGQKVNEAACNIAKEVAEEGD-----------ALVAGGVSQTP

SYLSSKSEAE-----------------------IKSIFRKQLNIFMKQ---------KVD

FLIAEYFEHVEEAVWAVETLKESG--------------------------LPIAASLCIG

PEG--DMHGISP------------------------GECAVRLVKAG-----ASIVGVNC

HFDPTTCLKTVKLMKEGLAAAKLKAHLMS--------------QPLAFHTPDCGK--QGF

IDLPEFPFALE----------PRILTRWDIHKYAREAYNLGIR---------YIGGCCGF

EPYHIRAIAEELAPERGFLPHGSEKHGSWGSDLSMHTKPWVRARARKEYWENLLPASGRP

YCPSLSKPDDWGVTKGDAELIQQKEATSEQQLKELFNKQKLKSKVVA

>Taeniopygia_guttata

---------MTGVYHINPDSNGILERLDAGEIVIGDGGFVFALEKRG-----YVKAGPWT

PEATVEH----PEAVRQ---------LHREFLRAGSSVLQTFTFYASEDKLENRGNYVAE

---------------KISCQKVNEAACDIAREVANEGD-----------ALVAGGVSQTP

SYLSCKDKTE-----------------------VKAAFQKQLEVFVKK---------NVD

FLIAEYFEHVEEAVWAVEVLKESG--------------------------KPVAATMCIG

PEG--DMHGVSP------------------------GQCAVQLVKAG-----ASIVGVNC

HFDPETSIETVRLMKEGLQAAKLKAHLMC--------------QPLAFHTPDCGK--QGF

IDLPEFPFGLE----------PRIATRWDIQKYARKAYDLGIR---------FIGGCCGF

EPYHIRAIAEELAPERGFLPEASEKHGSWGDNLSMHTKPWVRARARKEYWENLKPASGRP

YCPGMSKPDGWGVTKGAKELMQQKEATTEQQLKELFHKKKF------

>Danio_rerio

---------MAPVGS----KRGVLERLNAGEVVIGDGGFVFALEKRG-----YVKAGPWT

PEAAAEH----PEAVRQ---------LHREFLRAGSNVMQTFTFYASDDKLENRGNKLS-

----------------FTGQQINEAACDLAREVANEGD-----------ALVAGGVSQTP

SYLSCKSEEE-----------------------VKKTFKKQLDVFIKK---------NVD

LLIAEYFEHVEEAEWAVQVLKATG--------------------------KPVAATLCIG

PDG--DMPGVTP------------------------GECAVRLVKAG-----ADIVGVNC

HFDPLTCVKTVVMMKAAVEKAGLKAHYMT--------------QPLAYHTPDCSC--QGF

IDLPEFPFALE----------PRILTRWEMQQYAREAYKAGIR---------YIGGCCGF

EPYHIRAVAEELSAERGFLPEASQKHGLWGSGLEMHTKPWVRARARRDYWEKLKPASGRP

LCPSMSTPDGWGVTRGHAALMQQKEATTAEQLRPLFQQADAKH----

>Ciona_intestinalis

-------------------MKGLLERLKEG-PVVGDGSMCMTLEKRG-----YCRAGQWT

PEAVLEY----PDAVKQ---------LLREYLRAGADVLQTPCYASSDGRLK-RG---RI

---------------TYTTAEINAAACDLAYEVAQEGN-----------ALVCGGITPVL

SYLQNKGEEA-----------------------IREEFNGQLDVFIKRR-------NNVD

FILGEFFGHIEELEICVDVMKKAK--------------------------MPIACTMRIG

ALG--DLNGVSV------------------------EECAVRMAKTG-----ADLIGLNC

LFDLNATLKTLKRMKNALDAEGITTPLMC--------------QPLGFMCPEVEHTTNGY

SGLPENPLAQD----------PRQVTRFEVHKFAREAYNLGAR---------YIGGCCGM

EPHHIRAIAQELAPERNRNPPVQDMCPPCG-FLDRSCISTIKKKVRHGILDESETWKWKA

LQSSMC-----------------------------------------

>Sorghum_bicolor

-MG-----------------ALEELVAKAGGCAVIDGGFATQLEALG----ADINDPLWS

AACLIT----RPHLVKE---------VHMQYLEAGADIIISSSYQATIPGFLAR-GMSVD

---------EAEDLLRTSVKLAVEARDEFWKSALRKA------KPIYNRALVAASVGSYG

AYLADGSEYSGSYG-ADITA-----------EKLKDFHRRRLQVLASAGPD---------

LIAFEAIPNKMEAQALVELLEEEK------------------------VQVPSWICFSSV

DGK--NLCSGES------------------------FADCLKILDTS-----DKVAVVGV

NCTPPQFIEGIICEFKKQTKK------------------------AIAVYPNS---GEVW

DGRAKRWLPVE------------CLGHKSFDALAKRWQEAGAS---------LIGGCCRT

TPSTIRAVSKILKGKTGH------------------------------------------

-----------------------------------------------

>Zea_mays

-MG-----------------VLEDLVARAGGCAVIDGGFATQLEALG----ADINDPLWS

AACLIT----RPHLVKE---------VHMQYLEAGADVIISSSYQATIPGFIAR-GMSVA

---------EAEDLLRTSVKLANEARDEFWKSTLRKS------KPIYNRALVAASIGSYG

AYLADGSEYSGSYG-ADITA-----------EKLKDFHRRRLQVLASAGPD---------

LIAFEAIPNQMEAQALVELLEEEK------------------------VQIPSWICFSSV

DGK--NLCSGES------------------------FADCLKILNAS-----EKVAVVGV

NCTPPQFIEGIICEFRKQTKK------------------------AIAVYPNS---GEVW

DGRAKRWLPVE------------CLGHKSFDALAKRWQEAGAS---------LIGGCCRT

TPSTIRAVSKILKGRTGH------------------------------------------

-----------------------------------------------

>Populus_trichocarpa

-MGF-----------QKAKTSLEDLIKKAGGCAVIDGGFATQLERHG----ATINDPLWS

ALCLIK----DPDLIKR---------VHLEYLEAGADILVTSSYQATLPGFLSR-GLSAE

---------EGELLLKKSVTLAVEARNKFWDAVERNP------GHSYNRALVAASIGSYG

AYLADGSEYSGCYG-PDVNL-----------EKLKDFHRRRLQVLVKASPD---------

LLAFETIPNKLEAQACVELLEEEN------------------------INIPSWICFSCV

DGE--NAPSGES------------------------FQQCLEAINKS-----DRVKAVGI

NCAPPHFIESLICKFKELTEK------------------------LIVVYPNS---GEVW

DGRAKRWLPST------------CFDDDKFEVFATRWHDLGAS---------LIGGCCRT

TPSTIQAISKVLKDPKQS------------------------------------------

-----------------------------------------------

>Vitis_vinifera

-MG-------------KTSSLLEDLIEKAGGCAVVDGGFATQLEIHG----ATINDPLWS

ALCLIK----DPDLIKR---------VHLEYLEAGADILVTSSYQATIPGFLSK-GLSIE

---------EGELLLERSVRLAVEARDKFWDVTKRVP------GHGYNRALVAASIGSYG

AYLADGSEYSGCYG-PDMNL-----------DKLKDFHRRRLQVLVRSCPD---------

LLAFETIPNKLEAQACVELLEEEN------------------------VQIPSWICFSSV

DGE--NAPSGES------------------------FKECLDIINKS-----KKVNAVGI

NCAPPHFLESLICKFKELTEK------------------------PIVVYPNS---GEVW

DGRAKRWLPSK------------CFGDDKFELYATKWRDLGAK---------LIGGCCRT

TPSTIRAISKVLKEMS--------------------------------------------

-----------------------------------------------

>Arabidopsis_thaliana

-MVL-----------EKKSALLEDLIKKCGGCAVVDGGFATQLEIHG----AAINDPLWS

AVSLIK----NPELIKR---------VHMEYLEAGADIVVTSSYQATIPGFLSR-GLSIE

---------ESESLLQKSVELAVEARDRFWEKVSKVS------GHSYNRALVAASIGSYG

AYLADGSEYSGHYG-ENVSL-----------DKLKDFHRRRLQVLVEAGPD---------

LLAFETIPNKLEAQACVELLEEEK------------------------VQIPAWICFTSV

DGE--KAPSGES------------------------FEECLEPLNKS-----NNIYAVGI

NCAPPQFIENLIRKFAKLTKK------------------------AIVVYPNS---GEVW

DGKAKQWLPSQ------------CFGDDEFEMFATKWRDLGAK---------LIGGCCRT

TPSTINAISRDLKRR---------------------------------------------

-----------------------------------------------

>Physcomitrella_patens

-MGRTIDVPEDTLQESETSNVVLELLKQAGGCVVTDGGFATQLERHG----ANINDPLWS

AVCLIT----MPDLIRK---------VHREYLEAGAAVISTASYQATIQGFEMR-GLSTK

---------DSEDLLQLSVRIAREERDRFWKEYQNKVHTGPGQAGSYHHALVAASIGSYG

AYLADGSEYSGDYG-SFVTV-----------EKLKNFHRRRLLVLADAGPD---------

LLAFETIPCKLEIQALVELLDEEK------------------------IRIPAWVALNSK

DGV--NVVNGDS------------------------LTDCVGLLDNC-----TKVVAVGI

NCTPPRFILDLIRVARKVTSK------------------------PIMVYPNS---GEHY

DAVIKQWVECK------------GSTDTDFVSHVQEWRKAGAQ---------LIGGCCRT

TPNTIRAISRVLYEHTQVYAAK--------------------------------------

-----------------------------------------------

>Selaginella_moellendorffii

-MG-------------AGKNKLEELLESSGGCAVLDGGLATQLEHCG----ADLNDPLWS

ALCLIT----RPQLIQK---------VHWDYLEAGADILVSSSYQATVQGFVSK-GLSEK

---------EGEEMLKKSVAIACQVRDKFWDKVKQNNSSG---EIRYNRALVAASIGSYG

AYLADGSEYSGQYGPEMMNV-----------AKLKGFHRRRLQILASSGAD---------

LLAIETIPCQVEAQALVELLEEED------------------------IQIPSWISFNSK

DGA--NVVSGDP------------------------LSECVALAAKS-----AKVAAVGI

NCTPPRFIHGLVSTARKVTDK------------------------PIVVYPNS---GETF

DPDAKQWIPST------------GVSDVDFVSYVGEWKKAGAS---------LIGGCCRT

TPATIRAIKKSLQK----------------------------------------------

-----------------------------------------------

>Oryza_sativa

MVGKSGVAEEG---GAAAAAAVRRWVEAGGGRLVMDGGLATELEANG----ADLNDPLWS

AKCLLS----SPHLVRK---------VHLDYLEAGANIIITASYQATIQGFESK-GFSKE

---------QSEDLLAKSVEIAREARDMFLKEHSDR--------PIQHPILVAASIGSYG

AYLADGSEYSGDYG-EAGTL-----------EFLKDFHKRRLEVLAEAGPD---------

LIAFETIPNKLEAQAYVELLDECN------------------------ISIPAWFSFNSK

DGV--HIVSGDS------------------------LIECATIANGC-----SKVGAVGI

NCTPPRFIHGLILSIRKVTDK------------------------PILIYPNS---GERY

DAEKKEWVEST------------GVSDGDFVSYVNEWCKDGAV---------LIGGCCRT

TPNTIKAISRSLNQRHSSLHLPVA------------------------------------

-----------------------------------------------

>Pyrenophora_tritici-repentis

-MTFT-----------APPNRLSAHIVK-GSPLILDGALATYLETLG----ADISGALWS

ASILLD----QPSLIKQ---------THLDYYRANANVAITASYQASIPGLVKHLQLNEK

---------EAKDVVKKSVELAQEARDQYITESTAKVG---------NQLFIAGSVGPYG

AFLADGSEYRGDYS---IPK-----------EEMKDFHRGRIQALVEAGVD---------

ILACETIPSKAETEAIIDLLTTEF------------------------ASTEAWFGFTLR

DSE--HISDGTS------------------------LAEIAALFDNV-----QQVVALGF

NCVPDDLSVAALKTLKPLVKRG-----------------------TLVVYPNS---GEQW

NAQAREWEGKR------------TEG-SSLAEKTREWRDAGAG---------LIGGCCRT

TPKDIGVMKQALE-----------------------------------------------

-----------------------------------------------

>Ustilago_maydis_521

-------------MTASIDQGALESLLSPARIGILDGGLATYLEDGLDFDLSKG--PLWS

ARLLDE----KEDDVSDGKGQKGIFDAHLHYLQAGAGIIGTATYQASLESFARANYDQVS

---ASHLMSKAVDLACDALHAHNISNNKVGVASAASAR-----------PLLSLSLGPYG

AMLSNGAEYTGDYRRTFLAESDPLREQQPSLEEMMAFHQRRIEAFIAQP-SWEHVG----

VLAVETVPRADEALAFRMALENVARSLEQQGRPLERKPVYISMAFPDDRRLPWPPVKKSS

AAGQEGDVDMDEDNDEDEVEDEEAIQEEMNWLVQIVTDTQVQGQDSL-----WPISGIGI

NCTKPYLLPKLVERMSASLVTLNLPASEGGMDLESRRGALGLPKPLLFLYPDG---GLVY

DAVRKIWLTPSNAGDLGHGDNSAASWASNLMKLAKSITAGSDAKLPDVWRGVFVGGCCKS

GTDEIRALCQFRSS----------------------------------------------

-----------------------------------------------

>Puccinia_graminis

------------------MERLFPNHAGHPKIVLMDGGSGTTLEDEFGCRLKS---QLWS

SELLLN----RPEILSS---------LHHAWEQAGAQIISTASYQATLEGFR---SLLSQ

---SSRGETEEKDVGSDVSLQLLRRSVALARDSLSGSN-----------ARVALSLGPYG

ATLTPGQEYSGCYPAPYDSE-----------EKLVNFHFDRLMDYAEDYSTWEKVD----

IVLFETVPNLTEARAIRRAWKKFERTLHALIRRSATG------ANPDSSSKPWVISFVFP

TS--TGQFPTGENPSQ-------------VLQAALITDADAELAE---------PSGVGV

NCTKLGNLQPILEAWRTSAVDH--------------------SKTWLWLYPDG---GPTY

DSVNRSWTGSP---------ITHQEWANQLFTIASNFSAS--------WAGIVLGGCCKA

GTPHIRALHQLLSSTARNEDSLNNSD----------------------------------

-----------------------------------------------

>Gibberella_zeae_PH-1

---------------------------MPSKILILDGGLGTSLESKYSVTFSRS-TPLWS

SHLLVA----DQPTLQS--------CQSDFG-AVPVDVLLTATYQVSLHGFADTRTE---

---EFPNGISRE-----NVPRFLDDSVSIAERAVGDK------------GCVALSIGPYG

ACMIPGQEYSGKYDDKHDSL-----------QDLESWHRERLGVFSEVNDIQKRLG----

YVALETIPRLDEIIAMRKALAAT----------------------PALSKLPYWTALLSP

EKD-LRLPDGNS--------------------IEAAVEAMLDPEVSA-----NIPWGIGI

NCTKVDKLDSLLQIFESTVSNMVEKGKI-------------AEWPALVLYPDG-TNGEVY

NTTTQKWEMP-----DGAENQRRTSWEGQLEDVVKATEGRGK------WPAILVGGCCRA

GSEDIKKLRDRLV-----------------------------------------------

-----------------------------------------------

>Aspergillus_niger

--------------------------MAKTPILILDGGLGTSLQDHYNITFSSSTTPLWS

SHLMIS----DPSTLLS--------CQRDFTTTAAVDVLLTATYQVSPEGFQRTKTP---

---SHPTGIPRE-----SIAGYLRTALDVAGQAVQNTS-----------ASVALSLGPYG

ACMIPGQEYSGKYDGEHDTE-----------EKLWRWHTDRLGLFNDEAMEGMRLGERVK

YIAMETVPRIDEVRAVRRAVGSS----------------------RFCEGIPFWVACVFP

IEDKDTLPDGST--------------------VDEVVEAALLPIEGG-----ATPWGIGI

NCTKLHKLPRLVKLFGDAVERLLRDGRI-------------QERPALVLYPDG-TQGEVY

NTATQTWEKVQ----DKSGAADSRPWEVQLAQVVNDASATGQ------FSSILVGGCCKA

SFNDIKRLREQLRPQ---------------------------------------------

-----------------------------------------------

>Neurospora_crassa_OR74A

-------------------------MATPIPVQILDGGMGTTLEDMHDITFSFE-TPLWS

SHLLVSG---EEDKLSD--------CHEAFK-QAGANIISTATYQISINGFAATKAPRSG

TVDEEREGIEKE-----EIPRFLSRAVVLAANAAGTE------------GKVALSLGPYG

ATMIPSTEYSGRYDPEHQHV-----------QALGKWHKERLDLFKDVDPN--QVN----

YIAFETVPRLDEIVAIRNLLSADNIP-------------------TSLRGRPVWISSPYP

NDD-GKLPDGST--------------------VEEVVKAVLTHREGL-----ETPWGIGI

NCTKVEKLDSLVKRYEDAIQTCIKNGEQ-------------MAWPSLVLYPDG-TKGEVY

NTATKTWELS------PGHKETEAPWETVLASVVEAARQRGN------WKSIVVGGCCKA

SPEHIRRLRRTLQDYGHMSTSPAA------------------------------------

-----------------------------------------------

>Sclerotinia_sclerotiorum_1980_

-------------------------MSSECKIHLLDGGLGTTLGDSHQVQFTEK-EPLWS

SQLLIPTHPHGPNTLLA--------TQKSFV-DAGADILLTATYQASYEGFGRSGYAVHS

---HSSSGFEKEDGDKEEVNEIMRSAVDIASNAFSVKKDS--------NGKIALSLGAYG

AIMTPGQEYTGKYDDQHKSS-----------EHLSSWHHERISVFSRDPKSWDRVD----

YVAFETIPLLEEIEGVRKSMGEIERSNG-----------------GKTGSKPFWVTCVFP

GEG-NGLPDGSS--------------------VQQIVQAMLSKKEGS-----PVPFGVGL

NCTKVGKVEALILDFEREVRSLIEKGDV-------------SEWPSLVVYPDGTIKGEVY

NTSTKVWEIR------EPPGKEDLQWDEAVLEIVRRTRDRGL------WKEIIVGGCCKT

TPREIGKLRERIDRLIKE------------------------------------------

-----------------------------------------------

>Saccharomyces_cerevisiae_S288c

---------------MARLPLKQFLADNPKKVLVLDGGQGTELENRG----IKVANPVWS

TIPFISESFWSDESSAN---RKIVKEMFNDFLNAGAEILMTTTYQTSYKSVSENTPIRTL

S----------------EYNNLLNRIVDFSRNCIGEDK------------YLIGCIGPWG

AHICR--EFTGDYGAEPENID------------FYQYFKPQLENFNKNDKLD--------

LIGFETIPNIHELKAILSWDESILS-------------------------RPFYIGLSVH

EHG--VLRDGTT------------------------MEEIAQVIKDLGDKINPNFSFLGI

NCVSFNQSPDILESLHQALPN-----------------------MALLAYPNS---GEVY

DTEKKIWLPNS-------------DKLNSWDTVVKQYISSGAR---------IIGGCCRT

SPKDIQEISAAVKKYT--------------------------------------------

-----------------------------------------------

>Schizosaccharomyces_pombe

-------------------------------MLMLDGGSTAILPKLP---ESISESRLWT

SEALVRY----PEIVVK----------HHEEFLKVCDIISTFTYQLDASIYDEKVEGVPL

----------------KQVYANSIGLPVYAREHLGLPN-----------KYIALCLGSHA

ATIPGCMEYKMIYDKPTDFE------------MLYNFHKNRIEAIQASNPK---AFEKID

FIAFESLPHVTEAEVVCQLIQDMKG-----------------------WSKRCWITCTCP

ERS--TIERVSS-----------------------IISKILSINHDS-------IWGIGV

NCFHLSLLEPIAKMLSSLLPSN----------------------ITAILYPDG---RGLY

QNPDGTFSPGS--------TDHPAPSPEEWSTITAKYSNLHNG-------NLILGGCCET

NYNHLSLLREKTK-----------------------------------------------

-----------------------------------------------

>Drosophila_melanogaster

------------MTTKMAINAKDIFRNQHRLIRFLEQSIRLCSNQSLKAAQAAQLQQKSS

ADFEITASLDAQYTSAP------EPIKHDKNLGHQFRASQISVRLAAPEQLQPKPDNDEE

LG-------FGRLFTDHMLKIYYHKSLGGWQRPEITPLEN-------LVMHPAAKVLHYA

VELFEGMKAYRGVDGKIRIFRPDMN------MNRMNLAAQRSGLPTFEGKEFVQCLSRLL

SIDSEWVPHTDTASLYIRPTLIGIDPTLG------------------VASSDSALLYTIL

SPVGSYFKTGSS-------------------------GAVSLLADPSYVR--AWPGGVGN

RKMGSNYAPTINVQKEAAAKGLQQVLWLYG---EDHQLTEVGTMNIFMFFVNDQGEQELV

TPPLSGLILPG-------------ITRDSILRMTRQWGKFKVS-------EANITMPMVC

ELLNQGRLLELFGAGTACVVSPVNRISYLGQDLYIPTMEQEKPVHELIRETLTDIQYGRV

DHPWAVVID--------------------------------------

**BCA.fasta**

>Gibberella_zeae_PH-1

------------------------------------------------------------

--------------------MMRP------------------------------------

--------MLAR-----SALRSARLSSFRPLCQAQR-------FSIKAEAASSAQ-----

---------------------------------HMGLDASKLTIQKT--GNHKTLS-KPE

TLVFGKEFT----------------------DHMLAIEWN-QDKG-WLEPKITPYQNLSL

DPATCVFHYAFECFEGMKAYKDKN-GKVRLFRPDMNMARLNKSAARIALPTFEPAEFAQL

ISKLVNLDSRFIPDQRGYSLYLRPTMIGTQKTLGVGPPGSALLYCIASPVGPYYPTG-FK

AVSLEATDYAVRAWPGGVGDKKLGANYAPCILPQLQAASRGFQQNLWLFGEE-EFVTEVG

TMNMFVALKN-KETGQKELITAPL-DGTILEGVTRDSVLALARERLI--------PQGWK

ITERKYTMKELAEAADEGRLLEAFGTGTAAIVSPVRSISWKG------KLVDCGLSEVEE

SGE--------------IALQMKNWIEAIQYGD--EEH-EWSYTI---------------

----------

>Neurospora_crassa_OR74A

------------------------------------------------------------

------------------------------------------------------------

--------MFRT-----SLRRAAAFQPARPCFGAFS-------ATAARQYSAATG-----

---------------------------------TADINPSKLKVEKT--TQPKGLL-KPE

ELVFGRNFTGTVQNLPSKNAQSALTNFRATPDHMLTIEWT-KENG-WNAPEIKPYQNLSL

DPATCVFHYAFECFEGMKAYKDKA-GKIRLFRPDKNMARFNKSAARIALPTFDSPALIDL

IAKLVKLDSRFIPEQRGYSLYLRPTMIGTQKTLGVGPPGSALLYVIASPVGPYYPTG-FK

AVSLEATDYAVRAWPGGVGDKKLGANYAPCIVPQVEAMSRGFQQNLWLFGEE-EYVTEVG

TMNFFVAIKD-KKTGQKTLVTAPL-DGTILEGVTRDSVLALAREKLA--------PEGWK

IEERKFTMKELAEAANEGRLIEAFGAGTAAIVSPVRSISWKG------QLVNCGLKPEEE

SGE--------------LTMQVKNWMEARQYGD--EEH-EWSYVCE--------------

----------

>Aspergillus_niger

------------------------------------------------------------

--------------------MKSF------------------------------------

--------QFARRRALQSLVAPRAFQSP-ATAQLWK-------RCFSATSAAASQ-----

---------------------------------LAPLDPSKLTVSKT--TTPKELT-APK

DLVFGKTFT----------------------DHMLSIEWT-ATEG-WHSPQIVPYQNLSL

DPSACVFHYAFECFEGMKAYKDES-GQIRLFRPDKNMARLNKSSKRIALPSFDGEALTKL

IGEFVKLDSRFIPSERGYSLYLRPTMIGTQKTLGVGPPGSALLFVIASPVGPYYPTG-FK

AISLEATDYAVRAWPGGVGDKKLGANYAPCVLPQLEAASRGFQQNLWLFGEE-EYITEVG

TMNLFVALKN-KETGQKELITAPL-DGTILEGVTRDSVLGLARERLV--------PKGWN

ISERKLKMSEVAEAAEEGRMIEVFGAGTAAIVSPVRTISYRG------KLVDCGLKETEE

AGE--------------IALQMKNWIEGIQYGD--ESH-PWSYVL---------------

----------

>Sclerotinia_sclerotiorum_1980_

------------------------------------------------------------

--------------------MLRQ------------------------------------

--------LFPRG-AAFAKPSSRVLRR----WEASS-----RLYSIQAEAASTIK-----

---------------------------------LQDIDPTKLTITKN--TTPSELL-PPN

ELVFGRSFT----------------------DHMLSIEWT-ASEG-WLPARITPYQNLSL

DPASCVFHYAFECFEGMKAYKDKS-GNVRLFRPTKNMERMNKSSARIALPTFDQSALIEL

ISKFVAMEKRFIPDARGYSLYLRPTMIGTQRTLGVGPPGSALLYVIASPVGPYYPTG-FK

AISLEATDYAVRAWPGGVGDKKLGANYAPCILPQLEAAKRGFHQNLWLFGEE-EFVTEVG

TMNMFVAVKN-KE-GQKELVTAPL-DGTILEGVTRDSVLSLAREKLT--------KEGWI

ISERKYTMKELSDASKEGRLLEAFGAGTAAVVSPVRNISWKG------NLVECGLRPDQE

AGE--------------IALKMKEWIEARQYGD--EEH-EWSYVVPN-------------

----------

>Pyrenophora_tritici-repentis

------------------------------------------------------------

--------------------MIQQ------------------------------------

--------RSSR---LFSSLSHCAFRSSVAPLSASQRCRIRRPYSIHADVSVGEK-----

---------------------------------IQNIDPSKLSITRT--TTPKDLL-PPE

ELIFGRNFS----------------------DHMLSLEWT-ATQG-WLPARITPYQNLSL

DPATCVFHYAFECFEGMKAYKDKD-GNIRLFRPDKNMARLNKSSARIALPTFDPDALIEL

IGKFVKEDERFVPDARGYSLYLRPTMIGTQRTLGVGPPASALLYVIASPVGPYYPTG-FK

AISLEATDYAVRAWPGGVGDKKLGANYAPCIVPQMQAASRGFHQNLWLFGEE-EYITEVG

TMNLFAAIKN-KETGKPELLTAPL-DGTILEGVTRDSILQLARERLE--------PKGWT

VSERKFTMKEVADAADEGRMMEIFGAGTAAIVAPVRKISWKG------RLVDCGLEDHVE

AGP--------------IAQQMKEWMEGIQYGD--EDH-PWR------------------

----------

>Saccharomyces_cerevisiae_S288c

------------------------------------------------------------

------------------------------------------------------------

-----------------------------------------------------MT-----

---------------------------------LAPLDASKVKITTT--QHASKPK-PNS

ELVFGKSFT----------------------DHMLTAEWT-AEKG-WGTPEIKPYQNLSL

DPSAVVFHYAFELFEGMKAYRTVD-NKITMFRPDMNMKRMNKSAQRICLPTFDPEELITL

IGKLIQQDKCLVPEGKGYSLYIRPTLIGTTAGLGVSTPDRALLYVICCPVGPYYKTG-FK

AVRLEATDYATRAWPGGCGDKKLGANYAPCVLPQLQAASRGYQQNLWLFGPN-NNITEVG

TMNAFFVFKD-SKTGKKELVTAPL-DGTILEGVTRDSILNLAKERLE--------PSEWT

ISERYFTIGEVTERSKNGELLEAFGSGTAAIVSPIKEIGWKG------EQINIPLLPGEQ

TGP--------------LAKEVAQWINGIQYGE--TEHGNWSRVVTDLN-----------

----------

>Schizosaccharomyces_pombe

------------------------------------------------------------

---------------MSLMFLRRA------------------------------------

--------GNIKGRNIRFALQRGSVGYSQQSSEACKNFLNTTQLRTMVQTAALHG-----

---------------------------------PKPMDSSHIKVTNV--KELKPLP-EWK

SLKFGENFT----------------------DHMLIMKWN-REKG-WSTPEIVPFGKLCF

HPASSVFHYGFECFEGMKAFRDEK-GVPRLFRPIKNAERMLSTGTRISLPSFDPAELAEI

IRKFVAHENRWVPDQRGYSLYIRPTFIGTDEALGVHHCDNAMLYVIASPVGPYYSSG-FK

AVKLCCSEESVRAWPGGTGHYKLGGNYAPSVLPQKEAAKKGYAQILWLYGDE-DYITEVG

TMNCFTVWIN-KN-GEKEIITAPL-DGMILPGVTRDSILEICRERLA--------PKGWK

ITEGKYSMKEVAQASKEGRLLEVFGAGTAALVSPVKAINYKG------TEYEIPMPEGQE

AGP--------------ITSEISKWILDIQYGK--EPNNPWSVPALP-------------

----------

>Ustilago_maydis_521

MTRRQKCGPERMVRMGRDPRRGQAATASRKQATATACAESSLTEKGESAVIRLTNVDEAL

WVRHLVVIGSDGSSDVLISNVTRAGSASSVVDDVDERGQRSGPWIAMDVKCRCPNANAFL

RLGRKKVDCLSKYPKNRWQIAVAANRPSHETVTSHESRVHRAAMLNFAKTALRARAVPA-

--TSLLRRNAAGSSFRFQSTTVSHDPMTGEPTGLKHLDASALVVTKS--NAPRVPP-PSQ

SLVFGANFS----------------------DHMLSVPWN-SATG-WDAPKIHPYAPLQL

DPSAVIFHYAPSLFEGMKAYKDVN-GKVRLFRPDMNMKRMNTSAARIALPTFEGEQLITL

IKKLVALDKSWIPSEPGHSLYIRPALIGTEAALGVHPTKDALLFVICSPVGPYYKTG-FK

PVALEADPNKVRAWPGGTGQYKLGGNYAPGILPQLEAAERGYQQNLWLFGDQ-HLLTEVG

TMNLFVALKK-KDGKGIELVTPPL-NGMILPGVTRDSILHIAKDHVNGKYALESLPKDLE

VNEREISIHEVIEAEKSGALVEMFGAGTAAVVSPVDRVGYKG------KDIHIPAG--DG

IGP--------------IAKAMLERITDIQLGK--VEH-EWSVVVDDL------------

----------

>Puccinia_graminis

------------------------------------------------------------

----------------MFSPAARP-------------------------ICRLSHS----

-------LCMRK-PSGSLDITHFSSTIHHPFSTCHR---------NLSQSAN--------

---------TSG------------------------IEASNLVIKTT--EAPRTKL-PAS

ELKFGQTFT----------------------DHMLIVKWT-RQDG-WKAPEIKPYGNLEI

DPSASVLQYATCLFEGMKAYKSND-GKIRLFRPEMNMKRMNQSARRLAFPSFEGEQLLEL

IKKLVKLEGEWIPTEAGHSLYIRPTIIGTGAGLGVGPPTELTLFVICSPVGPYYRTG-FK

PVSLLASSKYCRAWPGGSGAFKLGANYPTGFLPQMEANSDGFEQILWLFGEN-DNLTEVG

TMNLFVALED-KSGT-IELVTPPL-DDKILPGVTRCSVLELLRSHLDGSQRLDGLPPKFK

LSERPITMIEMLEKSKTGHLKEVFGTGTAAIVSSVERIGYKG------KDIHVPVGP-GG

LGV--------------FANVVQREILGRQTGE--IPS-DWAVVV---------------

----------

>Homo_sapiens

------------------------------------------------------------

------------------------------------------------------------

------------------------------------------------------------

------------MKDCSNGCSAECTGEGGSKEVVGTFKAKDLIVTPA--TILKEKP-DPN

NLVFGTVFT----------------------DHMLTVEWS-SEFG-WEKPHIKPLQNLSL

HPGSSALHYAVELFEGLKAFRGVD-NKIRLFQPNLNMDRMYRSAVRATLPVFDKEELLEC

IQQLVKLDQEWVPYSTSASLYIRPTFIGTEPSLGVKKPTKALLFVLLSPVGPYFSSGTFN

PVSLWANPKYVRAWKGGTGDCKMGGNYGSSLFAQCEAVDNGCQQVLWLYGED-HQITEVG

TMNLFLYWIN--EDGEEELATPPL-DGIILPGVTRRCILDLAHQWGE-----------FK

VSERYLTMDDLTTALEGNRVREMFGSGTACVVCPVSDILYKG------ETIHIPTMENGP

K----------------LASRILSKLTDIQYGR--EESD-WTIVLS--------------

----------

>Pan_troglodytes

------------------------------------------------------------

------------------------------------------------------------

------------------------------------------MASPLRSAAAL-------

-----------ARQDCSNGCSAECTGEGGSKEVVGTFKAKDLIVTPA--TILKEKP-DPN

NLVFGTVFT----------------------DHMLTVEWS-LEFG-WEKPHIKPLQNLSL

HPGSSALHYAVELFEGLKAFRGVD-NKIRLFQPNLNMDRMYRSAVRATLPVFDKEELLEC

IQQLVKLDQEWVPYSTSASLYIRPTFIGTEPSLGVKKPTKALLFVLLSPVGPYFSSGTFN

PVSLWANPKYVRAWKGGTGDCKMGGNYGSSLFAQCEAVDNGCQQVLWLYGED-HQITEVG

TMNLFLYWIN--EDGEEELATPPL-DGIILPGVTRRCILDLAHQWGE-----------FK

VSERYLTMDDLTTALEGNRVREMFGSGTACVVCPVSDILYKG------ETIHIPTMENGP

K----------------LASRILSKLTDIQYGR--EESD-WTIVLS--------------

----------

>Mus_musculus

------------------------------------------------------------

---------------------------------------------MLHRLRSPLRRVLAG

GSPIVPLSPATPN--CSPGLSGSAVRHESPLCHVFPRSRSFVMACLPRATATL-------

-----------ARQDCSNGCSAPFAGERGSEEVAETFRAKDLIITPA--TVLKEKP-DPD

SLVFGATFT----------------------DHMLTVEWS-SASG-WEKPHIKPFGNLPI

HPAASVLHYAVELFEGLKAFRGVD-NKIRLFRPDLNMDRMCRSAVRTTLPMFDKEELLKC

ILQLLQIDQEWVPYSTSASLYIRPTFIGTEPSLGVKKPSKALLFVILSPVGPYFSSGSFT

PVSLWANPKYIRAWKGGTGDCKMGGNYGASLLAQCEAVENGCQQVLWLYGKD-NQITEVG

TMNLFLYWIN--EDGEEELATPPL-DGIILPGVTRQSILELAQQWGE-----------FK

VCERHLTMDDLATALEGNRVKEMFGSGTACVVCPVSDILYKG------QMLHIPTMENGP

K----------------LASRILGKLTDIQYGR--VESD-WTIELP--------------

----------

>Monodelphis_domestica

------------------------------------------------------------

------MRQLLVDFPDGVSRQFGILYWGSALKEREGRALFVSCECEEGTYALTLSRLLIP

GPVELELEHHAASGPSAAGTAGSFLGRSLPLLLLLWRSLVLKQLLPPLRCTAITLLDTAW

PSSLEQPRPFPARYECNSECSIGCSGTGEPKQMAGTFKAKDLIITPA--TILKEKP-DPN

NLVFGTVFT----------------------DHMLTVEWS-LNFG-WEKPQIKPLENLSL

HPGSSALHYAVELFEGMKAYRGVD-EKIRLFRPNLNMDRMLRSAIRVTLPEFDKEELLKC

IQELVKLEQEWVPHSTSASLYVRPTLIGTEPSLGVKKPTKALLFVILCPVGPYFASGSFN

PVSLWANPKYVRAWKGGTGDYKIGGNYGASIFAQNEALHYGCQQVLWLYGED-HQITEVG

TMNLFLYWIN--EDGEDELATPPL-DGIILPGVTRQSILDLAHKWDE-----------FK

VTERYLTMNDLIVALKENRVKEMFGCGTACVVCPVSDVLYKG------ENIHVPTMENGP

K----------------LATRILNQLTDIQIPV--ADTS-RIKLVSSGSSCVGCSLGSRD

SRLQVAGTDC

>Anolis_carolinensis

------------------------------------------------------------

------------------------------------------------------------

-------------MAFSRYNRMAHVRKEDLWKNIFGVALIPIQIEVKDYSCEN-------

----------VTLHGCQNDCAAG-CMGGAAKHVTESFKAADLIINPS--TTLKEKP-DPN

GLVFGTVFT----------------------DNMLFVEWS-LTSG-WEAPHIKPLENLSL

HPAVSAFHYAVELFEGMKAYRGVD-GKIRFFRPRLNMDRMLRSAVRATLPGFDKEELLEC

IRKLVEVEKEWVPYSTSASLYIRPTLIGTEPSLGVKKPSKAILYVILSPVGPYFSSGTFS

PISLWADPKYVRAWKGGTGDCKLGGNYGSSIYAQREAMELGCQQVLWLYGDE-HQITEVG

TMNLFLYWRN--EDGEEELATPPL-DGIILPGVTRQSILDLARKWEE-----------FK

VSERYITMSDLQAALKENRVKEMFGAGTACVVCPISTILYLG------ENLHIPTMENGP

Q----------------IATRFLNQLSDIQYGR--EDSE-WSVVVS--------------

----------

>Taeniopygia_guttata

------------------------------------------------------------

------------------------------------------------------------

------------------------------------------------------------

-----------MIKGCQSECTGGGYSGETGKQMTESFKASDLIITPA--TTFKEKP-DPT

GLVFGTVFT----------------------DNMLTIEWS-LASG-WEKPYIKPLENLSL

HPASSALHYAIELFEGMKAYRGVD-GKIRLFRPALNMDRMARSARRTTLPSFDQNELLEC

IRKLVEVEQEWVPYSTSASLYIRPTLVGTEPSLGVKKPTKALLYVILSPVGPYFASGSFN

PISLWADPKYVRAWKGGTGDCKVGGNYGSAIYAQQEALEFGCQQVLWLYGED-HQITEVG

TMNLFLYWIN--EDGENELATPPL-DGIILPGVTRQSILDLARNWGE-----------FK

VSERYITMSDLTVALEESRVKEMFGAGTACIVCPISKILYKG------KHLHIPTMENGP

E----------------VTTRFLNKLSDIQYGR--EDSD-WAVLVS--------------

----------

>Xenopus_tropicalis

------------------------------------------------------------

------------------------------------------------------------

----------------------------------------MANRAAPGIWHSN-------

-----------SKKGTQNGNSCTNGDPGAQ---SDSFKASDVIINLA--SSSKEKP-DPN

SLVFGATFT----------------------DHMLCIDWS-KETG-WQRPVIKPFQNLSL

HPAVSALHYAIQLFEGLKAYRGED-GKIRLFRPKLNMERMHRSALRMTLPGFDKEELLQL

ILRLVEVDREWVPYSSSASLYIRPTFIGTEPTLGVRKPSHALLYVILSPVGPYFSSGSFN

PVSLWAEPKYVRAWMGGTGDCKVGGNYGPTIFAQYEAMEVGCQQVLWLYGEQ-EQITEVG

TMNLFIYWKD--ENGEEELVTPPL-DGIILPGVTRHSILDLARKWGE-----------FK

VSERHLTMKDLVAALNENRIREMFGAGTACVVCPVSRVSYRN------KDLHVPTMENGP

K----------------MAKRFLQELTDIQYGR--SPSD-WMVEVPSP------------

----------

>Danio_rerio

------------------------------------------------------------

------------------------------------------------------------

------------------------MNPDYFFFHMHVQSVTMNKQLSTCPAVLP-------

------------TPLCLFVFSPFFCLFLSLCLSDCLCQAADLTIEKS--SVLKPKP-DPS

TLVFGKQFS----------------------DHMLTISWS-AAGG-WENPQIKPFQNLSL

HPACSALHYSIELFEGMKAFRGVD-NNIRLFRPMLNMERMHRSAERSCLPLFDKAELLKC

INKLVETDQEWVPYSTDASLYIRPTFIGTEPSLGVSRAGHALLFVIVGPVGPYFATGSFN

PVSLLADPRYVRAWRGGVGEYKMGGNYGPTIAVQNEAAKQGCQQVLWLYGES-EEITEVG

TMNLFIYWTT--KKGEKELVTPPL-DGVILPGVTRQSLLDLAREWGE-----------FK

VTERRVFMKELLGALDEGRVLEVFGSGTACVVCPVGSLLYKG------QTYQIPTMKNGP

D----------------LAKRFHKELTDIQYGR--LQRD-WAPLVV--------------

----------

>Ciona_intestinalis

------------------------------------------------------------

------------------------------------------------------------

------------------------------------------------------------

------------------------------MTLSIRFQASDLRLNLT--RHPGPKPEDPN

KCIFGKHFT----------------------DHMLAVEWN-LKTG-WGVPVIKPLENISL

HPATSVFHYATELFEGMKAYRTVD-NRIAMFRPMENMNRMNRTAKRACLPEFDNEELVKC

LAELVRVEHEWVPHSTTASLYMRPTMIGTEPSLGVNMSSSALLYCILCPVGPYFDTGSFS

PVSLMADPTYIRAAKGGSGAYKLGSNYGPTIRIQQEAIKRGCQQVLWLYGED-HQVTEVG

TMNVFVYWIN--EDGEKELATMPLGEGIVLPGVTRSSLIELAHEWDE-----------FK

VTERIITMADVSKALNENRMLEMFGAGTACVVCPIDKILYMD------QTLEIPTMENGP

E----------------IASRFYKELTDIQYGR--IPHE-WSYTVMEDAGKGDVSEARNN

F---------

>Drosophila_melanogaster

------------------------------------------------------------

------------------------------------------------------------

------------MTTKMAINAKDIFRNQHRLIRFLEQSIRLCSNQSLKAAQAAQLQQKS-

------SADFEITASLDAQYTSAPEPIKHDKNLGHQFRASQISVRLAAPEQLQPKPDNDE

ELGFGRLFT----------------------DHMLKIYYH-KSLGGWQRPEITPLENLVM

HPAAKVLHYAVELFEGMKAYRGVD-GKIRIFRPDMNMNRMNLAAQRSGLPTFEGKEFVQC

LSRLLSIDSEWVPHTDTASLYIRPTLIGIDPTLGVASSDSALLYTILSPVGSYFKTGSSG

AVSLLADPSYVRAWPGGVGNRKMGSNYAPTINVQKEAAAKGLQQVLWLYGED-HQLTEVG

TMNIFMFFVN--DQGEQELVTPPLS-GLILPGITRDSILRMTRQWGK-----------FK

VSEANITMPMVCELLNQGRLLELFGAGTACVVSPVNRISYLG------QDLYIPTMEQEK

P----------------VHELIRETLTDIQYGR--VDHP-WAVVID--------------

----------

>Caenorhabditis_elegans

------------------------------------------------------------

------------------------------------------------------------

------------MPAILSRVAPRTFN--------------LVG--SRLMASAARLE----

--------------------TVPREEIHKEYDRKKTFYHRDLEIQLAGPTQLKTKPLDPT

KLKFGHTYA----------------------DYMMTCDWD-AERG-WHHPKIEPIGELKI

HPGAKVLHYASELFEGMKAYRGID-NKIRMFRPEMNMARMKRTALRAALPDFDSEEMINV

LTELLRLDQEWVPNSDVCSLYLRPTLIGTDPTLGVGCATEAKMFVITGPVGAYYSTG-FQ

PVSLLADSRFIRAFPGGVGAYKMGCNYAPTIWVGKEAASKNCQQVLWLYGEN-EDLTEVG

TMNIFLFWKN--EEGDMELITPPLHRGLILPGVTRDSLLELGREWGE-----------YK

VTERTLNMEEVKKALSEKRLYEMFGSGTACVVSPVGKILYHNKVTDEYEELHIPTMSSKF

G----------------VMQKFYNTINDIQYGR--IIKDGWMRDI---------------

----------

>Physcomitrella_patens

------------------------------------------------------------

------------------------------------------------------------

------------------------------------------------------------

--------------------------------------------------QVKPKL-DAT

TLKFGAVYS----------------------DHMLQASWK-DGLG-WTAPSIDPLGPISL

HPCAQVLHYAVECFEGMKCYKGKD-GRLRLFRPDMNMDRLARSAARLTLPHFDKVELLEC

IKELLRVERDWVPDKEGFSVYIRPTMIGTHPFLGVGPTREALLFVVLSPVGPYFPSG-LK

PIKLYVETQNVRAFPGGVGDKKIGGNYAPTILPQLNGSHLGCSQVCFHYSHS-VNL-KSG

SMNMFFLIRQEGKDGDLELVTPPL-DGLILPGVTRDTVLTLARGFGG-----------MK

VSERPLRFDELERAAETGRLLEVFGTGTACMIQPVMGLVKKDH-----TEIAIPFNEEAA

N----------------LCGRLSRALLDVQYGY--IDSQ-WSIMVDP-------------

----------

>Selaginella_moellendorffii

------------------------------------------------------------

------------------------------------------------------------

------------------------------------------------------------

---------------------------------------------------VKPVA-DAN

VLKFGAETT----------------------DHMLQISWN-EQQG-WLSPEINPLQHLKL

HPCAQVLHYATECFEGMKAYKDKD-GRIRLFRPLMHMNRLLKSAQRLALPEFDKMELLDC

IKELVRLERDWIPSKEGFSLYIRPAMIATQACLGIQAPREGMLFVVLSPVGPYFPTG-LK

PVKLFVDRRNVRAYPGGVGDRKLGGNYAQTVVPQLEAAKRGCSQVLYVLEGT-ERIGEAG

SMNIFFLLRDGTKSGAMELVTPAL-DGTILPGVTRDTVLKLASNIPG-----------LK

VSERPLNFPEIEAAVARGDLLEAFGTGTACMIQPVEGMLKENG-----QLISVPFNLEAA

NQWISRHDCKSREFPFNLCGYLTRTISDIHYGH--VPSE-WSVVVN--------------

----------

>Sorghum_bicolor

------------------------------------------------------------

---------------------------------------------------------MAT

RRRLDGVGQFGQSRDPNLQAAAAATRVYFLVRSSPPNLPVNCKPTYPRSPVTGLRPDYK-

------------------WSSAPARPPVLRPSLPAVARCHPRASFPVNTGTPDLADFNWD

ALGFQLIP----------------------TDFMYLMSCS--SEGVFTNGKLVPYGPIEL

NPAAAVLNYGQGLLEGLRAHRKED-GSILLFRPHENALRMKTGADRLCMPAPSVEQFLEA

VKLTVLANKHWVPPFGKGSLYIRPQLIGSGAILGVAPAPQYTFIVFVCPVGHYFKDG-LS

PISLLTEEEYHRAAPGGTGDIKTIGNYASVVSAQRRAKEKGHSDVLYLDPIHNKFVEEVS

SCNIFMVKDN--------VISTPLLTGTILPGITRRSVIEISQNLGF------------Q

VEERPITIDELLGAD------EVFCTGTAVVVSPVGSITYRG------RRVEYGKNQEAG

A----------------VSQQLYAAFTAIQKGV-TEDRMGWTLQLA--------------

----------

>Oryza_sativa

------------------------------------------------------------

---------------------------------------------------------MEL

LPRVG-VAAPGPGRG---GASPSPTR-----RHRAPSHPILKRSAAVCGAVAVCR-----

-------------------GGAVARR----------SRWSTLVTAAYYTGTAELVDFNWE

TLGFQPVP----------------------TDFMYVMRCS--EEGVFTKGELVPYGPIEL

NPAAGVLNYGQGLLEGLRAHRKED-GSVLLFRPDENALRMRVGADRLCMPAPSVEQFLEA

IKLTILANKRWVPPTGKGSLYIRPLLIGSGAILGVAPAPEYTFVVFACPVGHYFKDG-LS

PISLLTEEEYQCAAPGGTGDIKTIGNYASAVYAKERAKERGHSDVLYLDPVHKKFVEELS

SCNIFMVKDN--------IISTPLLTGTVLPGITRRSIIEYARSLGF------------Q

VEECLITIDELLDAD------EVFCTGTSVVLSSVGCIVYQG------RRVEYG-NQKFR

T----------------VSQQLYSALTAIQKGL-VEDSMGWTVQLN--------------

----------

>Populus_trichocarpa

------------------------------------------------------------

------------------------------------------------------------

MERSAVFGGLQPNYLLYPSPNSS---------SLPFSDHRARLPNFSPPPSLSLK-----

--------------------IHKQVSSCFKA---VSPFKRGAAFSDTHSDTFELADIDWD

DLGFAYVP----------------------TDYMYSMKCT--KGGNFSKGELQRYGNIEL

NPSAGVLNYGQGLFEGLKAYRKED-GNLLLFRPEENAMRMIMGAERMCMPSPTIDQFVDA

VKATVLANKRWVPPPGKGSLYIRPLLMGSGAVLGLAPAPEYTFLIYVSPVGNYFKEG-VA

PIHLIVEHELHRATPGGTGGVKTIGNYA---AAQSAAKARGFSDVLYLDCVHKKYLEEVS

SCNIFVVKGN--------SISTPAIKGTILPGITRKSIIDVARSQGF------------Q

VEERLVTVDELLDAD------EVFCTGTAVVVSPVGSITYKG------KRVSYG-VEGFG

A----------------VSQQLYSVLTKLQMGL-IEDKMNWTVELS--------------

----------

>Vitis_vinifera

------------------------------------------------------------

-----------------------------------------------------------M

ISRRACFRNLIQS--LRTGSTAS---------KLRSSN------CYTSRTAPSLQ-----

--------------------PVVELS------------------PDSDD---EYADVDWD

NLGFSLRP----------------------TDYMYITKCS--GGDSFEEGHLSRYGNIEL

SPSAGVLNYGQGLFEGTKAYRREN-GRLCLFRPDQNAIRMQAGAERMCMPSPSIHQFVEA

VKQTAFANKRWIPPPGKGSLYIRPLLMGSGPVLGLGPAPECTFLIYASPVGNYFKEG-LA

PLNLFIDDEYHRATRGGAGGVKTITNYSPVLKALTRAKSRGFSDVLYLDSVYKKNIEEVS

SCNVFIVKDN--------VISTPATGGTILEGVTRKSIVDIALDHGY------------Q

VKEQCIPVEEVMEAD------EVFCTGTAVGVAAVGSITYQG------KRVEY--KMGDQ

C----------------VSQELFSTYMGIQKGD-IEDKKGWILEID--------------

----------

>Zea_mays

------------------------------------------------------------

-----------------------------------------------------------M

AALTSAKGALLPSWARSSSSGHGGDLWRVLGKALATAGGGGGGGCSLLLPRRWQS-----

--------------------SLPQLD----------------HVADRSNEE-SGGEIDWD

NLGFGLTP----------------------TDYMYVTRCSPEDRGDFPRGELCRYGNIEL

SPSSGVLNYAQGLFEGMKAYRRPDRAGYTLFRPEENARRMQRGAERMCMPAPSVEQFVHA

VRQTVLANRRWVPPQGKGALYLRPLLVGSGPILGLAPAPEYTFLIYAAPVGNYFKEG-LA

PINLVVHDEFHRAMPGGTGGVKTIANYAPVLRAQMDAKSKGFTDVLYLDSVHKRYLEEVS

SCNVFVVKGG--------VVATPDTRGTILPGITRKSVIELARDRGY------------K

VEERLVSIDDLVAAD------EVFCTGTAVVVAPVSTVTYQG------ERYEF--RTGPD

T----------------VSQELYTTLTSIQMGMAAEDSKGWTVAVE--------------

----------

>Arabidopsis_thaliana

------------------------------------------------------------

------------------------------------------------------------

-MAPSVHPS----------------------------------------SSPLFT-----

--------------------SKADEK---------------------------YANVKWD

ELGFALVP----------------------TDYMYVAKCK--QGESFSTGEIVPYGDISI

SPCAGILNYGQGLFEGLKAYRTED-GRITLFRPDQNAIRMQTGADRLCMTPPSPEQFVEA

VKQTVLANNKWVPPPGKGALYIRPLLIGTGAVLGVASAPEYTFLIYTSPVGNYHKAS-SG

-LNLKVDHNHRRAHFGGTGGVKSCTNYSPVVKSLIEAKSSGFSDVLFLDAATGKNIEEVS

TCNIFILKGN--------IVSTPPTSGTILPGITRKSICELARDIGY------------E

VQERDLSVDELLEAE------EVFCTGTAVVIKAVETVTFHD------KRVKY--RTGEE

A----------------FSTKLHLILTNIQMGV-VEDKKGWMMEIDHLVGTDSFPDET--

----------

>Chlamydomonas_reinhardtii

------------------------------------------------------------

-----------------------------------------------------------M

SLGFRLWQNLLPTMNRSLAPSKA--------QCLRRCGAANIARTRLHTIAAAAAP----

--------------------VAGERR--------------------------AKANIDWD

SLGFGLKDV---------------------AATMYVAEWT--PEKGWDSGKLVPYGPLNM

MPSAQVLNYGQAIFEGMKAQESAK-GRVVLFRPDQNAARFKAGAARMSMPSVPEDQFVEA

VRATVENNLDYVPPMGKGSLYLRPLLMGTGPILGLGPAPAYTFAIFAAAVGAYFKGGQLT

PIDLIVEERFHRAAPGGMGGTKAAGNYSPVLVTQLEAKKNGFADVVYLDAKTDTYLEEVS

SCNIFVVKGK--------TIKTPPLSGTILPGVTRRSIIELARSRGY------------D

VQEAAVSVHEAMEDR------PARWLWRCTRPSPTS----RP------RRPTTS-SPGST

P----------------SSKCMHACLLAA-------------------------------

----------

**SD.fasta**

>Anolis_carolinensis

KKKKILILGSGYISGPVIEYLTRDPN--------------------IEITAVSVMKKQLE

QLTKKYTNVAPVVADVTEDETKLSSMVKKHNLVISLLPYVYHPLVAKKCIENKVNMVTAS

YLTPAMKELQESVEAAGITVVSEMGLDPGLDHMLAMDCIDKAKEVGATVVSYTSFCGGLP

APEYSDNPLRYKFSWSPQGVLLNTVQPATYLKNGEIINIPAGGALLDS-VTVMDFFPGLN

LEGFPNRDSTKYAEPYGIQ-SAHTLLRGTLRYRGYSKAISGFVKLGLIDSEPCPMLSATA

----PSINWKDLMCKLLGLH------------------PSASLEDLRDAVYHKLNKDEKQ

LEAVEW-----LGLLRDEP--VPI----------AETIVGALAKHMEAKLSYGAGERDMI

VMRNEIGIKHPSG-HLEDKYVNLVVYGD----------KGYSAMAKTVGYPTAIAAKMVL

DG--EINAKGMVIPLTKNIYGPILKRVQAEGISYSTQSVIKQ-

>Taeniopygia_guttata

DEKRVLLLGSGYVSGPVLEYLTRDSS--------------------VDITVVSVMKDQLE

QLTKEYRNVTPVHMDVLKHEEKLSSLVKKHDLVISLLPYSAHPFVAKKCIDNKVNLVTAS

YLTPAMKELQESVEAAGITVVSEMGLDPGLDHMLAMECIDKAKEVGASVVSYTSFCGGLP

APEHSDNPLRYKFSWSPQGVLLNTVQSATYLKDGEIINIPPGGALLDS-VTPMDFFPGLN

LEGFPNRDSTKYAEPYGIQ-TAHTLLRGTLRYKGYSRTMGGFVKLGLINPDPYPLLSSTT

----PPLTWKELMCKLVGIK------------------SPAEYHVLKEAVFSKLEKDESQ

LEAVEW-----LGLLGDEP--VPA----------ADSIVGALAKHMEMKLPFGIGERDMI

VMRSEIGLRHPSG-HLEDKFIDLVVYGDN---------KGYSAMAKTVGYPTAIAAKMVL

DG--EITAKGMVIPLTKNVYGPILERVQAEGIMYSTRSIIKQ-

>Xenopus_tropicalis

TKKKILVLGSGYVSEPVINYLTRDPN--------------------VEITAVSMVKDQVD

HLSKRYHNTTPIAMDIFKNEEKLSALIKKHDLVVSLLPYSAHPSVAKKCIKNKVNLVTAS

YISPAMKELQQGAEDAGIIIVGEMGLDPGLDHMLAMECFDKAKDVGAKVESYISFCGGLP

APEFSDNPLRYKFSWSPLAVLFNTVQPATYLKDGQIVNIAAGGSLLES-VTAMDCFPGLN

LEGFPNRDSTKYAEPYGIQ-TAHTLMRGTLRYKGFCNAMSGFVQLGLINSDPCPLLGMNA

----PAITWRELLCHLMNVS------------------SSTSIGLLKELVYNKLDKNDSN

METLEW-----FGLLSEEP--VPV----------ADSIVGALAKHLEMMLSFGPGERDMI

VLRNDIGIRHPSG-HLECKNISLVVYGDV---------NGYSAMAKTVGYPTAIAAKMVL

DG--EVESRGLVIPLTKNIYGPILERVKEEGILYSTKSTFSL-

>Homo_sapiens

TRRKVLVLGSGYISEPVLEYLSRDGN--------------------IEITVGSDMKNQIE

QLGKKY-NINPVSMDICKQEEKLGFLVAKQDLVISLLPYVLHPLVAKACITNKVNMVTAS

YITPALKELEKSVEDAGITIIGELGLDPGLDHMLAMETIDKAKEVGATIESYISYCGGLP

APEHSNNPLRYKFSWSPVGVLMNVMQSATYLLDGKVVNVAGGISFLDA-VTSMDFFPGLN

LEGYPNRDSTKYAEIYGIS-SAHTLLRGTLRYKGYMKALNGFVKLGLINREALPAFRPEA

----NPLTWKQLLCDLVGIS------------------PSSEHDVLKEAVLKKLGGDNTQ

LEAAEW-----LGLLGDEQ--VPQ----------AESILDALSKHLVMKLSYGPEEKDMI

VMRDSFGIRHPSG-HLEHKTIDLVAYGDI---------NGFSAMAKTVGLPTAMAAKMLL

DG--EIGAKGLMGPFSKEIYGPILERIKAEGIIYTTQSTIKP-

>Pan_troglodytes

TRRKVLVLGSGYVSEPVLEYLSRDGN--------------------IEITVGSDMKNQIE

QLGKKY-NINPVSMDICKQEEKLGFLVAKQDLVISLLPYVLHPLVAKACITNKVNMVTAS

YITPALKELEKSVEDAGITIIGELGLDPGLDHMLAMETIDKAKEVGATIESYISYCGGLP

APEHSNNPLRYKFSWSPVGVLMNVMQSATYLLDGKVVNIAGGISFLDA-VTSMDFFPGLN

LEGYPNRDSTKYAEIYGIS-SAHTLLRGTLRYKGYMKALNGFVKLGLINREALPAFRPEA

----NPLTWKQLLCDLVGIS------------------PSSEHDVLKEAVLKKLGGDNTQ

LEAAEW-----LGLLGDEQ--VPQ----------AESILDALSKHLVMKLSYGPEEKDMI

VMRDSFGIRHPSG-HLEHKTIDLVAYGDI---------NGFSAMAKTVGLPTAMAAKMLL

DG--EIGAKGLMGPFSKEIYGPILERIKAEGIIYTTQSTIKP-

>Mus_musculus

TKKKVLVLGSGYVSGPVLEYLSRDNN--------------------IEITLGSDMTNQMQ

QLSKKY-NINPVSLTVGKQEAKLQSLVESQDLVISLLPYVLHPVVAKACIESRVNMVTAS

YITPAMKELEKSVDDAGITVIGELGLDPGLDHMLAMETIDTAKELGATVESYVSYCGGLP

APEHSDNPLRYKFSWSPVGVLMNIMQPASYLLNGKVVNVTGGVSFLNS-VTPMDYFPGLN

LEGYPNRDSIKYAEIYGIS-SAHTLLRGTLRYKGYSKALNGFVKLGLINREAYPALRPEA

----NPLTWKQLLCDLVGIS------------------RSSPCEKLKEVVFTKLGGDNTQ

LEAAEW-----LGLLGDEQ--VPQ----------AESIVDAFSKHLVSKLSYGPEEKDMI

VMRDSFGIRHPSG-HLENKTIDLVVYGDF---------NGFSAMAKTVGLPTAMAAKMLL

DG--EIEAKGLMGPFTKEIYGPILERIKAEGIVFNTQSTIKL-

>Monodelphis_domestica

NTKKVLLLGTGYVSEPVLEYLSRDGS--------------------IEITVGSDMKNQLE

HLAKKY-NINPIVLNIGKQEERLASLVKKQDLVISLLPYVLHPLVAKACIASKVNMVTAS

YITPAIKELEKSVDEAGITIIGELGLDPGLDHMLAMETIDKAKEVGATIESYVSFCGGLP

APEHSDNPLRYKFSWSPVGVLLNILQPAKYLLNGEVVD-AGGASFLDY-VNSMDYFPGLN

LEGYPNRDSIKYAEIYDIP-FAHTLLRGTLRYKGYAKALNGFLKLGLINRDAYPALSPEA

----SPITWKELLCELVGIS------------------PSSTPDVLKEAVYKKLDGDDMQ

LQAIEW-----FGLFGDEQ--VPR----------AGSLVDAFSKHLVMKLSYGPGEKDMI

VMRDSFGIRHPSG-HLEHKNIDLVVYGEV---------NGFSAMAKTVGLPAAMAAKMIL

DG--EIKAKGLIGPFTKEIYGPILERIKAEGIIYTVQSTIKQ-

>Danio_rerio

GMKRVLLLGAGYVSGPVIEYLTRDAG--------------------TQVTVASNLLNQAE

EMATRYPNTIAVMLDITSQEGHLESLIKDHDIVISMLPYSFHPQVAKHCIKLKVNMVTAS

YLSPAMKELQKSAEDAGITIVNEMGLDPGIDHMLAMECIDQAKADGCTVESYSSFCGGLP

APECSDNPLRYKFSWSPYGVLLNTISPAIYLKDNQIVSVPPGGALLDV-TKEMDFMPGFN

LEGFPNRDSTKYAEPYGIE-SAHTLIRGTLRFRGFSSAMSGFVKLGLINTEPCPLLDHTA

----SPVSWKELLCKQIGLS------------------ATVSNSVFEDAVYEKIGKDDFR

MQSLKW-----LGLLGEEP--VPH----------AETILAAVAKHLEAKLSFDKGERDMI

IMRNDVGIRHPTG-ELETKHISLVVYGDP---------SGYSAMAKTVGYPAAIAAHMVL

NG--ELTTKGLVVPMVKSIYSPVLKRLQDEGLQFITKSSVSE-

>Ciona_intestinalis

QNHKVLVLGAGFVSAPLIDYLTKHND--------------------TAVTVASNIKQEVK

NMSGKFKHASPIVLDILKETDRLTELVKGHDLVISLLPHTIHPPIAKLCIENKKNFVTAS

YVSPQIKELEKSALDAGISIVMEVGVDPGIDHMLAMQCFHDIKERGGNVSSFVSWCGGLP

SPEDSENPLKYKFSWSPAGMLQSAMAGSRYLKDGKVIEMAEGGDNYKYGRQDISFMPGFH

LEGFPNRDSIKYKDQYEIQ-EADTVFRGTLRYDGFVDSIIGLQQLGIVSSEMNSMLDEDS

----PDISWKQFISHILGGG------------------SDISEEDLQQLVYKCVDENDHR

LQTIIG-----LGLLSENI--VPK----------LKSPLQSLAAHLATKLTYGNDERDMI

VLRHEVIGQFNKG-KTEHHNIDLVVYGEP---------AGYTAMAATVGYPCAIAARMVL

LN--KIETKGVVTPLKKAVYKPLLKELKRLGIKPKISITTN--

>Caenorhabditis_elegans

TDKRVLLLGAGMVSGPFADFYSKQKD--------------------VNLTVATESQRDGQ

RLCTSP-NIQSVVVDIARESHTMERLIREHDLVVSLLPFNFHPLVAKMCISNQRDMVTSS

YVSPELEALDKAAKDADVTIMNEAGLDPGIDHMLAMECFDDIKEHGGRITSFESFCGGLP

APEWSDNPLRYKFSWSPKGVLTALMNPAKYLKNGKIVEVPSGS-VVDN-LIDIDFMPGLN

LIGFPNRDSTKYSDVYGLGNDCKTIIRGTLRYQGFVDTVKALHSVGLLSGDNIDSFTSNI

G---PDLTWKELIASLSNQK------------------LDIFPDSLRHIIEEKVGGKG--

LSALEN-----LGLFSDKV--VDR----------HGTPIDTLAQYLAKILAFKDHESDLV

VLNHDIGAQLPGG-NSERHRISLVQYGNP---------NGFSAMARTVGYTTAIVSHMVL

NN--EIQRAGIQRPILKEVYRPALKRLRDFGIVPTHTITPM--

>Drosophila_melanogaster

SDKKVLVLGAGMVSAPLVEWLHREKD--------------------VSITVCSQVKEEAD

RLAQQYAGVDSVYLDVNESTGHLQELCGRADVVVSLLPYSLHGMVARYCVAEGTHMVTAS

YLNDEISGLHEEAKAKGVTIMNEVGLDPGIDHLLALECIHEVQDKGAVVESFVSYCGGLP

APEHSNNALRYKFSWSPRGVLLNTLSAAKYLSQGQIVEISGGGELMSS-PRSLDFLPGFA

LEGFPNRDSTKYGNLYGLGRDVHTLLRGTIRYKGFSESIKPMQLLGLIDPEPHALLHPSG

----PDVTWRQLVIHLMGMS------------------DST---IFYENLKQKLTERIGD

VDGIES-----LGLLDDTP--VVK----------LNTPLDTLSHYLSKRLAFERDERDLV

VLRHEVGIRWPDG-RREERGINFVVYGQP---------QGHSAMAMTVGKPAAIAAKMIL

DG--EIQERGVLLPFTPDIYRPMLQRLRSEGLTATETSRWLN-

>Neurospora_crassa_OR74A

SQHKVLMLGAGFVTRPTLDVLSEAG---------------------IPVTVACRTLASAQ

KLSEGVKNAT-PISLDVTNDEALDAEVAKHDLVISLIPYTFHATVIKSAIRQKKHVVTTS

YVSPAMMELDAEAKAAGITVMNEIGLDPGIDHLYAIKTIDEVHQAGGKILSFLSYCGGLP

APEDSDNPLGYKFSWSSRGVLLALRNAGKWWQDGKIVEVE-GKDLMKMAKPYFI-YPGYA

FVAYPNRDSTIYKERYNIP-EAQTVIRGTLRYQGFPQFIKTLVDIGFLDDTARESLSK--

-----QTPWKEATKEIVGAAS--------------SSQADLEAAILSKATFESAEDQKRI

LSGLRW-----IGLFSDET-ITPR-----------GNPLDTLCATLEQKMQFEEGERDLV

MLQHKFEIEHADG-SRETRTSTLVEYGDP---------KGYSAMAKTVGVPCAVAVKQVL

SG--QISGKGVLAPMSTDITEPLMKELHEKYGITMIEKTIS--

>Sclerotinia_sclerotiorum_1980_

DTKKVLMLGAGFVTRPTLDILSEAG---------------------IQVSVACRTIESAK

KLSEGVKNAH-PISLDVTDDKALDAEVAKNDLVISLIPYTFHATVIKSAIRNKKNVVTTS

YVSPAMMELDEEAKNAGITVMNEIGLDPGIDHLYAVKTIEEVHKAGGKITSFLSYCGGLP

APEDSDNPLGYKFSWSSRGVLLALRNAARFYKDGKIVDVA-GPELMGTAKPYFI-YPGFA

FVAYPNRDSTPYKERYNIP-EAQTIIRGTLRYQGFPEFIRVLVDMGFLSDED-KGFSE--

-----PISWKEATQKVLAASS--------------SFEDDLKWAIASKAKFESTEEKERI

MNGLNW-----VGIFSDEK-IIPR-----------GNPLDTLCATLEKKMQFEKGERDLV

MLQHKFEIEHKDG-KKETRTSTLVEYGDP---------KGYSAMAKLVGVPCGVAVKQVL

DG--KISEKGILAPMTSKINDPLMEEL-KKYGITLVEKTIS--

>Aspergillus_niger

AGSKVLLLGSGFVTKPTVEVLSKAD---------------------VNVTVACRTLESAQ

KLCEGFKNTK-AISLDVTDDAALDKALEQVDLAISLIPYTFHANVIKSAIRTKKHVVTTS

YVSPAMMELDEECKKAGITVMNEIGLDPGIDHLYAVKTISEVHAEGGKITSFLSYCGGLP

APECSNNPLGYKFSWSSRGVLLALRNAAKFYKDGQEFSVA-GPDLMATAKPYFI-YPGFA

FVAYPNRDSCPYRERYNIP-EAQTVVRGTLRYQGFPEMIKVLVDIGFLSDEGREYLNT--

-----PIAWKEATKQILGATS--------------SAEKDLEWAIASKTAFANNDDRDRI

ISGLRW-----IGLFSDEQ-ITPR-----------GNPLDTLCATLEKKMQYEEGERDLV

MLQHKFEIEHKDG-QKETRTSTLCEYGVP---------GGYSAMAKTVGVPCGVAVKLVL

DG--TINQKGVVAPMTMDICAPLIKTLKEDYGIEMIEKTL---

>Pyrenophora_triticirepentis

SGKSALLLGAGFVTRPTVDVLAKSG---------------------VKVTVACRTLEKAQ

SLAKGIPNTN-AISLDVNDADALDAEVAKVDVVISLIPYTYHATVIKSAIRKKKNVVTTS

YVSPAMMELDAEAKEAGITVMNEIGVDPGVDHLSAVLTIDEVHKAGGKILSFKSYCGGLP

APENSDNPLGYKFSWSSRGVLLALRNQAKYYQDGQIKEVE-GPELMAEAKPYFI-YPGYA

FVAYPNRDSTPYKERYNIP-EAQTIIRGTLRYGGFPEYIKCLVDIGFLSEEPKDFLKEGE

-----KRTWRDATAKIIGASS--------------DKDEDLIWAISSRTKFASTEEKDRI

ITGLRW-----IGLLSDEE-ITPR-----------GNPLDTLCATLEKKMQYEEGERDFV

MLQHRFEIENKDG-SKSVRTCTLAEYGDP---------KGYSAMAKLVGVPCAVAVQQVL

DG--TLSEKGIIAPMSPEICAPLMKTLEDKYGVVFKEKTVPL-

>Saccharomyces_cerevisiae_S288c

MGKNVLLLGSGFVAQPVIDTLAANDD--------------------INVTVACRTLANAQ

ALAK-PSGSK-AISLDVTDDSALDKVLADNDVVISLIPYTFHPNVVKSAIRTKTDVVTSS

YISPALRELEPEIVKAGITVMNEIGLDPGIDHLYAVKTIDEVHRAGGKLKSFLSYCGGLP

APEDSDNPLGYKFSWSSRGVLLALRNSAKYWKDGKIETVS-SEDLMATAKPYFI-YPGYA

FVCYPNRDSTLFKDLYHIP-EAETVIRGTLRYQGFPEFVKALVDMGMLKDDANEIFSK--

-----PIAWNEALKQYLGAKS--------------TSKEDLIASIDSKATWKDDEDRERI

LSGFAW-----LGLFSDAK-ITPR-----------GNALDTLCARLEELMQYEDNERDMV

VLQHKFGIEWADG-TTETRTSTLVDYGKV---------GGYSSMAATVGYPVAIATKFVL

DG--TIKGPGLLAPYSPEINDPIMKELKDKYGIYLKEKTVA--

>Gibberella_zeae_PH-1

----ALLLGSGFVATPAVEVLSKAG---------------------VHVTVACRTLASAK

NLAGTFDNTK-AVSLDVNDSAALEQAVSEHDITISLIPYTFHAAVIKAAIKAKKNVVTTS

YVSPAMEELHEEAKAAGITVLNEIGVDPGVDHLYAVDFIDRIQQEGGKIKSFKSYCGGLP

APENSNNPLGYKFSWSSRGVLLALKNNAKYYEDNKLVDIS-GVDLMSTAQPYHSGYLGFN

FVAYGNRDSTGYRERYRIP-DAETVVRGTMRYNGFPQFVKALVDIGFLSTDEQDFFKQ--

-----SIPWKDALQKFIGANS--------------SSEEDLTKAILSKTSFKDESVKNQV

LAGLKW-----IGVFSDVK-TTPR-----------GTALDTLCASLEQKMAYEKGERDIV

FLQHTFEVINKDG-SQNTWTSTLVEYGAPEGS------GGFSAMSRLVGVPCGVATKMVL

DG--TITDKGVVAPVYPSLARTLMNELKNNYGIECKEKIIA--

>Schizosaccharomyces_pombe

----ILLLGSGFVAHPTLEYLSRRKE--------------------NNITVACRTLSKAE

AFINGIPNSK-AIALDVNDEAALEKAVSEHDLTISLIPYTYHATVMKAAIKHGKHVCTTS

YVNPKMAELEEAAIKAGSICMNEIGVDPGIDHLYAIKTIEEVHKAGGKIKSFLSYCGGLP

APEDSNNPLGYKFSWSSRGVLLALRNSAKFYENGKLVEID-GKDLMETAKPYFI-YPGYA

FVCYPNRDSTVYQERYQIP-EAETIIRGTLRYQGFPEFIHCLVDMGFLDETAQEYLSPEA

----PALPWKEVTARVIKAES--------------SSEADLIKKISSIHKFKDEDDKKRI

LNGLKW-----LGMFSSKP-VTPR-----------GNPLDTLCATLEELMQYEEGERDML

ILQHKFEVETKEG-KRQTRTCTLLDYGVP---------NGYTSMAKLVGVPCGVATQQIL

DG--VINTPGVLAPNDMKLCGPLIDTL-AKEGIRLEEEIIDEE

>Puccinia_graminis

PGKKILLLGSGFVAQPAADYLLRRPE--------------------NQVTVASFNLWKAE

RFATELAREVKCISLDINNSEALDKAVSEHDLVISLVPYIHHASVIKSAIKFKKNVVTTS

YVSPAMRALDEEVKKAGITVLNEIGVDPGVDHLYAVKMIDTVHRAGGKIIEFISYCCGLP

APECSNNPLGYKFSWSSRGVLLALLNSAKLYSKGKLIEIE-GQELMNHAQPYFI-SPAFA

FVAYPNRDSTPFREFYEIP-EAQTVVRGTIRYQGFPAFIKTLVDIGFLNETPQAYLRPES

-----TLPWKEVTARILGADD--------------STERCLVSEIKKRTTFPNSDEEVRI

LAGLKW-----IGIFSDDH-AVPR-----------GNILDTLCARLETKMQYEKDERDMV

VLQHKFGIQWQDG-SKETRTSTLVEYGAPFQSG-----TGPSAMAKLVGVPCGIAVQLIL

DG--KITKKGVLAPYTLDIVAPLLAEV-EKEGISMVDQIVS--

>Ustilago_maydis_521

APKKILLLGSGYVAGPFAQYVTRFPE--------------------YSLTVASSKLEHSE

RLTQGLHNAS-AAAVDVNDAAALSALVKGHDIVISLIPYIYHAAVIKAACEHKVNVVTTS

YVSDAIRALEPEIQKAGITVMNEIGLDPGLDHLYAVKAIDDVHAEGGKIKSFLSYCGGLP

APEAADNPLGYKFSWSSRGVLLALRNTAKFWQDGQELTVS-GHELMAAAKSFYI-NPAFA

FVAYPNRDSTPFKQWYNIP-EAETVIRGTLRYQGFPEFILALVKLGFLDEESKPFLAYNT

-----KATWAEVTANMVGASS--------------TSETDLIAAVKAKVSFKSAQEEETI

IRGLRW-----LDLFSTTAPVTVRGTAAQEAAQVAGNPLDSLCATLEDKCAYAPGERDMV

MLQHKFEIETASG-EHKTLTSTLLDYGIP---------HGVTSMAKLVGVPCAIATRLIL

EGHPALSKTGILAPYTKDICDPIRLEL-EKEGIALEERYV---

>Sorghum_bicolor

GGPKVLILGAGRVCRPAAEFLASYPNICT-------YGVDDHNTDQIHVIVASLYQKDAE

ETVDGIENTT-ATQLDVSDIGSLSDLVSQVEVVISLLPTSFHAAIARVCIELKKHMVTAS

YVDESMSNLSQAAKGAGVTILCEMGLDPGIDHLMSMKMIDEAHARKGKIKTFTSYCGGLP

SPAAANNPLAYKFSWNPAGALRAGKNPAVYKFLGETIHVDGHNLFESAKRLRLPELPAFA

LEHLPNRNSLIYGDLYGISKEASTIYRATLRYEGFSEIMATLSKIGLFDAANHPLLQET-

----NRPTYKGFLDELLNNISTTNTGLDIEASGGYD--DEIIARLSKLGCCRDKEIAAKT

VKTIKF-----LGLHEETQ--IPKG---------CSSAFDVICQRKEQRMAYGHNEQDMV

LLHHEVEVEYPDGQPTEKHQATLLEFGKVEN------GRSTTAMALTVGIPAAVGALLLL

QN--KVQTKGVIRPLQPEIYIPALEILESSGIKLIESVEI---

>Zea_mays

GGPKILILGAGRVCRPAAEFLASYPDICT-------YGVDDHDADQIHVIVASLYQKDAE

ETVDGIENTT-ATQLDVADIGSLSDLVSQVEVVISLLPASFHAAIAGVCIELKKHMVTAS

YVDESMSNLSQAAKDAGVTILCEMGLDPGIDHLMSMKMIDEAHARKGKIKAFTSYCGGLP

SPAAANNPLAYKFSWNPAGALRSGKNPAVYKFLGETIHVDGHNLYESAKRLRLRELPAFA

LEHLPNRNSLIYGDLYGISKEASTIYRATLRYEGFSEIMVTLSKTGFFDAANHPLLQDT-

----SRPTYKGFLDELLNNISTINTDLDIEASGGYD--DDLIARLLKLGCCKNKEIAVKT

VKTIKF-----LGLHEETQ--IPKG---------CSSPFDVICQRMEQRMAYGHNEQDMV

LLHHEVEVEYPDGQPAEKHQATLLEFGKVEN------GRSTTAMALTVGIPAAIGALLLL

KN--KVQTKGVIRPLQPEIYVPALEILESSGIKLVEKVET---

>Populus_trichocarpa

RKAAVLIIGAGRVCRPAVELLTSNENTSSREWYKACLNTDFEGQNVVEVVVASLYLKDAE

EIIDGIPNAS-AVQLDVMDDESLCKYISQVEVVVSLLPPSCHIIIANACIKLKKHLVTAS

YVDDSMSFLHEEAKAADITILGEMGLDPGIDHMMAMKMINNVRVRKGRIKSFTSYCGGLP

SPAAANNPLAYKFSWSPAGAIRSGRNPATYKNHGEIVHVDGEKLYDSAFRFRLPNFPAFA

LECLPNRNSLVYGKLYGIEDEASTIFRGTLRYEGFGEIMGTLASIGLFNTESHLVLRHG-

----QRPSFKRFLCELLNIVSEIPDGVPLGEKH-------ISERIVALGHCKEQGTAVRT

AKTIIY-----LGLHEQTE--IPVS---------CQSAFDVTCYRMEERLAYSSTEQDMV

LLHHEMEVEFPDSQATENHKGTLLEFGRTGN------GKTTTAMALTVGIPVAIGALLLL

EN--KINTRGVLRPFEPEVYVPALDILQAYGIKVMEKVE----

>Vitis_vinifera

EKPGVLILGAGRVCQPVAEVLTTAGSVSSRQLFKMCQESDFEGQSDIQVIVASLYLKDAE

EIIEGLPNAT-AIQLDVMDHENLHKYISQVEVVISLLPASCHFIVANACIELKKHLVTAS

YIDDSMSKLDERAKGAGITILGEMGLDPGIDHMMAMMMIDQAHVQGGKIRSFISYCGGLP

SPEAANNPLAYKFSWNPAGAIRSGRNPATYRSHGETVSINGESLYDSAVSFRIPDLPAFA

LEILPNRNSLVYGDLYGIDHEASTIFRGTLRYEGFAEIMGTLARIGFFDTEAHPILTLT-

----KRPTFGAFLLELLKIKSEDFDGT-MTAED-------IKERILALGLCKVQVTALKT

AKTILY-----LGFHEQTE--IPVS---------CRSAFDVACLRMEERLAYSSEEQDMV

LLHHEVEVEFPDGRPVEKHRATLLEFGKTKN------GKTTTAMAFTVGIPAAIGALLIL

EK--KIKTRGVLRPIEPQVYVPALDILQAYGLKLLEKTE----

>Arabidopsis_thaliana

KKSGVLILGAGRVCRPAADFLASVRTISSQQWYKTYFGADSEEKTDVHVIVASLYLKDAK

ETVEGISDVE-AVRLDVSDSESLLKYVSQVDVVLSLLPASCHAVVAKTCIELKKHLVTAS

YVDDETSMLHEKAKSAGITILGEMGLDPGIDHMMAMKMINDAHIKKGKVKSFTSYCGGLP

SPAAANNPLAYKFSWNPAGAIRAGQNPAKYKSNGDIIHVDGKNLYDSAARFRVPNLPAFA

LECFPNRDSLVYGEHYGIESEATTIFRGTLRYEGFSMIMATLSKLGFFDSEANQVLSTG-

----KRITFGALLSNILNKDADNESEPLAGEEE-------ISKRIIKLGHSKE--TAAKA

AKTIVF-----LGFNEERE--VPSL---------CKSVFDATCYLMEEKLAYSGNEQDMV

LLHHEVEVEFLESKRIEKHTATLLEFGDIKN------GQTTTAMAKTVGIPAAIGALLLI

ED--KIKTRGVLRPLEAEVYLPALDILQAYGIKLMEKAE----

>Physcomitrella_patens

KNYQVLILGAGRMCEPAVRHLTSTKRRFR--FREASYVDNGLVDECVSVVVASLYIEDAQ

RVVAGVPNAS-AIELDTSDTQNLGDCVSKANVVISLLPADLHLPVATACIKFKKHLVTAS

YVSEAMQALDKMAKEADVTLLCEMGLDPGIDHLMAMKMIDSAHKRGGRVQSFVSYCGGLP

SPEAANNPLGYKFSWNPAGAIKAGRNPAVYLMNGQKIEVPGEKLFAAALPVRLRDTPAFA

LERLPNRDSLKYGELYGISKEASTIFRATLRYQGFSKVMDALGELGYFDTDLHPLLDPGA

LPVPDQPTYNAMLDALLLQMFHKMNEPEKRMMLNAGDRNSLSHALAYLPCCKNNPSAVED

AAACIRQVSSWLGLDALEQ--VPKS---------CKSIFEVLCKRMEEKLTYGPEETDMV

LLHHELDVELNG--QVERHTATLLAFGETAEGCSQ--RRPESAMARTVGIPAAIAAELLL

FG--EVKTRGVLRPLTAEIYEPALEVLKTMKLPLVEHVEFL--

>Selaginella_moellendorffii

EYRNILILGAGRMCEPTLMYLTEN--------AFEDYADTSKPPKQVFVHVGSLYLEDAS

KVVEGVENAL-AIQIDVMDEQQLESQVQKVEVVISLLPPSFHERVAVACIELKKHLVTAS

YVSKDMALLDSRAQAAGVTLLCEMGLDPGIDHMMAMKMIDASHERGGKVRVFESYCGGLP

SPEAANNPLAYKFSWNPTGAIKAGRNAAIYKHENKIIRVPGERLFGAAVSFRIPQYPAYA

LEVLPNRDSLMYGDLYGITQEAATIFRGTLRYEGFGQIMDTLGKLGYYNSDNHPLLAS--

---STETTYAAVLEALILQLSTSYN-------------GLCAEELARIISS-DNLDVAKR

VLSCIR----FLGLDSQEI--VPRS---------CKSAFEVLCSRMEEKLVFRANEQDLV

LLHHELEVVYEDSRSAERHSATLVAVGESCNQLKNESRRPHSAMARTVGLTVAIGAELLF

TG--RLKSRGVIRPLQPEVYVPG------MSYDFVQTN-----

**CTH.fasta**

>Sorghum_bicolor

----------------------MAAAVPTAPARLFLLQSTPSPNPSSAAAAAQAPVLRVP

PLRLSLSRRMAGRPLTVIAGASGDSERDISASAVSLEALDSVAS-DSDLEAKEPSVATML

TSFENSFDKYGALSTPLYQTATFKQPSATDYGP---------------------------

---------YDYTRSGNPTRDVLQ----SLMAKLEKADQ-------------AFCFTSGM

AALAAVTHLLK-AGQEIVAGEDIYGGSDRLLSQVVPR-NGIVVNRVDTT-----------

NISDVVSAIGP-STRLVWLESPTNPRQQITDIKTISEIAHSHG-----ALVLVDNSIMSP

VLSR---PIELGADIVMHSATKFVAGHSDLMAGILAVKGES-------------------

-----------------------------------LAKEIAFLQNAEGTGLAPFDCWLCL

RGIKTMALRVEKQQANAQKIAEFLASHPR----VKQVNYAGL-P-----DHPGRSLHYSQ

AKGAGS------VLSFLTGS--------------------LALSKHVVETTKHFSVTVSF

GSVKSLISLPCFMSHAAIPAAVREERGLTDDLVRISVGIE-DVEDLIADLDRALRTSPA-

------------------------------------------------------------

------------------------------------------------------------

------------------------------------------------------------

------------------------------------------------------------

------------------------------------------------------------

------------------------------------------------------------

------

>Zea_mays

----------------------MAVAVPNAPGRLFLLQSTPFPNPSSSASAARAQSFRVP

PLRLSLFRRMAGRSLTVIAGASGGSERDLSASAVSVEALDSVAS-DSDLETKEPSVSTML

TSFENSFDKYGALSTPLYQTATFKQPSATDYGT---------------------------

---------YDYTRSGNPTRDVLQ----SLMAKLEKADQ-------------AFCFTSGM

AALAAVTHLLQ-AGQEIVAGEDIYGGSDRLLSQVVPR-NGIVVKRVDTT-----------

KISDVVSAIGP-STRLVWLESPTNPRQQITDIKTISEIAHSHG-----ALVLVDNSIMSP

VLSR---PIELGADIVMHSATKFIAGHSDLMAGILAVKGES-------------------

-----------------------------------LAKEVGFLQNAEGSGLAPFDCWLCL

RGIKTMALRVEKQQANAQKIAEFLASHPR----VKQVNYAGL-P-----DHPGRALHYSQ

AKGAGS------VLSFLTGS--------------------LALSKHVVETTKYFSVTVSF

GSVKSLISLPCFMSHASIPASVREERGLTDDLVRISVGIE-DVEDLIADLDRALRTGPV-

------------------------------------------------------------

------------------------------------------------------------

------------------------------------------------------------

------------------------------------------------------------

------------------------------------------------------------

------------------------------------------------------------

------

>Oryza_sativa

--------------MSAAAAAAAAAAIPTSLGRLFHLRPTPNPSRNLSGSSAQPLLRLSY

HPRLTLSRRMEAP--AAIADSHGGG--DLSASAVGAEALGAVAAPDFDVEMKEPSVATIL

TSFENSFDGFGSMSTPLYQTATFKQPSATDNGP---------------------------

---------YDYTRSGNPTRDVLQ----SLMAKLEKADQ-------------AFCFTSGM

AALAAVTHLLK-SGQEIVAGEDIYGGSDRLLSQVAPR-HGIVVKRIDTT-----------

KISEVTSAIGP-LTKLVWLESPTNPRLQITDIKKIAEIAHYHG-----ALVLVDNSIMSP

VLSR---PLELGADIVMHSATKFIAGHSDLMAGILAVKGES-------------------

-----------------------------------LAKEIAFLQNAEGSGLAPFDCWLCL

RGIKTMALRVEKQQANAQKIAEFLASHPR----VKKVNYAGL-P-----DHPGRSLHYSQ

AKGAGS------VLSFLTGS--------------------LALSKHVVETTKYFNVTVSF

GSVKSLISLPCFMSHASIPSAVREERGLTDDLVRISVGIE-DADDLIADLDHALRSGPA-

------------------------------------------------------------

------------------------------------------------------------

------------------------------------------------------------

------------------------------------------------------------

------------------------------------------------------------

------------------------------------------------------------

------

>Physcomitrella_patens

------------------------------------------------------------

--------------------------MDEEKRFVTNLHLKFVVVFFKKSERK-LHVSTQL

QQLDNPFDPYGALSTPLYQTATFKQPSATEGGP---------------------------

---------YDYTRSGNPTRDALERQVCRTLAKLEGADR-------------AFCFSTGM

AALATVMRLAE-AGQEIVAGDDIYGGSDRLLSQVAPK-SGLIVKRIDTG-----------

NLDEVRAAVGP-KTKIVWLESPTNPRQMISDIREIAKIAHEYS-----ALVIVDNSIMSP

VLSR---PLDLGADIVMHSATKFIAGHSDVMAGVLAVKGES-------------------

-----------------------------------LAKEIYFLQNAEGTGLSPFDCWLCL

RGIKTMALRVEKQQANAQRIAEFLVDHPL----VTRVNYAGL-P-----KHTGRDLHYSQ

CKGAGS------VFSFNTGS--------------------LAISKHVVEQTKLFSITVSF

GSVKSLISLPCFMSHASIPAGVREARGLTEDLIRISIGIE-DVDDLVADLEQALTTAPK-

------------------------------------------------------------

------------------------------------------------------------

------------------------------------------------------------

------------------------------------------------------------

------------------------------------------------------------

------------------------------------------------------------

------

>Schizosaccharomyces_pombe

------------------------------------------------------------

----------------------------------------------MPSDCK-YSVDTEL

VHVEGNEDQYHASSVPIYQSATFKQPCLEHMGK---------------------------

---------FDYTRSGNPTRSVLQ----VHLAKLMKAKH-------------AFVTSNGM

SALDMILRCCK-SNSHVVAGHDLYGGSDRLLSFNQRQ-YGFKVDNVDTS-----------

DLAAFEAALRP-DTNLVLIESPTNPRISICDIRAIVKITRSKAK---DALLVMDNTMLSP

VLCN---PLDFGYDIVYESATKYLSGHHDLMGGVIATKSDE-------------------

-----------------------------------IAKSVFFNINAMGAAMAPFECFLLL

RGIKTMGLRVERAQQNAIEIAKFLKSKG------LQVNFPGLDP-----DAKSTAIFYSF

ARGPGA------VMSVFTGD--------------------VEVSKTIVNTTKLFEISVSF

GAVNSLISMPAYMSHASIKKEVRDARGLSEDLIRICVGIE-NVDDLKADLENALAQANFK

QN----------------------------------------------------------

------------------------------------------------------------

------------------------------------------------------------

------------------------------------------------------------

------------------------------------------------------------

------------------------------------------------------------

------

>Neurospora_crassa_OR74A

------------------------------------------------------------

-------------------------------MTASS-DHPITTPPRAPSPVHGFGTLAVH

AGSPHDP-ATGAVIEAISLSTTFAQTAVGKPVGE--------------------------

---------FEYSRSSNPNRANFEK----MVAALEHAKY-------------ALAYSSGS

ATTANILQSLA-AGSHVISVSDVYGGTHRYFTQVAKA-HGVKVTFT-----------PEI

EVD-IRDHITD-ATKLVWIETPSNPTLRLVDIRAVATAAHERG-----ILVVVDNTFLSP

YVQN---PLDHGADIVVHSVTKYINGHSDVVMGVAAFNSDE-------------------

-----------------------------------LYARLSFLQNAIGAVPSAFDSWLAH

RGAKTLHLRAREATTNATAIAHALEAS----PLVISVNYPGLES------HPHRAIALKQ

HRNGMG-------------GGMLSFRIH--GGH--------AAAEKFCQYTKIFTLAESL

GGVESLCEIPSSMTHAGIPKAQREAVGIFDDLVRISCGVE-DAEDLKADVLQALERA---

-------------VADAANG--VSNGVNGTH-----------------------------

------------------------------------------------------------

------------------------------------------------------------

------------------------------------------------------------

------------------------------------------------------------

------------------------------------------------------------

------

>Sclerotinia_sclerotiorum_1980_

------------------------------------------------------------

-------------------------------MTIPSFDHPIETPPRAPSPVHRFGTLAVH

AGSPHDP-VTGAVIEAISLSTTFAQTAVGKPVGA--------------------------

---------YEYSRSSNPNRDNFEQ----AVAALEHAKY-------------ALAFSSGS

ATTATILQSLA-AGSHVISVSDVYGGTHRYFTQVAKA-HGVKVTFT-----------PEI

EVD-VVDHITP-ETKLIWIETPSNPTLRLVDIRAVATAAHEHG-----IMVVVDNTFMSP

YVQQ---PLDHGADIVVHSVTKYINGHSDVVMGVAAFNSDA-------------------

-----------------------------------MKERLGFLQNAIGCIPSAFDSWLAH

RGLKTLHLRAREATVNATLVAKTLESS----PHVIAVNYPGLDS------HPHRKIALKQ

HRDGMG-------------GGMLSFRIK--GGS--------AAAEKFCQMTNIFTLAESL

GGVESLVEVPSSMTHAGIPKAHREAIGVYDDLVRISCGVE-DAEDLKADVLQALEKA---

-------------VVWPK----ISNGLENYTS----------------------------

------------------------------------------------------------

------------------------------------------------------------

------------------------------------------------------------

------------------------------------------------------------

------------------------------------------------------------

------

>Gibberella_zeae_PH-1

------------------------------------------------------------

------------------------------MSSAPLNSDPVATPPRPPSPVHNFGTLAVH

AGAPHDP-ATGAVIESISLSTTFAQSAVGKPVGE--------------------------

---------YEYSRSSNPNRTNFET----AVAALEHAKY-------------ALAFSSGS

ATTATILQSLA-AGSHVISVSDVYGGTHRYFTQVAKA-HGVKVTFT-----------PEI

EVD-IAEHITP-DTRLIWIETPSNPTLRLVDIRAVVTEAHKHG-----VLVVVDNTFLSP

YVQN---PLDFGADIVVHSVTKYINGHSDVVMGVAAFNSDE-------------------

-----------------------------------LKARLSFLQNAIGAVPSAFDSWLAH

RGLKTLHLRAREASRNADAVARALEAS----PLVIAVNYPGLDS------HPHRHIAKKQ

HRDALG-------------GGMLSFRIQ--GGH--------AAAERFCQVTKIFTLAESL

GGVESLVEVPSSMTHAGIPRDQREAVGIFDDLVRISCGVE-DAQDLTNDVLQALEKA---

-------------TSAAKANGLNGNGVNGSNGH---------------------------

------------------------------------------------------------

------------------------------------------------------------

------------------------------------------------------------

------------------------------------------------------------

------------------------------------------------------------

------

>Pyrenophora_tritici-repentis

------------------------------------------------------------

---------------------------------MTVSEHPIDTPPRAPSPVHRFGTLAVH

AGSPHDP-VTGAVIESISLSTTFAQTSVGKPVGE--------------------------

---------YEYSRSSNPNRDNFEK----AVAALEHARY-------------ALAFSSGS

ATTANILQSLA-AGSHVVSVSDVYGGTHRYFTKVALT-HDVKVTFS-----------PSI

EID-IAELIRP-NTKLIWIESPSNPTLTLVDIRKISTIAHQHG-----IMVVVDNTFMSP

YVQN---PLDHGADIVVHSVTKYINGHSDVVMGAAAFNSDD-------------------

-----------------------------------LYERLSFLQNAIGAVPSAFDCWLAH

RGLKTLHLRAREATKNATGVAKALEAS----SHVISVNYPGLPS------HPQHAIALKQ

HRDGMG-------------GGMLSFRIQ--GGH--------EAAERFCQATKIFTLAESL

GGVESLVEVPSAMTHGGIPKEQREAAGVFDDLVRISCGVE-DEVDLVADVKQALEKA---

-------------LVGPK----VKNGVE--------------------------------

------------------------------------------------------------

------------------------------------------------------------

------------------------------------------------------------

------------------------------------------------------------

------------------------------------------------------------

------

>Aspergillus_niger

------------------------------------------------------------

-------------------------------MVALPTQNGASTPQD-----RRFGTLAVH

AGAPHDP-TTGAVIAPISLSTTFAQTSVGNPVGL--------------------------

---------YEYTRSSNPNRDNFEQ----AIAALEHAKY-------------ALAFSSGS

ATTAVILQSLA-AGSHIVSVSDVYGGTHRYFTKVASA-HGVQVTFS-----------PCI

ELD-VEELIRPNETKLVWIETPSNPTLGLVDIEKIAAVAHKYG-----ILVVVDNTFLSP

YVQN---PLDHGADIVVHSVTKYINGHSDVLMGVAAFNSDS-------------------

-----------------------------------LQERLSFLQNAIGAVPSPFDCWLAH

RGLKTLHLRAREATTNATAVAKALEAS----PNVIAVNYPGIDS------HPNRAIAVKQ

HRNGMG-------------GGMLSFRIK--GGQ--------EAAHLFCKYTKIFTLAESL

GGVESLCEVPSSMTHAGIPKDQREAAGVFDDLVRMSCGVE-DSEDLLADALQALEKA---

-------------VAATQ----VENGSA--------------------------------

------------------------------------------------------------

------------------------------------------------------------

------------------------------------------------------------

------------------------------------------------------------

------------------------------------------------------------

------

>Saccharomyces_cerevisiae_S288c

------------------------------------------------------------

-----------------------------------------MTLQES----DKFATKAIH

AG-EHVD-VHGSVIEPISLSTTFKQSSPANPIGT--------------------------

---------YEYSRSQNPNRENLER----AVAALENAQY-------------GLAFSSGS

ATTATILQSLP-QGSHAVSIGDVYGGTHRYFTKVANA-HGVETSFT-----------NDL

LND-LPQLIKE-NTKLVWIETPTNPTLKVTDIQKVADLIKKHAAG-QDVILVVDNTFLSP

YISN---PLNFGADIVVHSATKYINGHSDVVLGVLATNNKP-------------------

-----------------------------------LYERLQFLQNAIGAIPSPFDAWLTH

RGLKTLHLRVRQAALSANKIAEFLAADK---ENVVAVNYPGLKT------HPNYDVVLKQ

HRDALG-------------GGMISFRIK--GGA--------EAASKFASSTRLFTLAESL

GGIESLLEVPAVMTHGGIPKEAREASGVFDDLVRISVGIE-DTDDLLEDIKQALKQA---

-------------TN---------------------------------------------

------------------------------------------------------------

------------------------------------------------------------

------------------------------------------------------------

------------------------------------------------------------

------------------------------------------------------------

------

>Puccinia_graminis

------------------------------------------------------------

----------------------------MSNPPQDKSGHGGAELLTSNPHGDHLHTRLIH

VGAHPDP-TTGAVIRPISLSSTFAQSAVGVHAG---------------------------

---------YEYSRSLNPNRLDLET----LIASLEGVDQLERVEPAGLPLPGALAVSSGS

AATATIINALVGPGAHLVAMSDVYGGTYRYLNQVAKQ-LGIDSTMIDLGFADDSKQAAEL

VIERLQKSIIPGQTKLIWIETPTNPTLRLVDIACISDFARKNG-----LLLVVDNTFLSP

YYQQ---PLRLGADIVVHSATKYINGHSDVVLGVIVSPHVE-------------------

-----------------------------------LIKKMRFLQNAHGAVPSAFDCWLAQ

RGLKTLALRMLRHGTNALAIANWLQDVALPQGWITKVTYIGLNKSEIAWRQIPKETQEEL

HKLGHGPS----ADGQFPYGGMISFKIKPKGGDGLDGQTDYSRADEFLKNCRLFTLAESL

GGIESLASLPAKMTHATLSVEDRSKLGIDGSLVRLSVGIE-KVEDLIADLEQSFKKS---

-------------F----------------------------------------------

------------------------------------------------------------

------------------------------------------------------------

------------------------------------------------------------

------------------------------------------------------------

------------------------------------------------------------

------

>Ustilago_maydis_521

------------------------------------------------------------

------------------------------MTISSTNGVSNDNGHADVLVSSGFSTKAIH

VGSEPNA-ATGAVIPPISLSSTFAQDGIGNHKG---------------------------

---------YEYSRSGNPNRDSFER----ALAALEGGSR-------------GLAFSSGS

AVTATVLNSMP-SGSHIVSVNDVYGGTYRYFTKVATVSQGIRTSFVEMD-------GPEA

EVSERVNAAIRKETSLVWIETPTNPTLRVIDVPLIVKLVRNHPNASKDIKIVVDNTFMSP

WFQN---PLAHGADLVMHSVTKYLNGHSDVVMGAAVTNDEA-------------------

-----------------------------------WADRLAFLQNSIGAIPSPFDCWLAL

RGLKTLALRMKAHGASALAIAQFLENH----PSVETVIYPGLPS------HPGHEVARRQ

ISKRAAAAGPSNSNGAFAYGGMISFRIKSNPADD-------EPADKLLSNLNVFTLAESL

GGVESLIELPSKMTHGSVALEDRIKIGIGHNLIRVSVGIE-ETADLIQDLERGLKAAGFI

LPLQAVTVILDAFVSTPSCVAEKHNFAADIGDAIASHLESLDSCPKTRQKLRLCSLETLS

HHFAQPGLASSETCTSRRSISEAFGLLAGSTASSRTGLRRRNISTWSHPSRSFSSTSSNR

DLKQDTKDKLVEEGKKFAKDKLDSKLNATETREQAKNLSSSVSKILFTLLAVAGVAYQFV

GGKTHAEEACTSLKPAPKYTKHQVSLLCLVGPSLSGKSTQAKRLSNHFPELDAVVQPKSI

DELSSIIASRATGKKRTSLILDDFPTTLEDAQRIEDEIVPIFCFSFYDLPLGDFEKRLSE

KDEKKKREKVDEFNKYSERLEPLVKKYRHQGNIYEISADWNSADEVWEQVEAKTQQILEL

RERGDL

>Xenopus_tropicalis

------------------------------------------------------------

-------------------------------------------MDGFQEGFKHFATEAIH

AGQEPEQWKSMAVVPPITLSTTFKQFGPGEHAG---------------------------

---------YEYSRSGNPTRNSLEK----AVAALDGAKY-------------CLSYASGL

AATMNIAHLLK-AGDQIICTKDVYGGTNRYFRRVASE-MGLKVAFVDCS-----------

DLKSLEAAIAA-NTKLVWIETPTNPTLTVIDIKGCADIIHKH----KDIILAVDNTFLSA

YFQR---PLALGADICMYSATKYMNGHSDVVMGLVSVNCDK-------------------

-----------------------------------LYEKLKFLQNAIGAVPSPFDCYLCN

RGLKTLHLRMQQHFQNALAVAKFLEAD----PRVDKVLYPGLPS------HPQYELTKRQ

CTGVSG---------------MITFYIKG----------NLEHATTFLKSLKVFALAESL

GGYESLAEHPAIMTHAAVPEEDRAALNISDTLIRLSIGLE-DVEDIIGDLDQALRSAHPD

IKNEN-------------------------------------------------------

------------------------------------------------------------

------------------------------------------------------------

------------------------------------------------------------

------------------------------------------------------------

------------------------------------------------------------

------

>Danio_rerio

------------------------------------------------------------

--------------------------------------MQNDSSAGFLPIYKSFATDAIH

VGSEPEQWSSMAVVPPISLSTTFKQHGPGKHAG---------------------------

---------FEYSRSGNPTRNCLEK----AVAALDGAKY-------------CLAVASGL

AATLTITHLFK-SGDGILCMNDVYGGTNRYFRKVASE-VGLDISFADLT-----------

KLEELKAGLKP-NTKMVWIETPTNPTMKVVDIQACADIVHKHN---KDTIVVVDNTFMSA

YFQR---PLALGADICMYSATKYMNGHSDVVMGLISLSRDD-------------------

-----------------------------------LYERLKFLQNSLGAVPSPFDCYMCN

RGLKTLHLRMKQHFKNALAAAQFLEAD----PRVDRVIFPGLPS------HPQHELTKRQ

CTGCPG---------------MITFYIKG----------KLEHASTFLSNLKLFALAESL

GGYESLAEHPAIMTHASVCEEERKKLGISDTLIRLSVGLE-DEEDIIADLDQALGAAHPK

K-----------------------------------------------------------

------------------------------------------------------------

------------------------------------------------------------

------------------------------------------------------------

------------------------------------------------------------

------------------------------------------------------------

------

>Homo_sapiens

------------------------------------------------------------

------------------------------------MQEKDASSQGFLPHFQHFATQAIH

VGQDPEQWTSRAVVPPISLSTTFKQGAPGQHSG---------------------------

---------FEYSRSGNPTRNCLEK----AVAALDGAKY-------------CLAFASGL

AATVTITHLLK-AGDQIICMDDVYGGTNRYFRQVASE-FGLKISFVDCS-----------

KIKLLEAAITP-ETKLVWIETPTNPTQKVIDIEGCAHIVHKH----GDIILVVDNTFMSP

YFQR---PLALGADISMYSATKYMNGHSDVVMGLVSVNCES-------------------

-----------------------------------LHNRLRFLQNSLGAVPSPIDCYLCN

RGLKTLHVRMEKHFKNGMAVAQFLESN----PWVEKVIYPGLPS------HPQHELVKRQ

CTGCTG---------------MVTFYIKG----------TLQHAEIFLKNLKLFTLAESL

GGFESLAELPAIMTHASVLKNDRDVLGISDTLIRLSVGLE-DEEDLLEDLDQALKAAHPP

SGSHS-------------------------------------------------------

------------------------------------------------------------

------------------------------------------------------------

------------------------------------------------------------

------------------------------------------------------------

------------------------------------------------------------

------

>Pan_troglodytes

------------------------------------------------------------

------------------------------------MQEKDASSQGFLPHFQHFATQAIH

VGQDPEQWTSRALVPPISLSTTFKQGAPGQHSG---------------------------

---------FEYSRSGNPTRNCLEK----AVAALDGAKY-------------CLAFASGL

AATVTITHLLK-AGDQIICMDDVYGGTNRYFRQVASE-FGLKISFVDCS-----------

KIKLLEAAITP-ETKLVWIETPTNPTQKVIDIEACAHIVHKH----GDIILVVDNTFMSP

YFQR---PLALGADICMYSATKYMNGHSDVVMGLVSVNCES-------------------

-----------------------------------LHNRLRFLQNSLGAVPSPIDCYLCN

RGLKTLHVRMEKHFKNGMAVAQFLESN----PWVEKVIYPGLPS------HPQHELVKRQ

CTGCTG---------------MVTFYIKG----------TLQHAEIFLKNLKLFTLAESL

GGFESLAELPAIMTHASVLKNDRDVLGISDTLIRLSVGLE-DEEDLLEDLDQALKAAHPP

SGSHS-------------------------------------------------------

------------------------------------------------------------

------------------------------------------------------------

------------------------------------------------------------

------------------------------------------------------------

------------------------------------------------------------

------

>Mus_musculus

------------------------------------------------------------

-------------------------------------MQKDASLSGFLPSFQHFATQAIH

VGQEPEQWNSRAVVLPISLATTFKQDFPGQSSG---------------------------

---------FEYSRSGNPTRNCLEK----AVAALDGAKH-------------SLAFASGL

AATITITHLLK-AGDEIICMDEVYGGTNRYFRRVASE-FGLKISFVDCS-----------

KTKLLEAAITP-QTKLVWIETPTNPTLKLADIGACAQIVHKR----GDIILVVDNTFMSA

YFQR---PLALGADICMCSATKYMNGHSDVVMGLVSVNSDD-------------------

-----------------------------------LNSRLRFLQNSLGAVPSPFDCYLCC

RGLKTLQVRMEKHFKNGMAVARFLETN----PRVEKVVYPGLPS------HPQHELAKRQ

CSGCPG---------------MVSFYIKG----------ALQHAKAFLKNLKLFTLAESL

GGYESLAELPAIMTHASVPEKDRATLGINDTLIRLSVGLE-DEQDLLEDLDRALKAAHP-

------------------------------------------------------------

------------------------------------------------------------

------------------------------------------------------------

------------------------------------------------------------

------------------------------------------------------------

------------------------------------------------------------

------

>Anolis_carolinensis

------------------------------------------------------------

------------------------------------MEKGKDKSAGFLPPFKNFATQAIH

AGQDPEQWKSLAVVPPISLATTFKQYAPGQHSG---------------------------

---------FEYSRCGNPTRDCLEK----AVAALDGAKY-------------CLAYASGL

AATLNIAHLLK-AGDNIVCTDDVYGGTNRYFSKMASD-MGLKTVFVDCT-----------

KLESLDAAITP-QTKLVWIETPTNPMLKVIDIQACAKVVHKH----KGVILAVDNTFMSA

YFQR---PLALGADICMYSATKYMNGHSDVVMGLVSVNDDA-------------------

-----------------------------------IHERLRLLQYSLGAVPSPFDCYLCN

RGLKTLPIRMKQHYQNALAVAQFLESD----PRVEKVIFPGLPS------HPQFEVTKRQ

CTGCPG---------------MISFYIKG----------NLTNAATFLKNLKLFALAESL

GGYESLAEHPAIMTHASVPKADRELLGITDTLIRMSIGLE-DSEDLLEDLAQALKAAVPD

FKICN-------------------------------------------------------

------------------------------------------------------------

------------------------------------------------------------

------------------------------------------------------------

------------------------------------------------------------

------------------------------------------------------------

------

>Taeniopygia_guttata

------------------------------------------------------------

-----------------------------------------------------MAAEEPL

CSLQPE--PARGSVCPQRAAVRSARG-----EG---------------------------

---------YEYIRSGNPTRECLEK----AMAALDGGKY-------------CLAYSSGS

AALLNICHLLK-PGDTVISIDDVYGGTNRYFQKVASE-NNLKVIFVDCT-----------

NPKCLEAAITP-ETKLVWLETPTNPTLKVIDIKACADIVRRH----PGVLLAVDNSFMSA

YFQR---PLSLGADICMSSATKYINGHSDVLIGLVSVNRDD-------------------

-----------------------------------LYERLKFLQNALGAVPSPFDCFLCN

RGLKTLHIRMKLHFKNGLAVAKFLECH----PRVEKVIYPGLPS------HPQHEVIKKQ

CTGCPG---------------MVTFYIKG----------NIKNASAFLRKLKVFTLAESL

GGYESLAEHPAIMTHASVPEKEREKLGITDTLIRLSVGLE-DEEDLLADLDQALEAAFA-

------------------------------------------------------------

------------------------------------------------------------

------------------------------------------------------------

------------------------------------------------------------

------------------------------------------------------------

------------------------------------------------------------

------

>Ciona_intestinalis

------------------------------------------------------------

------------------------------------------------------------

------------------------------------------------------------

------------------------------------------------------------

------------------------------------------------------------

---------------ILWVETPTNPTMKVTDIRAVADLEKPS-----ECIIVVDNTFMSS

YFQR---PLLHGADIAYHSATKYMNGHSDVVMGLITTSNDE-------------------

-----------------------------------LYKKLQFLQYATGPVPSAFDCYLVN

RGIKTLALRMEQHQKNAIKVATWLQGH----SGVEKVNYPGLPS------HPQYEIMKKQ

ATGCSG---------------MIAFWLKG----------DLQTAKTFLQSLKVFILAESL

GGYESLAEHPAIMTHASVPAAQREVLGIGDNFIRISVGLE-NVEDLIADLDQAMKLASAG

K-----------------------------------------------------------

------------------------------------------------------------

------------------------------------------------------------

------------------------------------------------------------

------------------------------------------------------------

------------------------------------------------------------

------

>Drosophila_melanogaster

------------------------------------------------------------

--------------------------------------------MSYRVQPSGFATKSIH

SGQSPDQWKSAAVIPPISLSTTFKQDAPGEHRG---------------------------

---------YEYSRSGNPTRNVLET----CFAALDNAKY-------------GLTFSSGL

GATTAVLTMLS-SGDHIIMGDDVYGGTNRLIRQVATR-LGISATFVDPT-----------

KLDLIKSSIKP-ETKLVWIESPTNPLVKVADIEAIAQLVHGVR---EDIVLAVDNTFLTS

YFQR---PLELGADLVCYSLTKYMNGHTDVVMGGITMNSEK-------------------

-----------------------------------LYKSLKFLQNAVGIVPSPFDCYQVN

RSLKTLSLRMEQHQKNALKVAKYLETN----PFVEKVLHPSLPS------HPQHKIALKQ

AYGYSG---------------VFSFYIKG----------ELKHSSAFLKALKVFTLAESL

GGYESLAELPSIMTHASVPAEDRKTLGITDGLVRLSVGLE-DADDLIKDLEQALEIASKA

------------------------------------------------------------

------------------------------------------------------------

------------------------------------------------------------

------------------------------------------------------------

------------------------------------------------------------

------------------------------------------------------------

------

>Caenorhabditis_elegans

------------------------------------------------------------

-----------------------------------------------MATFPHFGTAAIH

VGQEPEQWDMNQVVPPISLSSTYKQDNPGEPKG---------------------------

---------HDYSRAGNPTRDVLQK----NLAALEDAKH-------------CQVFSSGL

AATSAIINLLK-YGDHIVCSDDVYGGTQRYIRRVAVPNHGLEVDSVDLT-----------

DVQNLEKAIKP-NTKMVWFESPSNPLLKVVDIAAVVQTAKKAN---PEIVVVVDNTFMSP

YFQR---PISLGADVVVHSITKYINGHSDVVMGAVITDNDE-------------------

-----------------------------------FQQHLFFMQLAVGAVPSPFDCFLVN

RGLKTLHIRMRAHYENALAVAKYLEAN----DRIESVLYPALPS------HPQHEVHEKQ

TKGMSG---------------MISFYLKG----------ELQESRAFLSALKVFTLAESL

GGYESLAELPSIMTHASVPAETRIVLGITDNLIRISVGIE-DLDDLVADLDQALKIAIPK

V-----------------------------------------------------------

------------------------------------------------------------

------------------------------------------------------------

------------------------------------------------------------

------------------------------------------------------------

------------------------------------------------------------

------

>Monodelphis_domestica

------------------------------------------------------------

------------------------------------MPKGGTPDGGFQPPFPHFATQAIH

AAQEPEQWSGQPVVAPISLSTTFKQTAPGCHAG---------------------------

---------FEYSRSGNPTRNCLEK----AVATLDGAKY-------------CLAFASGL

AATLNITHLLK-AGDHIVCMNDVYGGTNRYFRRVATE-LGLKISFVDCS-----------

KLELLNAAITP-ETKLVWIETPTNPGLKVIDIKGCAQVVHKHG----DVILVVDNTFMSA

YFQSDERPERDGAPSEQKASRILLSRQQPSLSPRRASGSAARAPWWLGLCPEQPQAARPG

PPGGSPEEPQAVRPGPPGGSPEEPQAARPGPPGGSGSARNSLRQRGPGPLVARQKSLRQR

GPGPLVARRKSLRQRGPGPLVARWKSLRQRGPGPLVARWKSLRQ-----RGPGPLVARRK

SLRQRGP------------GPLVARALPGTASG-------SAARAPWWSPLVARALPGTA

SGSAARAPWWSPLVARALPGRASGSAARAPRWLGLCPGPL-PLHRRFSFCWKGQRGERGA

TGWRPSLRPSSPLFCFGAHLGAAARTEREPYQ----------------------------

------------------------------------------------------------

------------------------------------------------------------

------------------------------------------------------------

------------------------------------------------------------

------------------------------------------------------------

------

>Populus_trichocarpa

MAVSSCPCPKLFVASSFECRSDSSSSADQPSQGRSNSARHGTTPFLSGAGMSSLILKFPP

NFVRQLSTKARRNCSNIGVAQIVAASWSNNSAAGAPSAAAAAAAAAAASAIPAAEPAKTL

AGNELEDLSSDLEYKSFLSSDGSIAVHAGERLGRGIVTDAITTPVVNTSAYFFKKTQELI

DFKEKRHASYEYGRYGNPTTQVLED----KISELEGAES-------------TLILASGM

CASTVLLLALVPAGGHLVTTTDCYRKTRIFMETFLPK-MGITVTVIDPA-----------

DVKALESALEINKVSLFFTESPTNPFLRCVDIELVSELCHRKG-----ALVCIDGTFATP

LNQK---ALALGADLILHSATKFIGGHNDVLGGCISGSTKV-------------------

------------------------------------VSEIRNLHHVLGGTLNPNAAYLII

RGMKTLHLRVQQQNSTALRMAKILEAH----PKVRCVYYPGLPS------HPEHHIAKKQ

MTGFGG---------------VVSFEVDG----------DLFITSKFVDALKIPYIAPSF

GGCESIVDQPAIMSYWDLSRSEREKYGIKDNLVRFSFGVE-DFEDLKADILQALETIESA

------------------------------------------------------------

------------------------------------------------------------

------------------------------------------------------------

------------------------------------------------------------

------------------------------------------------------------

------------------------------------------------------------

------

>Arabidopsis_thaliana

------------------------------------------------------------

----------MAHFLETQEPLVFSGKKRNDRDDEDGDALVAKKSA--LAVCD-ADPAAAI

ANIRHEFGEHGGVNMSIEASATFTVMEPDTMRRMFTG-----------------------

-ELGPDNDFYVYSRHFNPTVLNLSR----QMAALEGTQA-------------AYCTSSGM

SAISSVMLQLCSSGGHVVAASTLYGGTHALLSHFLPRTCNITTSFVDIT-----------

DHGAVANAIVEGRTQVLYFESVANPTLTVADIPELSRMAHEKG-----VTVVVDNTFAPM

VLSP----AKLGADVVVHSISKFISGGADIIAGAVCGSENLVK-----------------

----------------------------------EMMDLRGGSLMLLGPTMNAKVAFELS

ERIPHLGLRMREHSHRAQVYAERMRDLG------MKVIYPGLET------HPQHKLFKGM

VNRDYG------------YGGLLSIDMETEEKAN-------KLMAYLQNATQFGFMAVSL

GYYETLMSCSGSSTSSELDPSQKEAAGISPGLVRMSVGYVGTLEQKWTQFEKAFLRM---

------------------------------------------------------------

------------------------------------------------------------

------------------------------------------------------------

------------------------------------------------------------

------------------------------------------------------------

------------------------------------------------------------

------

>Vitis_vinifera

------------------------------------------------------------

----------------MATSVVHAGKKRKGADDED--HLVAKKSASLPAVAKGEDPMAAL

AMARHEFGEHGGVNMSIEASATFTVMEPDTMRRMFSG-----------------------

-ELGPDRDFFIYSRHFNPTVLNLSR----QMAALEGTEA-------------AYCTSSGM

SAISAALLQLCSSGDHIVASRTLYGGTHALLTHFFPRTCNITTSFVDIK-----------

DLDMVDGAIIEGRTKVLYFEAIANPVLSVANVPELCRVAHDKG-----LTVVVDNTFAPM

VMSP----AKLGADVVVHSISKFISGGADIIAGAVCGPAKLVN-----------------

----------------------------------SMMDLHQGALMLLGPTMNPKVAFELS

ERIPHLGLRMKEHCRRALEYATRMKKLG------LKVIYPGLEG------HPDHTVLKSI

ANLDYG------------FGGLLCLDMGTEEKAN-------RLMNLLQNSTQFGFMAVSL

GYYETLMSCSGSSTSSEMNAEEKALAGISPGLVRMSVGYSGTLEQRWSQFEKALSRMQG-

------------------------------------------------------------

------------------------------------------------------------

------------------------------------------------------------

------------------------------------------------------------

------------------------------------------------------------

------------------------------------------------------------

------

>Selaginella_moellendorffii

------------------------------------------------------------

----------------MDSSTKRGGSGSASGFFQD--RYSAKRPR-ASSPPPPLDPEEGL

VSIRHEFGEHGGVNMSIEASSTFTVMEAETMAKMFKG-----------------------

-ELGPDRDFYIYSRHFNPTVLNLGR----QIAAMEGTQA-------------AYCTASGM

SAVASVILQLCDAGDHVVASNRLYGGTYALLRHFLPRTCGIATTFVDVT-----------

DTAAVERAVASRGTKILYVESIANPVLTVADIPALAAIAHARS-----AALVVDNTFAPM

VLSP----ARLGADVVVHSISKFISGQSDVIAGAVCGPAALIR-----------------

----------------------------------DMMGLQHGALMLLGPTMNPTVAFHVS

GRLAHLGLRMREHSSRAMEFAKRMKSMG------LKVIYPGLED------HPQHELLKAL

GNEGYG------------YGGVVAVDLDTDERAN-------RFMHYLQNFTGFGLMAVSL

GYHETLMSCSSSSTSSELNEAERAEAGVGAGLVRLSVGYTGTLEQRWKQLHDAIVALGIA

RKA---------------------------------------------------------

------------------------------------------------------------

------------------------------------------------------------

------------------------------------------------------------

------------------------------------------------------------

------------------------------------------------------------

------

**AATm.fasta**

>Homo_sapiens

-------------------------------MALLHSGRVLPGIAAAFHPG---------

-----------LAAAASARASSWWTHVEMGPPD-----PILGVTEAFKRDTNSKKMNLGV

GAYRDDNGKPYVLPSVRKAEAQIAAK-NLDK--EYLPIGGLAEFCKASAELALGENS---

--EVLKSGRFVTVQTISGTGALRIGASFLQRFFKF--SRDVFLPKPTWGNHTPIFRDAGM

QLQGYRYYDPKTCGFDFTGAVEDISKIPEQS---------VLLLHACAHNPTGVDPRPEQ

WKEIATVVKKRNLFAFFDMAYQGFASGDGDKDAWAVRHFIEQGI------NVCLCQSYAK

NMGLYGERVGAFTMVCKDA-------------DEAKRVESQLKILIRPMYSNPPLNGARI

AAAILNTPDLRKQWLQEVKVMADRIIGMRTQLVSN-LKKEGSTHNWQHITDQIGMFCFTG

LKPEQVERLIKEFSIYMTKDGRISVAGVTSSNVGYLAHAIHQVTK------------

>Pan_troglodytes

-------------------------------MALLHSGRVLPGIAAAFHPG---------

-----------LAAAASARASSWWTHVEMGPPD-----PILGVTEAFKRDTNSKKMNLGV

GAYRDDNGKPYVLPSVRKAEAQIAAK-NLDK--EYLPIGGLAEFCKASAELALGENS---

--EVLKSGRFVTVQTISGTGALRIGASFLQRFFKF--SRDVFLPKPTWGNHTPIFRDAGM

QLQGYRYYDPKTCGFDFTGAVEDISKIPEQS---------VLLLHACAHNPTGVDPRPEQ

WKEIATVVKKRNLFAFFDMAYQGFASGDGDKDAWAVRHFIEQGI------NVCLCQSYAK

NMGLYGERVGAFTMVCKDA-------------DEAKRVESQLKILIRPMYSNPPLNGARI

AAAILNTPDLRKQWLQEVKGMADRIIGMRTQLVSN-LKKEGSTHNWQHITDQIGMFCFTG

LKPEQVERLIKEFSIYMTKDGRISVAGVTSSNVGYLAHAIHQVTK------------

>Mus_musculus

-------------------------------MALLHSSRILSGMAAAFHPG---------

-----------LAAAASARASSWWTHVEMGPPD-----PILGVTEAFKRDTNSKKMNLGV

GAYRDDNGKPYVLPSVRKAEAQIAAK-NLDK--EYLPIGGLAEFCKASAELALGENN---

--EVLKSGRFVTVQTISGTGALRVGASFLQRFFKF--SRDVFLPKPSWGNHTPIFRDAGM

QLQGYRYYDPKTCGFDFSGALEDISKIPEQS---------VLLLHACAHNPTGVDPRPEQ

WKEIASVVKKKNLFAFFDMAYQGFASGDGDKDAWAVRHFIEQGI------NVCLCQSYAK

NMGLYGERVGAFTVVCKDA-------------EEAKRVESQLKILIRPLYSNPPLNGARI

AATILTSPDLRKQWLQEVKGMADRIISMRTQLVSN-LKKEGSSHNWQHITDQIGMFCFTG

LKPEQVERLTKEFSVYMTKDGRISVAGVTSGNVGYLAHAIHQVTK------------

>Monodelphis_domestica

----MHKEQKEGRPEYKHTIGSQEKYVLRILIVKLSPEPISLSIASYIHRSSQSRMR---

----------QSCKSEQKDPYSWWTHVEMGPPD-----PILGVTEAFKRDTNSKKMNLGV

GAYRDDNGKPYVLPSVRKAEAQIAGK-NMDK--EYLPIAGLAEFCKASAELALGENN---

--EVLKSGRYVTVQTISGTGALRVGASFLQRFFKF--SRDVYLPKPSWGNHTPIFRDAGM

QLNGYRYYDPKTCGFDFTGAMEDISKIPEQS---------VILLHACAHNPTGVDPRPEQ

WKEMASLVKQKKLFAFFDMAYQGFASGDGNKDAWAVRHFIEQGI------NVSLCQSYAK

NMGLYGERVGAFTMVCKDA-------------DEAKRVESQLKILIRPMYSNPPLNGARI

ASTILNSPDLRSQWLQEVKGMADRIISMRTQLVSN-LKKEGSSHNWQHITDQIGMFCFTG

LKPEQVERLTKEFSIYMTKDGRISVAGVTSGNVGYLAHAIHQVTK------------

>Anolis_carolinensis

-------------------------------MALLFPVRL-----AALPPR---------

------------LASCAARASSWWAQVEMGPPD-----PILGVTEAFKRDTSAKKMNLGV

GAYRDDNGKPYVLNCVRKAEAQIAAK-KMDK--EYLPIAGLAEFTKASAELALGESN---

--EVIQSGRYVTVQTISGTGSLRVGANFLQRFFKS--SRDVYLPKPSWGNHTPIFRDAGM

QLQSYRYYDPKTCGFDFTGALEDISKIPEKS---------IILLHACAHNPTGVDPRPEQ

WKEMAALVKKRNLFAFFDMAYQGFASGDINRDAWAVRHFIEQGI------NIVLSQSYAK

NMGLYGERVGAFTVVCQDA-------------EEAKRVESQLKILIRPMYSNPPVNGARI

ASTILNSPDLRKEWLTEVKGMADRIIGMRTQLVAN-LKKEGSSHNWQHITDQIGMFCFTG

LKPEQVERLTKEFSVYMTKDGRISVAGVTSGNVAHLAHAIHQVSK------------

>Xenopus_tropicalis

-------------------------------MALLKSRLLVG--VARCQPC---------

------------LAAVQGRASSWWSHVEMGPPD-----PILGVTEAFKRDTNSKKMNLGV

GAYRDDNGKPYVLSSVRKAEAQLASK-NLDK--EYLPIGGLAEFARASAQLALGENC---

--DAIQSGRFITVQTISGTGSLRVGANFLQRFYKY--SRDVYLPKPSWGNHTPIFRDAGL

EVKGYRYYDPKTCGFDFTGALDDISKIPEQS---------IILFHACAHNPTGVDPKQEQ

WKELAALIKSRRLFPFFDMAYQGFASGDTNRDAWAVRHFIQEGI------NVVLSQSYAK

NMGLYGERVGAFTVVCSDA-------------EEAKRVESQLKILIRPMYSNPPLNGARI

AAAILTQPDLRKEWLQEVKGMANRIISMREQLVSN-LKKEGSIHNWQHISDQIGMFCFTG

LRPEQVERLIKEFSIYMTKDGRISVAGVTSANNGYLAHAIHQVTK------------

>Danio_rerio

-------------------------------MALLKTTKFISS-VGSLTPS---------

------------LASLPIRASSWWTEVQMGPPD-----PILGVTEAFKRDTNSKKMNLGV

GAYRDDNGKPYVLNCVRKAESLIASK-ALDK--EYLGIVGLGDFNKACAELALGQDS---

--DVLKSKRSITVQTISGTGSLRVGANFLSRFHTV--ARDVYLPKPSWGNHTPIFRDAGM

QLKAYRYYDPATCGFDFTGALDDISKIPQNS---------VILLHACAHNPTGVDPRPEQ

WKELSAVIKERKLLPFFDMAYQGFASGDIDRDAWAVRYFIEQGH------NILLSQSFAK

NMGLYGERVGGFTVVCGDA-------------EEAKRVESQLKILIRPIYSNPPMNGARI

ASTILTTPELRSTWLEEVKGMADRIIRMREMLVTN-LKKEGSTHNWQHVTDQIGMFCFTG

LKPEQVERLTKDFSVYMTKDGRISVAGVTSGNVEYLAHAIHQVTK------------

>Ciona_intestinalis

-------------------------------MSLTINKNICS-LLQKCSVG---------

-------------HRSLNRTGSWWSNVEMGPPD-----PILGVTEAFKRDTNPKKMNLGV

GAYRDDQGKPYVLPTVKKAELAIQG---LDK--EYLGITGLPAFTNAAAELAFGAGN---

--TVLTDKRNVTVQGISGTGSLRIGANFLNKFFTS--NKAIWLPTPSWGNHVPIFKHAGL

DVEWYRYYKPSTCGFDAEGAMEDLNKIPENS---------IVLFHACAHNPTGVDPKPEN

WKEMSSICKKRNLLPFFDMAYQGFASGDINKDASAMRLFVAEGH------NVILAQSFAK

NMGLYGERAGAFTVVCADQ-------------EEAARVESQIKILIRPMYSNPPCNGARI

ASTVLTTPELREQWLVEVKGMADRIISMRQQLVDN-LKKEGSTRDWSHITDQIGMFCYTG

LNPDQVGDLTKNHSVYLTKDGRISVAGVASGNVGYLAHAIHQVTK------------

>Caenorhabditis_elegans

---------------------------------MSVSKKLFS------------------

---------------TAVRGKSWWSHVEMGPPD-----AILGVTEAFKADSNPKKINLGV

GAYRDDQGKPFVLPSVKEAERQVIAA-NLDK--EYAGIVGLPEFTKLSAQLALGENS---

--DVIKNKRIFTTQSISGTGALRIGSEFLSKYAK---TKVIYQPTPTWGNHVPIFKFAGV

DVKQYRYYDKSTCGFDETGALADIAQIPEGS---------TILLHACAHNPTGVDPSRDQ

WKKISDIVKKRNLFVFFDMAYQGFASGDVDNDAFAVRYFVEQGH------NIVLSQSFAK

NMGLYGERVGAFSVVTSDA-------------DEAARVASQVKILIRPLYSNPPVHGARI

ASRILADPALNKQWLGDVKLMADRIITMRTTLKDL-LAKEGSTRNWEHITNQIGMFCFTG

INPQQVEKLIKEHSVYLTKDGRISVAGISSNNVAYLAHALHQVTK------------

>Drosophila_melanogaster

-------------------------------MSQICKRGLLIS--NRLAPA---------

---------------ALRCKSTWFSEVQMGPPD-----AILGVTEAFKKDTNPKKINLGA

GAYRDDNTQPFVLPSVREAEKRVVSR-SLDK--EYATIIGIPEFYNKAIELALGKGS---

--KRLAAKHNVTAQSISGTGALRIGAAFLAKFWQG--NREIYIPSPSWGNHVAIFEHAGL

PVNRYRYYDKDTCALDFGGLIEDLKKIPEKS---------IVLLHACAHNPTGVDPTLEQ

WREISALVKKRNLYPFIDMAYQGFATGDIDRDAQAVRTFEADGH------DFCLAQSFAK

NMGLYGERAGAFTVLCSDE-------------EEAARVMSQVKILIRGLYSNPPVHGARI

AAEILNNEDLRAQWLKDVKLMADRIIDVRTKLKDN-LIKLGSSQNWDHIVNQIGMFCFTG

LKPEQVQKLIKDHSVYLTNDGRVSMAGVTSKNVEYLAESIHKVTK------------

>Ustilago_maydis_521

------------------------------MMLTAATIRTAVR--ASQAPLLGAMS----

-------------LPAVRQFKSTWAHVKAGPPD-----PILGVTEAFKRDQDPRKINLGV

GAYRDENGKPYVLPSVRQAEELVITA-KGDK--EYLPITGLADFTKNAAVLAYGKDS---

--APIKENRIAITQSISGTGALRIGGAFLQRHYPE--AKTIYLPTPSWGNHTPIFRDSGL

EVKQYRYYDKKTVGLDFKGMIEDIKAAPAGS---------IVLLHACAHNPTGVDPTVEQ

WKEISNVVKEKGHFPFFDMAYQGFASGDTDKDAFAVRHFVAEGH------QICLSQSFAK

NMGLYGERVGAFSIVCADP-------------DERARVDSQIKIIVRPMYSNPPMHGAKI

AGTILADQQLYQQWLGEVKGMADRINGMRSTLKDLLVQDFNSKLNWDHITNQIGMFAFLG

ISPEQVAKLVNEHHVYLTGDGRISVAGITDHNVKHLAESLHKVTS------------

>Puccinia_graminis

---------------------------------MASLARSLAR--NSSPSAL--FG----

-------------RQHVLRSITTWSNVPAGPPD-----PILGVTEAFKACTSPLKMNLGV

GAYRDQDGKPFVLPSVRQAEAAIVAA-KYDK--EYLGITGFPEFTKRAAILAYGSDS---

--APLKEGRIAITQSISGTGALRIGGAFLQRFYPH--SKSIYLPTPTWGNHIPIFKDSGL

EVKQYRYYDKKTVGLDASGMLEDIKNAPDRS---------IILLHACAHNPTGVDATQEQ

WKQIAQVIKEKGHFSFFDMAYQGFASGDVDRDAFAPRYFVSQGL------DIVLSQSFAK

NMGLYGERVGAFSVVCSSP-------------EEKSKVESQVKILVRPMYSNPPVHGARI

AGTILSDPALYKQWLGEVKLMADRIIGMRTALYDTLVNELGSQRNWDHIKSQIGMFCFAG

ISPEQVEQMTKNHHVYMTKDGRISMAGVTPHNVKNLAKALHDVTK------------

>Gibberella_zeae_PH-1

---------------------------------MLSTLRVASRRAARPAQYN--------

--------------LAAIRAASNWANVPQGPPD-----AILGITEAFKADKFDKKINLGV

GAYRDDAGKPYVLPSVREAEMKVVES-KLNK--EYAGITGVPEFPPAAAKLAYGANS---

--PAL--DRITITQTISGTGALRVGAAFLAKFFPG--EKKIYIPQPSWANHKAVFNHAGL

EVEQYRYYDKKTIGLDFEGLIADVKSAPNGS---------VFLFHACAHNPTGVDPTQEQ

WKQISDVVKEKGHFAFFDMAYQGFASGDTDKDAFAVRYFVEQGH------NIALCQSFAK

NMGLYGERIGAFSMVCADA-------------DEKKRVDSQLKILIRPLYSNPPIHGARI

AAEILNSPTLYKQWLGEVKEMADRIITMRALLKDN-LEKLGSKHDWSHITSQIGMFAYTG

LTSEEMTRLAEEFSVYATKDGRISVAGITSENVGRLAEAIYKVKG------------

>Neurospora_crassa_OR74A

---------------------------------MLSSVRLVTRRAAVRSAFASPGR----

-------------SLLAVRAASFWANVPQGPP--------VSITEAFKADTFDKKINLGV

GAYRDDKGKPYVLPSVRKAEDKVIHS-RMNK--EYAGITGVPEFTKAAAVLAYGKDS---

--SAL--DRLVITQSISGTGALRIGGEFLNRFFPG--AKKIYIPTPSWANHGAVFRDSGL

EVAQYAYYDKNTIGLDFEGLIRDLKAAPNSS---------IFLFHACAHNPTGVDPTPEQ

WKEIEAVVKDKGHYSFFDMAYQGFASGDIHKDAFAVRYFVEQGH------NICLAQSFAK

NMGLYGERVGAFSIVCADA-------------DEKKRVDSQVKILVRPLYSNPPIHGARI

AAEILNTPELYEQWLTEVKEMADRIIKMRALLKEN-LEKLGSKHDWSHITSQIGMFAYTG

LTPEQMEKLAKEHSVYATKDGRISVAGITSENVGRLAEAIFKVKG------------

>Pyrenophora_tritici-repentis

---------------------------------MLASSRTVLRQAANRQLSLKT------

----------------SVRAVSVWSQIPQGPPLTLPSQAILGITEAFKADSNPKKINLGV

GAYRDDKGKPYVLPSVRQAEKKILDS-SLDK--EYAGITGVPNFTKAALRLAYGEDS---

--TPLKNDCIAVTQSISGTGALRIGGAFFERHYPG--AKTIYIPTPSWANHNAVFKDSGL

KVEKYRYYNKDTIGLDFDGMVADIKAMPKNS---------IVLLHACAHNPTGVDPTEEQ

WVKIAEAVKAGDHFPFFDMAYQGFASGDTSKDAFALRHFIKEGL------RPVLAQSFAK

NMGLYGERVGAFSIVCESA-------------EEKKRVDSQIKILVRPLYSNPPVHGARI

ASEILNDSSLNQQWLGEVKGMADRIITMRALLKEN-LEKLGSKHDWSHITSQIGMFAYTG

LTAEQMDKLAKEHSVYATKDGRISVAGITTENVGRLAEAIYKVKG------------

>Aspergillus_niger

---------------------------------MLSTLRVASRKAATRDASVRTV-----

--------------IVGARHASAWSNVPQGPP-----DAILGITEAYKADTFKEKINLGV

GAYRDDQGKPYVLPSVRAAEDKVVAT-RLDK--EYAGITGIPAFTKAAAELAYGADS---

--AVLKEDRLVITQTISGTGALRIGGAFLQRFYPH--AKKVYLPTPSWANHAAVFKDAGL

EVDKYRYYNKDTIGLDFDGLLADIKAAPENS---------IILLHACAHNPTGVDPTQEQ

WRQISDVMKQKGHFAFFDMAYQGFASGNADQDAFAPRHFVKEGH------NIALCQSFAK

NMGLYGERVGAFSLVCESA-------------EEKKRVDSQIKILIRPFYSNPPIHGARI

ASTIMNDAKLNEQWLGEVKGMADRIIEMRALLRKN-LEELGSKHDWSHITSQIGMFAYTG

LKPEQMDALAKEHSVYATKDGRISVAGITSGNVKRLAESIYKITG------------

>Schizosaccharomyces_pombe

-------------------------------MLARNLRCLHPNTFASLKTNVSYHGV---

----------KCLASQSKRGFKVWADVPMGPPD-----PIFGITEAYKKDGDVKKMNLGA

GTYRDDAGKPYVLPSVRQAETELLSQ-KLDK--EYAPITGIPSFRVQATKLAYGDVY---

--ESIK-DRLVSAQSISGTGALCIAANFLASFYP---SKTIYVSDPTWGNHKNVFSRAGL

TVKSYKYYDPATRGLDIKGMLSDLTSAPDGS---------IILLHACAHNPTGVDPTKAQ

WDDILKTMQKKNHFALLDMAYQGFASGDFARDAYATRLFASSNV------PMLLCQSFAK

NMGLYGERAGCFSILANDA-------------EEAARIESQTKILIRALYSNPPVNGARI

ANHILSNPALREQWAGEVVGMSERLKSMRKALRNILEKDLKNKHSWKHITDQIGMFCYTG

LNPQQVDVLAKQYHIYLTKNGRISISGLNTSNVRYFAEAINAVTSN-----------

>Selaginella_moellendorffii

---------------------------------MSTAAAPASSVPAGA------------

-------------------RSGWWEAVQPAARD-----PILGVTEAFLADSDPKKVNVGV

GAYRNDEGKPVVLECVRKAEQIIAG--KQNM--EYLPMGGLVKFNDLSVKLAYGDTA---

--PVLEEKRVAAVQTLSGTGACRLFADFQKRFKP---DSRIYIPVPTWANHHNIWRDARV

EAHTFRYYKPSTRGLDFEGLMEDLKKAPEGS---------FVLLHACAHNPTGVDPTADQ

WKEMSQLFKSNRLFPFFDMAYQGFASGDTVRDAQAIRIFMEDGH------QLACAQSFAK

NMGLYGQRVGCLSVVCDNS-------------QQAVNVKSQLQQIARPMYSNPPLHGAQI

VTTVLSDPELKEQWYKEVKVMADRIIGMREALKSNLEKLGSSL-PWKHITEQIGMFCYSG

LTEDQVDRLTKEFHIYMTRNGRISMAGVTTGNVEYLANAIHEVTK------------

>Vitis_vinifera

-------------------------------MAFRTGISRRMMIRSGFGLRS--------

-------------------MSSWWGSVEPAAKD-----PILGVTEAFLADPSPDKVNVGV

GAYRDDNGKPVVLECVREAERRIAG--KLNM--EYLPMGGSVNMVEETLKLAYGENS---

--EFIKDKRIAAVQALSGTGACRLFADFQKRFRP---DSQIYIPVPTWANHHNIWRDAQV

PQRTFHYYHPETKGLDFASLMDDVKNAPNGS---------FFLLHACAHNPTGVDPSEEQ

WKEISYQFKVKGHFAFFDMAYQGFASGDPERDAKSIKIFLEDGH------LIGIAQSYAK

NMGLYGQRVGCLSLLCEDE-------------KQAVAVKSQLQQIARPMYSNPPVQGALI

VSTILGDPELKKLWLKEVKVMADRIIGMRTALRENLEKLGSPL-SWEHITNQIGMFCYSG

LTPEQVDRMTNEFHIYMTRNGRISMAGITTGNVSYLANAIHEVSKSA----------

>Physcomitrella_patens

------------------------------------------------------------

--MAP----------------SVFEHVQQAPED-----PILGVTVAYNKDPSPLKVNLGV

GAYRTEEGKPLVLNVVRRAEQQLVADRSRNK--EYQPITGISQFNKLSAKLILGANS---

--PAIAENRVATVQALSGTGALRVGAEFISRHY---AKPIIFLPNPTWGNHNKIFPLGGV

PQKPYRYYDPKTRGLDYEGMLEDLKAAPDGA---------VILLHACAHNPTGVDPTEEQ

WEGIRQVIRSKHQLPFFDCAYQGFASGSLDKDAHAVRLFVADGG------ECFVAQSYAK

NMGLYGERVGALSVVCTNA-------------AVASRVDSQLKLVIRPMYSSPPAHGAAI

AATILADGRLFQEWTVELKGMADRIISMRQQLYDALQARGTPG-DWTHVLKQIGMFTFTG

LNKSQVEFMTRQYHIYMTSDGRISMAGLSSKTVPHLADAIHAAVVGAQRKS------

>Populus_trichocarpa

-------------------------MNPELTSPSSSSDRRISVLARHLVGVE--------

--MDPQNDSISAFPTSGSDSNSVFSHVVRGPED-----PILGVTVAYNKDPSPVKLNLGV

GAYRTEEGKPLVLNVVRKAEQLLVNDRSRVK--EYLPITGLAEFNKLSAKLMFGANC---

--PAIQENRVTTVQCLSGTGSLRVGAEFLAKHH---HQRTIYIPQPTWGNHPKIFTLAGL

SVKTYRYYDPATRGLNFQGLVEDLNSAPSGA---------IVLLHACAHNPTGVDPTSQQ

WEQIRKLMRSKGLMPFFDSAYQGFASGSLDADAQPVRMFVADGG------ELLLAQSYAK

NMGLYGERIGALSIVCKTA-------------DVAGRVESQLKLVIRPMYSNPPIHGASI

VAAILKDRDLYNEWTIELKAMADRIISMRQKLFEALHARGTPG-DWSHIVKQIGMFTFTG

LNSKQVAFMTKEYHIYMTSDGRISMAGLSSKTVPHLADAMHAAVKRVV---------

>Chlamydomonas_reinhardtii

--------------------------------MCDSPNARLAVLARQLCAGG--------

--PGG----------------STWEHVQQAPPD-----PIIGVNDSYRADTDPRKLNLGV

GAYRTEEGKPYVLRAVREAEAALAADPAANK--EYLPIAGLPEFNRLSRELALGPSH---

--PAIRDGRVATVQALSGTGALRVGAEFLAQHLPPGQPRVVYLPNPTWGNHKTIFGRAGM

QVREYRYFDAKTRGLDFAGMCADLSAAPPGA---------VLLLHACAHNPTGVDPSPEQ

WRQLLALTQERKLLPFFDSAYQGFASGDLDADAASVRLFASAPGPQ----EMVLAQSFAK

NMGLYGERAGALSVVCKSK-------------EVAGRVESQLKLVIRPMYSNPPMHGAAI

AARVMADPRLNALWKEELAGMAHRIKAMRQALYGQLVARQLPG-DWSFVLKQIGMFSYTG

LSKAQCEVLTRKWHIHLTMDGRISMAGLSAASCPYLAEAIADVVTNGGSA-------

>Zea_mays

-------------------------MATAAAFSVSSPAASAVAARSKVFGGVNQARTRTG

CRVGITRKNFGRVMMALAVDVSRFEGVPMAPPD-----PILGVSEAFKADKSELKLNLGV

GAYRTEELQPYVLNVVKKAENLMLE-KGENK--EYLPIEGLAAFNKATAELLLGADN---

--PVINQGLVATLQSLSGTGSLRLAAAFIQRYFP---EAKVLISSPTWGNHKNIFNDARV

PWSEYRYYDPKTVGLDFEGIIADIEAAPEGS---------FVLLHGCAHNPTGIDPTPEQ

WEKIADVIQEKKHMPFFDVAYQGFASGSLDEDAFSVRLFVKRGM------EVFVAQSYSK

NLGLYSERVGAINVVCSAP-------------EVADRVNSQLKRLARPMYSNPPIHGAKI

VANVVGDPTMFGEWKQEMELMAGRIKNVRQKLYDSLSAKDKSGKDWSFILRQIGMFSYTG

LNKAQSDNMTDKWHIYMTKDGRISLAGLSLAKCDYLADAIIDSFHNVN---------

>Oryza_sativa

-------------------------MASAPFAVSSSPAASAVAARSKVLRGG-RSEARTG

CRLGITRKNFGRVMMALAVDVSRFEGVPMAPPD-----PILGVSEAFKADQNDLKLNLGV

GAYRTEELQPYVLNVVKKAETLMLE-KGENK--EYLPIEGLAAFNKATAELLFGADN---

--PVLKQGRVATLQSLSGTGSLRLAAAFIQRYFP---EAKVLISSPTWGNHKNIFNDAKV

PWSEYRYYDPKTVGLDFEGMIADIQAAPDGS---------FVLLHGCAHNPTGIDPTPEQ

WEKIADVIQEKKHMPFFDVAYQGFASGSLDEDASSVRLFVQRGL------EVFVAQSYSK

NLGLYAERIGAINVVCSTP-------------EVANRVKSQLKRLARPMYSNPPIHGARI

VANVVGDPTMFGEWKQEMEEMAGRIKNVRQKLYDSLSAKDDSGKDWSFILRQIGMFSYTG

LNKTQSDNMTDKWHIYMTKDGRISLAGLSLAKCEYLADAIIDSFHNVS---------

>Arabidopsis_thaliana

-------------------------MASLMLSLGSTSLLPREINKDKLKLGT------SA

SNPFLKAKSFSRVTMTVAVKPSRFEGITMAPPD-----PILGVSEAFKADTNGMKLNLGV

GAYRTEELQPYVLNVVKKAENLMLE-RGDNK--EYLPIEGLAAFNKATAELLFGAGH---

--PVIKEQRVATIQGLSGTGSLRLAAALIERYFP---GAKVVISSPTWGNHKNIFNDAKV

PWSEYRYYDPKTIGLDFEGMIADIKEAPEGS---------FILLHGCAHNPTGIDPTPEQ

WVKIADVIQEKNHIPFFDVAYQGFASGSLDEDAASVRLFAERGM------EFFVAQSYSK

NLGLYAERIGAINVVCSSA-------------DAATRVKSQLKRIARPMYSNPPVHGARI

VANVVGDVTMFSEWKAEMEMMAGRIKTVRQELYDSLVSKDKSGKDWSFILKQIGMFSFTG

LNKAQSDNMTDKWHVYMTKDGRISLAGLSLAKCEYLADAIIDSYHNALLYWETPIEL

>Sclerotinia_sclerotiorum_1980_

-----------------------------------MSELSATSFPEN-------------

-------------------------AVPKAPED-----PLFGLMAAYRADTFDKKVDLGI

GAYRTNDAKPWVLPVVKKADEILRNDPALNH--EYLPIAGLNTFTSAAAKLMLGADS---

--PALADKRAYSIQAISGTGAVHLGALFFKKFYP--NSPTVYLSNPTWVNHHQIFSNVHL

PVATYPYFSKSTKGLDFDGMKSTIQNAPDKS---------VILLHACAHNPTGVDPTKDQ

WKELAVLLKQKQHFPFFDCAYQGFASGDLANDAWAIRYFIEQGF------ELCIAQSFAK

NLGLYSERAGCFHFVTGAAPDA---------EQTIGRIASQLTLLQRSEISTPPAYGARI

ASTILNDEALFKEWEENLKEMSGRIMTMRKELRSKLEEMGTPG-KWNHITDQIGMFSFTG

LTEKQVLELRSEAHVYMTNNGRISMAGLNTNNIGYFAQAVDKVVRKTQQETANL---

>Saccharomyces_cerevisiae_S288c

--------------------------------MLRTRLTNCSLWRPYYTSS---------

-----------------------LSRVPRAPPD-----KVLGLSEHFKKVKNVNKIDLTV

GIYKDGWGKVTTFPSVAKAQKLIESHLELNKNLSYLPITGSKEFQENVMKFLFKESCPQF

GPFYLAHDRISFVQTLSGTGALAVAAKFLALFIS----RDIWIPDPSWANHKNIFQNNGF

EN-IYRYSYYKDGQIDIDGWIEQLKTFAYNNQQENNKNPPCIILHACCHNPTGLDPTKEQ

WEKIIDTIYELKMVPIVDMAYQGLESGNLLKDAYLLRLCLNVNKYPNWSNGIFLCQSFAK

NMGLYGERVGSLSVITPATANNGKFNPLQQKNSLQQNIDSQLKKIVRGMYSSPPGYGSRV

VNVVLSDFKLKQQWFKDVDFMVQRLHHVRQEMFDR-----LGWPDLVNFAQQHGMFYYTR

FSPKQVEILRNNYFVYLTGDGRLSLSGVNDSNVDYLCESLEAVSKMDKLA-------

**AATc.fasta**

>Xenopus_tropicalis

------------------------------------------------------------

-MA----SIFTS--VPQAPPVAVFKLTADFRADPDTRKVNLGVGAYRTDDSQPWVLPVVK

KVEQMIAKDSSLNHEYLPILGLPEFRSSASRIALGDDSPAFKENRVGGVQSLGGTGALRI

GAEFLRRWYNGNNN---TATPIYISSPSW---ENHNAVFSDAGFKDIRAYSYWDAAKRGL

DLEGFLRDLENAPEFSIFVLHACAHNPTGTDPTPDEWKQIADVMKRRSLFPFFDSAYQGF

ASGSLDKDAWAVR---FFVSQG-FELFCAQSFSKNFGLYNERVGNLTVVGKDCDNVA---

-RVLSQMEKIVRTTWSNPPSQGARIVATTLNTPELFDEWRDNVKTMAERVLLMRAELKSR

LE-ALKTP-GTWNHIVNQIGMFSYTGLNPKQVEYLIKEKHIYLMASGRINMCGLTTKNLD

YVAQSIYEAATKIQ-----------

>Anolis_carolinensis

------------------------------------------------------------

-MAPGGKSVFAA--VPQALPVAVFQLTADFRADADPRKVNLGVGAYRTDEGQPWVLPVVR

KVETMITKDTSLNHEYLPILGLPEFRANSSRIALGDDSPAIKENRVGGVQSLGGTGALRI

GAEFLRRWYNGTNN---TATPVYISSPTW---ENHNSVFTDAGFKDIRSYRYWDAAKRGL

DIQGFLEDLEKAPEFSIFILHACAHNPTGTDPTQEQWKQIAAVMKRRFLFPFFDSAYQGF

ASGSLDRDAWAVR---YFVSEG-FELFCAQSFSKNFGLYNERVGNLTVVAKDGENVQ---

-RVLSQMEKIVRTTWSNPPSQGARIVATTLTTPELFAEWKGNVKTMADRVLLMRAELRSR

LE-ALHTP-GTWNHITEQIGMFSFTGLNPKQVLFLIKEKHIYLMASGRINMCGLTAKNLD

YVAASIHEAVTKIQ-----------

>Homo_sapiens

------------------------------------------------------------

-MAP--PSVFAE--VPQAQPVLVFKLTADFREDPDPRKVNLGVGAYRTDDCHPWVLPVVK

KVEQKIANDNSLNHEYLPILGLAEFRSCASRLALGDDSPALKEKRVGGVQSLGGTGALRI

GADFLARWYNGTNN---KNTPVYVSSPTW---ENHNAVFSAAGFKDIRSYRYWDAEKRGL

DLQGFLNDLENAPEFSIVVLHACAHNPTGIDPTPEQWKQIASVMKHRFLFPFFDSAYQGF

ASGNLERDAWAIR---YFVSEG-FEFFCAQSFSKNFGLYNERVGNLTVVGKEPESIL---

-QVLSQMEKIVRITWSNPPAQGARIVASTLSNPELFEEWTGNVKTMADRILTMRSELRAR

LE-ALKTP-GTWNHITDQIGMFSFTGLNPKQVEYLVNEKHIYLLPSGRINVSGLTTKNLD

YVATSIHEAVTKIQ-----------

>Pan_troglodytes

------------------------------------------------------------

-MAP--PSVFAE--VPQAQPVLVFKLTADFREDPDPRKVNLGVGAYRTDDCHPWVLPVVK

KVEQKIANDNSLNHEYLPILGLAEFRSCASRLALGDDSPALKEKRVGGVQSLGGTGALRI

GADFLARWYNGTNN---KNTPVYVSSPTW---ENHNAVFSAAGFKDIRSYRYWDAEKRGL

DLQGFLNDLENAPEFSIVVLHACAHNPTGIDPTPEQWKQIASVMKHRFLFPFFDSAYQGF

ASGNLERDAWAIR---YFVSEG-FEFFCAQSFSKNFGLYNERVGNLTVVGKEPESIL---

-QVLSQMEKIVRITWSNPPAQGARIVASTLSNPELFEEWTGNVKTMADRILSMRSELRAR

LE-ALKTP-GTWNHITDQIGMFSFTGLNPKQVEYLVNEKHIYLLPSGRINVSGLTTKNLD

YVATSIHEAVTKIQ-----------

>Mus_musculus

------------------------------------------------------------

-MAP--PSVFAQ--VPQAPPVLVFKLTADFRDDPDPRKVNLGVGAYRTDESQPWVLPVVR

KVEQKIANDNSLNHEYLPILGLAEFRSCASRLVLGDNSLAIRENRVGGVQSLGGTGALRI

GADFLGRWYNGTDN---KNTPIYVSSPTW---ENHNAVFSAAGFKDIRPYCYWDAEKRGL

DLQGFLNDLENAPEFSIFVLHACAHNPTGTDPTPEQWKQIAAVMQRRFLFPFFDSAYQGF

ASGDLEKDAWAIR---YFVSEG-FELFCAQSFSKNFGLYNERVGNLTVVGKESDSVL---

-RVLSQMEKIVRITWSNPPAQGARIVAATLSDPELFKEWKGNVKTMADRILTMRSELRAR

LE-ALKTP-GTWSHITEQIGMFSFTGLNPKQVEYLVNEKHIYLLPSGRINMCGLTTKNLD

YVATSIHEAVTKIQ-----------

>Monodelphis_domestica

------------------------MRERLVSPVRDFRIDALGAGAGPRPGGVRALAAALG

LTMP--VSVFAD--VPQAQPVLVFKLTADFRKDPDPRKVNLGVGAYRTDESQPWVLPVVR

KVQQQIAANESINHEYLPILGLPEFRSNASRIALGDDSPAIKENRIGSVQGLGGTGALRI

GAEFLRRWYNGTKN---AATPVYVSSPTW---ENHNGVFSAAGFTDIRSYHYWDASKRGL

DLQGMLQDMENAPEFSIFVLHACAHNPTGTDPTPEQWEKIASVMKRRFLFPFFDSAYQGF

ASGDLDKDAWAVR---YFVNEG-FELFCAQSFSKNFGLYNERVGNLIVVGKDGDNVL---

-RVLSQMEKIVRVVWSNPPAQGARIVATTLSSPELFSEWRDNVKTMADRILLMRAELRSR

LE-ALGTP-GTWSHITDQIGMFSFTGLSAKQVEYLINEKHIYLLPSGRINMCGLTTKNLD

YVATSIHEAVTKIQ-----------

>Danio_rerio

------------------------------------------------------------

------MSIFGE--VPQAAPVAVFKLTADFREDQDQKKVNLGVGAYRTDECQPWVLPVVR

KVEKMIADDHSLNHEYLPILGLPEFRSSASKIALGEDSPAIKENRVGAVQCLGGTGALKI

GAEFLRRWYNGTDN---TKTPVYVSAPTW---ENHNAVFSNAGFEDIRPYKYWDPVKRGL

DLAGFLGDMESAPDHSIFVLHACAHNPTGTDPTQDQWKQIAEVMKRKNLFAFFDSAYQGF

ASGDLEKDAWAVR---YFVSQG-FELFCAQSFSKNFGLYNERVGNLTVVAKDQDNVN---

-RVLSQMEKIVRITWSNPPSQGARLVAITLNTPELFAEWKANVKTMADRVLLMRAQLKEK

LK-ALGTP-GTWEHITEQIGMFSFTGLNPKQVEYMIKEKHIYLMASGRINMCGLTSKNID

YVAESIHEAVTKVQ-----------

>Taeniopygia_guttata

------------------------------------------------------------

----------------MAEVFVVY--------------VTLSELLFET------------

----------SFPSPSNPFPDITGLRADSHKLQK----------------------LLEI

GGRFLT---ETATH---RQAPTPIPGKAWGQPRNHNSVFMDAGFPDIITYHYWDAAKRGL

DLQGLLGDMEHAPEFSIFILHACAHNPTGTDPTPEQWKQIAAVMKRRSLFPFFDSAYQGF

ASGSLEKDAWAVR---YFVSEG-FELFCAQSFSKNFGLYNERVGNLTVVGKDADNVQ---

-RVLSQMEKIVRTTWSNPPSQGARIVATTLSSPQLFDEWKGNVKTMADRVLLMRSELRSR

LE-ALGTP-GTWNHITEQIGMFSFTGLNPKQVEYMVKEKHIYLMASGRINMCGLTTKNLD

YVAKSIHEAVTKIQ-----------

>Ciona_intestinalis

------------------------------------------------------------

-MSS--PSLFAD--VELAAPVVVFQLTADYNADNADKKINLGVGAYRTDEGEPWVLPVVR

SVEAQMAIDPALNHEYLPILGLPSFRELATQLILGKDSRAILENRAGGVQSISGTGALRL

AAEFLYRYYNKRE----KSTPVYVSSPTWG---NQTAVFKNAGFTDMRTYRYWDAEDRCL

DYKGMLEDMLNAPEYSIFIFHGCAHNPTGVDPTHEQWQGIAKACKERNIFPVLDCAYQGF

ASGDPDVDAYSAR---MFVDLG-FEVLICQSFAKNFGLYNERVGNLTMVMHDAPTLS---

-RCKSQVELIIRAMYSNPPHHGARVVASTLANPAFKQEWLDNLHTMSSRIKEMRQLLHSK

LR-AKGTP-GNWDHIINQIGMFSFTGLNASQVQYLKKR-HIYLLSSGRINMCGLTTSNME

YFVDNVHDAVTNVKDGKL-------

>Drosophila_melanogaster

------------------------------------------------------------

------MSIYAD--VPKGPAIEVFALTQAFKDDSNPNKVNLSVGAYRTDAGVPWVLPVVR

KTEISIASDEQVNHEYLPVTGLETFTNAATELVLGADSPAIKENRAFGVQTISGTGALRV

AADFLH-----TQL---NRNVVYYSNPTWE---NHHKIFCDAGFTTVKSYRYWDQNKREL

DFKNMLADLNDAPPGAVIILHACAHNPTGIDPTQEQWTELADLMEKKKLFPLFDSAYQGF

ASGDPDRDAWAAR---YFVQRG-FELFICQSFAKNFGLYCERTGNLAVVQKNGATKA---

-AVHSQLTLLIRGQYSNPPAYGARIVSKVLNTPELRKEWMASIQAMSSRIREMRTALRDK

LV-ALGTP-GTWDHIVNQIGMFSYTGLNESHVRVLIDQYHIYLLKTGRISMSGLNKGNVE

YVAKAIHAAVTGSGESASCPCENKL

>Caenorhabditis_elegans

------------------------------------------------------------

------MSFFDG--IPVAPPIEVFHKNKMYLDETAPVKVNLTIGAYRTEEGQPWVLPVVH

ETEVEIANDTSLNHEYLPVLGHEGFRKAATELVLGAESPAIKEERSFGVQCLSGTGALRA

GAEFLA-----SVC---NMKTVYVSNPTWG---NHKLVFKKAGFTTVADYTFWDYDNKRV

HIEKFLSDLESAPEKSVIILHGCAHNPTGMDPTQEQWKLVAEVIKRKNLFTFFDIAYQGF

ASGDPAADAWAIR---YFVDQG-MEMVVSQSFAKNFGLYNERVGNLTVVVNNPAVIA---

-GFQSQMSLVIRANWSNPPAHGARIVHKVLTTPARREQWNQSIQAMSSRIKQMRAALLRH

LM-DLGTP-GTWDHIIQQIGMFSYTGLTSAQVDHLIANHKVFLLRDGRINICGLNTKNVE

YVAKAIDETVRAVKSNI--------

>Gibberella_zeae_PH-1

------------------------------------------------------------

---MVSTTNFPAEVVPQAPEDPLFGLAREYKADDSPNKIDLGIGAYRDENAKPWVLPVVK

KADEILRNDPELNHEYAPIAGIASFTSKAAELVFGPDSSAIQDKRSTTLQTISGTGAVHL

GALFLAKFYK-------GNQTVYLSNPTWA---NHHQIFKNVGMS-IDTYPYFHKETKGL

DFEGFKKTLQSAPEGSVFVLHACAHNPTGVDPTQDQWTEIASIMKEKNHFPFFDTAYQGF

ASGDLVKDAWAIR---YFVDQG-FELVVAQSFAKNFGLYGERAGCFHAVTSPAPEASNTI

TRIGSQLAILQRSEISNPPLYGARIVSTVLNDRDLFAEWEENLRTMSGRIISMRDVLRAK

LE-ELETP-GTWNHITDQIGMFSFTGLSEKQVLQLREEFHVYMTKNGRISMAGLNENNVE

YFAKAVDKVVRDAA-----------

>Neurospora_crassa_OR74A

MAPASTLLRAAAPAPHAHTSFPSHHTPTSSPNRTTITNTNPSANSTSSTPLHRIQTIAKH

MA-LPQITSFPAEVVPQAPEDPLFGLARAFKADPSPQKVDLGIGAYRDENAKPWVLPVVK

KADEIIRNDPEANHEYLPIAGLASLTSKAAELVVGASAPAITEGRVASIQTISGTGACHL

GGLFLSRFYNPYGDA-SKKPTVYLSNPTWA---NHNQIFSNVGLP-IAQYPYFDQKTKGL

DIDGMKKALSDAPERSIILLHACAHNPTGVDPTLAQWREIAEIMAAKGHFPFFDTAYQGF

ASGDLDRDASAIR---LFVELG-FELVVAQSFAKNFGLYGQRAGCFHFISAPSPDAASIT

TRVASQLTLLQRSEISNPPIYGAKVASIVLNDPALFAEWKENLRTMSGRIIDMRKALRAK

LE-ELGTP-GPWNHITDQIGMFSFTGLNEKQVAKLREEFHIYMTKNGRISMAGLNKKNVD

YVARAVDKVVREVQ-----------

>Pyrenophora_tritici-repentis

--------------------MPDSAIPKLSSSSISSASSSPSQQTCSQS---RLTQISSH

IE-PMAATAFDANVVPQAPEDPLFGLMAAFRRDEDPKKVDLGIGAYRDDNAKPWVLPVVK

MADDRLRNDPNLNHEYLPIAGLPEFTTASQKLVLGADSPAIKEKRVTSLQTISGTGAVHL

GALFLAKFYKTQ-----SERTAYFSDPTWA---NHFQIFSNVGLQ-HKTYPYFSKKTKGL

DFDGMIGALESATEGSIIVLHACAHNPTGVDATQEQWKKIASVIKSKKHFPFFDTAYQGF

ASGDLATDAWSIR---YFVEQG-FEMCIAQSYAKNFGLYGERAGCFHFVTSPSSDAESTV

KRIASQLAILQRSEISNPPAYGARIASTVLNDPKLFAEWEENLRTMSGRIKEMRKALRSK

LE-DMGTP-GTWNHITEQIGMFSFTGLTEQQVLKIREDSHVYMTKNGRISMAGLNTHNID

YFAKAVDKVVRETQ-----------

>Aspergillus_niger

-----------MSPLSSSSSPSSSLSLTSSSPSSPSAASSSIASTSTSTARSRLASLSSH

IMGSTTPSVFSTAVVPAAPEDALFGLAQAFRQDSSDKKVDLVIGAYRDDNAKPWILPVVK

KADELVRNDPALNHEYLPIKGLADYTTAAQKLIIGADSPAIRENRVCTFQTISGTGAVHL

GALFLSKFHPSN-----PKPTVYLSNPTWA---NHNQIFTNVNLS-LANYPYFDPQTKGL

NFSGMLSALRDAPTGSIILLHVCAHNPTGVDLTQSQWKDVAVVMRERNHFPFFDCAYQGF

ASGDLIRDSWAVR---YFVEQD-FELCVAQSFAKNFGLYGQRTGAFHFVSAPGAEASQAN

AHVASQLAILQRSEISNPPAYGARIASRVLNDEGLFKEWEEDLKTMSGRIAEMRKGLRER

LE-KKGTP-GTWNHITDQIGMFSFTGLTESQVRVLKEKWHVYMTKNGRISMAGLNTHNLD

YFAEAVDSVVRETS-----------

>Ustilago_maydis_521

--------------------------------------------------------MGSI

SEQSSALAVWSA--VPQAPEDAIFKLTSSYKSDTYDKKVNLGVGAYRDNNGKPYVLPSVK

KAQADLIADESVDHEYLSITGLAEFTSAAAKLILGDDSPAIAEKRVASVQTISGTGANHL

GAVFLQRFYQ-YQAYGVDR-QIYISNPTWA---NHKAIFNTVGIK-PIDYPYYDAKTIAL

DFDGFTNTLRQAKNQSVFLLHACAHNPTGVDPTQEQWKQIADIFVEKGHFAFFDCAYQGF

ASGDLDRDAWAVR---HFVSRKSIPLLICQSFAKNAGLYGERVGALHVVSATPAQSA---

-AVFSQLAAIQRSEISNPPAFGARVVKMILTDPALFQQWQADVKEMAGRIITMRQSLFDL

LTKKFQTP-GNWDHILKQIGMFTFLGLDTNQCNRLLEEGHIYLTANSRISMAGLTTNNVE

YVASWIDKVVREKK-----------

>Puccinia_graminis

------------------------------------------------------------

--MPSTQNIFAS--LPLAPPDAIFQLTAAYKADQFQQKVNLGVGAYRDDSGKPWVLPVVK

TIKKELAEDENLDHEYQPITGLPSFTSASSTLILGSNSPAISENRVAKAQTISGTGANHL

GGLFLAKFYQPWQALPADQRVVYLSNPTWA---NHKAIFANMKLT-TKDYPYYDPKTIGL

DYQGFVDALNSAPPMSVFLLHACAHNPTGVDPTRDQWQELANIFLSKGHYAFFDCAYQGF

ASGDLDNDAWAVR---HFVDRK-IPLLICQSFAKNAGLYGERIGCLTVVAKDQDEAS---

-RIESQISVLQRSEISNPPAYGARIVSKILNEPQHFEQWKKDVREMADRIIDMRKQLKDL

LENKYKTP-GSWEHITRQIGMFSFTGLTPPQVEKMVENAHIYMTGNGRISMAGLNSSNVE

YVADCIDKAVRAKL-----------

>Saccharomyces_cerevisiae_S288c

------------------------------------------------------------

----MSATLFNN--IELLPPDALFGIKQRYGQDQRATKVDLGIGAYRDDNGKPWVLPSVK

AAEKLIHNDSSYNHEYLGITGLPSLTSNAAKIIFGTQSDAFQEDRVISVQSLSGTGALHI

SAKFFSKFFPDKL--------VYLSKPTWA---NHMAIFENQGLK-TATYPYWANETKSL

DLNGFLNAIQKAPEGSIFVLHSCAHNPTGLDPTSEQWVQIVDAIASKNHIALFDTAYQGF

ATGDLDKDAYAVRLGVEKLSTV-SPVFVCQSFAKNAGMYGERVGCFHLALTKQAQNKTIK

PAVTSQLAKIIRSEVSNPPAYGAKIVAKLLETPELTEQWHKDMVTMSSRITKMRHALRDH

LV-KLGTP-GNWDHIVNQCGMFSFTGLTPQMVKRLEETHAVYLVASGRASIAGLNQGNVE

YVAKAIDEVVRFYTIEAKL------

>Schizosaccharomyces_pombe

------------------------------------------------------------

----MSDYGFAN--IEEAKADAIFKLNAQYHQDEDPKKVNMSVGAYRDDTGKPWILPAVK

KASKIVEEQASFNHEYLPIAGLPRFTKAAAEVLFRPNPHLLSEDRVASMQSVSGTGANFL

AASFIETFYVKHTGA-----HVYISNPTWP---VHRTLWEKLGVT-VDTYPYWDAKNRSF

DYEGMLSTIKSAPEGSIFLLHACAHNPTGIDPTREQWLSIFESLLSRKHLVVFDIAYQGF

ASGDLNRDSWALN---EFVKYN-KDFFVCQSFAKNMGLYGERTGCMHY----VAKDASTK

NKVLSQLCIVQRNTISNPPAYGARIAAEILNSPQLFAEWEQDLKTMSSRIIEMRKRLRDS

LV-ALKTP-GSWDHITQQIGMFSFTGLTPAQVQFCQERYHLYFSANGRISMAGLNNSNVE

HVAQAFNHAVRELP-----------

>Populus_trichocarpa

-------------------------------------MSSSS------------------

------SSWWQS--VEPAPKDPILGVTEAFLADPSPDKVNVGVGAYRDDNGKPVVLQCVR

EAER-RIAG-NLNMEYLPMGGSVNMVEETLKLAYGENSEFIKDKRIAAVQSLSGTGACRL

FADFQKRFRP--------DSQIYIPVPTWA---NHHNIWRDAQVP-QRTYHYYHPESKGL

DFSALMDDIKNAPNGSFFLLHACAHNPTGVDPSEEQWREISHQFKVKGHFAFFDMAYQGF

ASGDPDRDAKSIR---IFLQDG-HHIGISQSYAKNMGLYGQRVGCLSVLCEDEKQAV---

-AVKSQLQQLARPMYSNPPVHGALVVSTILGDPELKKLWLKEVKVMADRIIGMRSALREN

LE-KLGSP-LSWKHITDQIGMFCYSGMTPEQVDRLTKEFHIYMTRNGRISMAGITTGNVG

YLANAINEVTSSA------------

>Oryza_sativa

-----------------------------------MAMSSSRSAAAIRGRRVGALARARR

HATRAMASLFGH--VEPASKDPILGVTEAFLADPSPDKVNVGVGAYRDDNGKPVVLECVR

EAER-RIAG-NLNMEYLPMGGSIKMIEESLKLAYGEDSDFIKDKRIAAVQALSGTGACRL

FADFQRRFLP--------NSQIYIPTPTWS---NHHNIWRDAQVP-QRTFTYYHPESRGL

DFAGLMDDIKNAPNGSFFLLHACAHNPTGVDPTEEQWREISYQFKIKNHFPFFDMAYQGF

ASGDPERDAKAIR---IFLEDG-HQIGCAQSYAKNMGLYGQRAGCLSILCEDEMQAV---

-SVKSQLQQIARPMYSNPPVHGALVVSIILNDPELKSLWLKEVKGMADRIIGMRKALREN

LE-GLGSP-LSWDHITNQIGMFCYSGMTPEQVDRLTNEYHIYMTRNGRISMAGVTTGNVA

YLANAIHEVTKTK------------

>Sclerotinia_sclerotiorum_1980_

------------------------------------------------------------

-----------M--YHKVLQYAILGITEAFKADSFKEKINLGVGAYRDDAGQPYVLPSVR

TAED-KVVKANLNKEYAGITGVPDFTKAAAVLAYGEGSSALD--RLVITQSISGTGALRI

GGAFLQRFYPG-------AKKIYIPNPSWA---NHAAVFKDSGLE-VEKYRYYNKDTIGL

DFEGMIADIKAAPKGSAFLLHACAHNPTGVDPTVEQWKEISEVVKASGHYAFFDMAYQGF

ASGDTDKDAFPIR---HFIKEG-HNPCLAQSFAKNMGLYGERVGAFSVVCADADEKK---

-RVDSQIKILVRPLYSNPPVHGARIASTILNDKALNKQWLGEVKGMADRIITMRALLKKE

LE-SLGSK-HDWSHITSQIGMFAYTGLTPEQMDKLAKEHSVYATKDGRISVAGITTANVK

RLAAAIHAVKP--------------

>Physcomitrella_patens

------------------------------------------------------------

----MAPSVFEH--VQQAPEDPILGVTVAYNKDPSPLKVNLGVGAYRTEEGKPLVLNVVR

RAEQQLVADRSRNKEYQPITGISQFNKLSAKLILGANSPAIAENRVATVQALSGTGALRV

GAEFISRHYA--------KPIIFLPNPTWG---NHNKIFPLGGVP-QKPYRYYDPKTRGL

DYEGMLEDLKAAPDGAVILLHACAHNPTGVDPTEEQWEGIRQVIRSKHQLPFFDCAYQGF

ASGSLDKDAHAVR---LFVADG-GECFVAQSYAKNMGLYGERVGALSVVCTNAAVAS---

-RVDSQLKLVIRPMYSSPPAHGAAIAATILADGRLFQEWTVELKGMADRIISMRQQLYDA

LQ-ARGTP-GDWTHVLKQIGMFTFTGLNKSQVEFMTRQYHIYMTSDGRISMAGLSSKTVP

HLADAIHAAVVGAQRKS--------

>Selaginella_moellendorffii

----------------------------------------------------------ME

ATSAAAASVFDH--LEQAPEDPILGITVAYNKDPHPGKVNLGVGAYRTEEGKPLILNVVR

RAEERLLADRSKNKEYLPITGLADFNKRSAMLILGSDSPAIVEKRLVTAQCLSGTGSLRV

GAEFLARHYG--------VKLVFLPTPTWG---NHFKVFMNAGLA-VKTYRYYDNKTRGL

DYEGMLEDIGAAPSGSVILLHACAHNPTGVDPTPQQWEGIRQAIRNRGHLPFFDSAYQGF

ASGSLDKDAHAVR---TFVADG-GECFIAQSFAKNLGLYGERVGALSIVTKSSGVAT---

-RVESQLKLVIRPMYSNPPIHGASIVALVLGDGDLFSEWTVELKGMADRIISMRHKLYDA

LR-ARGTP-GDWTHILKQIGMFSFTGLNKEQVQFMTREYRIYMTLDGRISMAGLSLKTVP

LLADAIHAAVTGAKA----------

>Arabidopsis_thaliana

-----------------------------------------------------------M

D------SVFSN--VARAPEDPILGVTVAYNNDPSPVKINLGVGAYRTEEGKPLVLDVVR

KAEQQLVNDPSRVKEYIPIVGISDFNKLSAKLILGADSPAITESRVTTVQCLSGTGSLRV

GAEFLKTHYH--------QSVIYIPKPTWG---NHPKVFNLAGLS-VEYFRYYDPATRGL

DFKGLLEDLGAAPSGAIVLLHACAHNPTGVDPTSEQWEQIRQLMRSKSLLPFFDSAYQGF

ASGSLDTDAQSVR---TFVADG-GECLIAQSYAKNMGLYGERVGALSIVCKSADVAS---

-KVESQVKLVVRPMYSSPPIHGASIVATILKSSDMYNNWTIELKEMADRIKSMRQQLFEA

IQ-ARGTP-GDWSHIIKQIGMFTFTGLNKEQVEFMTKEFHIYMTSDGRISMAGLSSKTVP

HLADAMHAAVTRLG-----------

>Vitis_vinifera

-----------------------------------------------------------M

NSQHPDGSVFSN--IVRAPEDPILGVTVAYNKDTSPIKLNLGVGAYRTEEGKPLVLNVVR

RAEQLLVNDPSRVKEYLPIVGLAEFNKLSAKLIFGADSPAIQENRVATVQGLSGTGSLRI

GAEFLARHYY--------QHTIYIPVPTWG---NHPKIFTIAGLS-VKTYRYYDPETRGL

DFKGLLEDLGAAPTGAIVLLHACAHNPTGVDPTLEQWEQIRQLMRSKGLLPFFDSAYQGF

ASGSLDADAQSVR---MFVADG-GECLAAQSYAKNMGLYGERVGALSIVCKAADVAS---

-RVESQLKLVIRPMYSNPPIHGASIVATILKDSDMYNEWTLELKAMADRIISMRQLLFDT

LR-DRGTP-GDWSHIIKQIGMFTFTGLNTEQVAFMTKEYHIYMTSDGRISMAGLSSRTVP

HLADAIHAAVTRIP-----------

>Zea_mays

-----------MATAAAFSVSSPAASAVAARSKVFGGVNQARTRTGCRVGITRKNFGRVM

MALAVDVSRFEG--VPMAPPDPILGVSEAFKADKSELKLNLGVGAYRTEELQPYVLNVVK

KAENLMLE-KGENKEYLPIEGLAAFNKATAELLLGADNPVINQGLVATLQSLSGTGSLRL

AAAFIQRYFP--------EAKVLISSPTWG---NHKNIFNDARVP-WSEYRYYDPKTVGL

DFEGIIADIEAAPEGSFVLLHGCAHNPTGIDPTPEQWEKIADVIQEKKHMPFFDVAYQGF

ASGSLDEDAFSVR---LFVKRG-MEVFVAQSYSKNLGLYSERVGAINVVCSAPEVAD---

-RVNSQLKRLARPMYSNPPIHGAKIVANVVGDPTMFGEWKQEMELMAGRIKNVRQKLYDS

LS-AKDKSGKDWSFILRQIGMFSYTGLNKAQSDNMTDKWHIYMTKDGRISLAGLSLAKCD

YLADAIIDSFHNVN-----------

>Chlamydomonas_reinhardtii

-----------MQLQARMQLRS------------------AGPRRAGPVPVR--------

---AMAGSRFAD--VAQAPPDPILGVSEAFKASTDPNKLNLGVGAYRDEQLKPVVLSVVK

KAESKIFS-RNENKEYLTIEGLDAFRKATVDLLLGGDHPAIKEGRVAVVQSLSGTGSLRV

GAAFIARFMK--------GATVYLSNPTWG---NHRNIFGDEGVE-WKYYRYFDADTVGL

DFRGMVQDLQAAPPGSVVVLHGCAHNPTGIDPTKDQWAAIADLCKERNLMPFFDVAYQGF

ATGDLDKDAYAPR---LFVEKG-LEIVVSQSYSKNLGLYGERVGALVMVLNDKEAAT---

-RCLSQLKRLARALYSNPPTHGARIAAEVVNDKELFEEWKGEMRGMAGRIERVRGELQRS

LE-SKYPS-KDWSFITKQIGMFSFTGLTPAQVDNMTNKHAIFMTRDGRISLAGLNSAKVD

YLAEAIVDSVRNH------------

**Supplementary Material 5**. Amino acid conservation between clades for three ReGens. Graphs are colored according to the clade comparison code defined in Figure 1B. Pairwise comparisons of proteins from each clade were plotted along their multiple alignment extension (*x*-axis) and higher values (*y*-axis) represent more similar regions in the interclade comparison. The *y*-axis represents the proportion of amino acid substitutions in a given position of the multiple alignment (*x*-axis); positions with gaps were removed.
(**A**) SD; (**B**) AATc; (**C**) CTH.


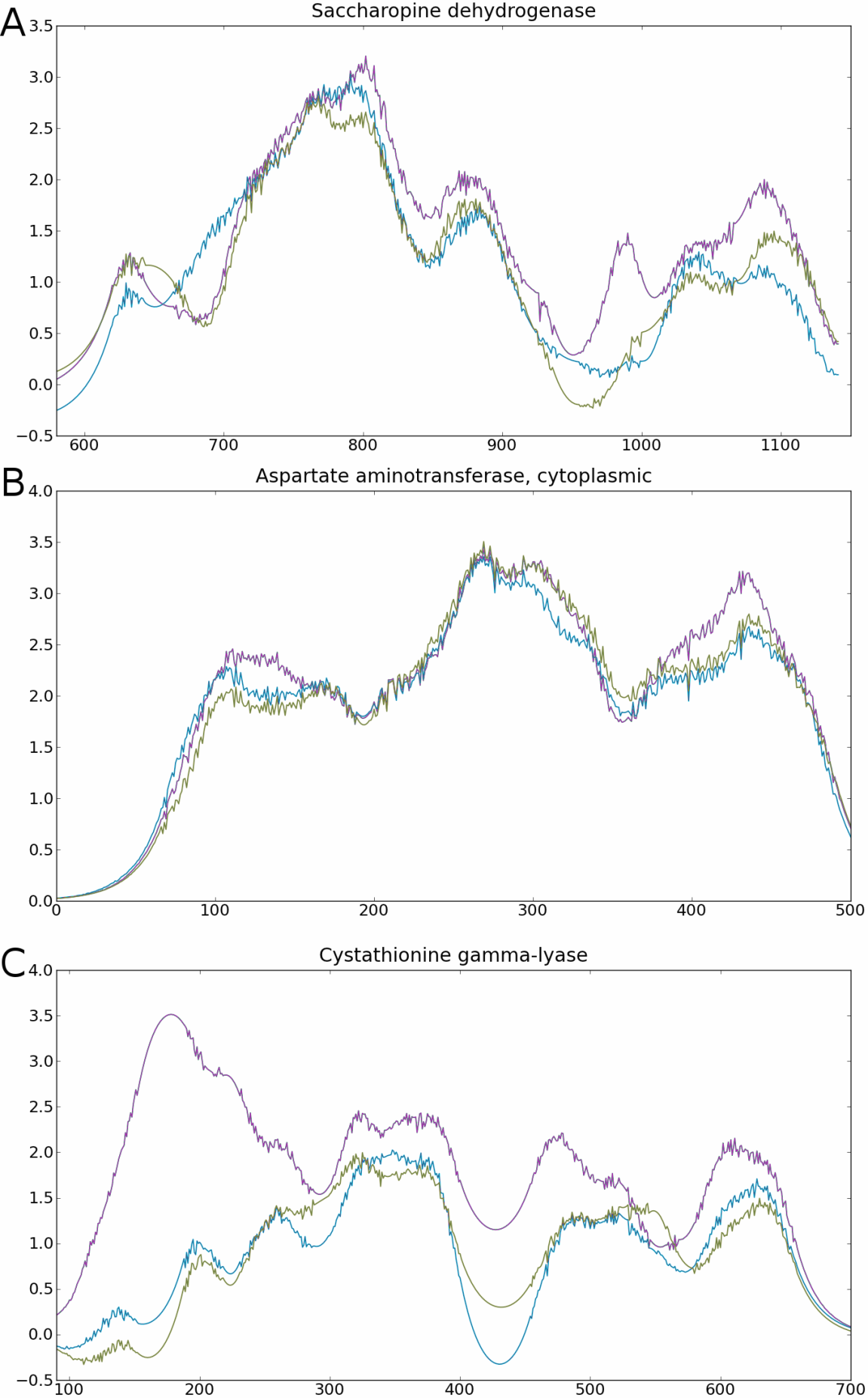


**Supplementary Material 6.** Conservation plots including standard deviation. Graphs are colored according to the clade comparison code defined in Figure 1B, dashed lines represent mean ± standard deviation. Standard deviation data were filtered for high-frequency noise using the low-pass filter. (**A**) ALS; (**B**) BHMT; (**C**) BCA; (**D**) SD; (**E**) CTH; (**F**) AATm; (**G**) AATc.

| (A) |
| --- |
| 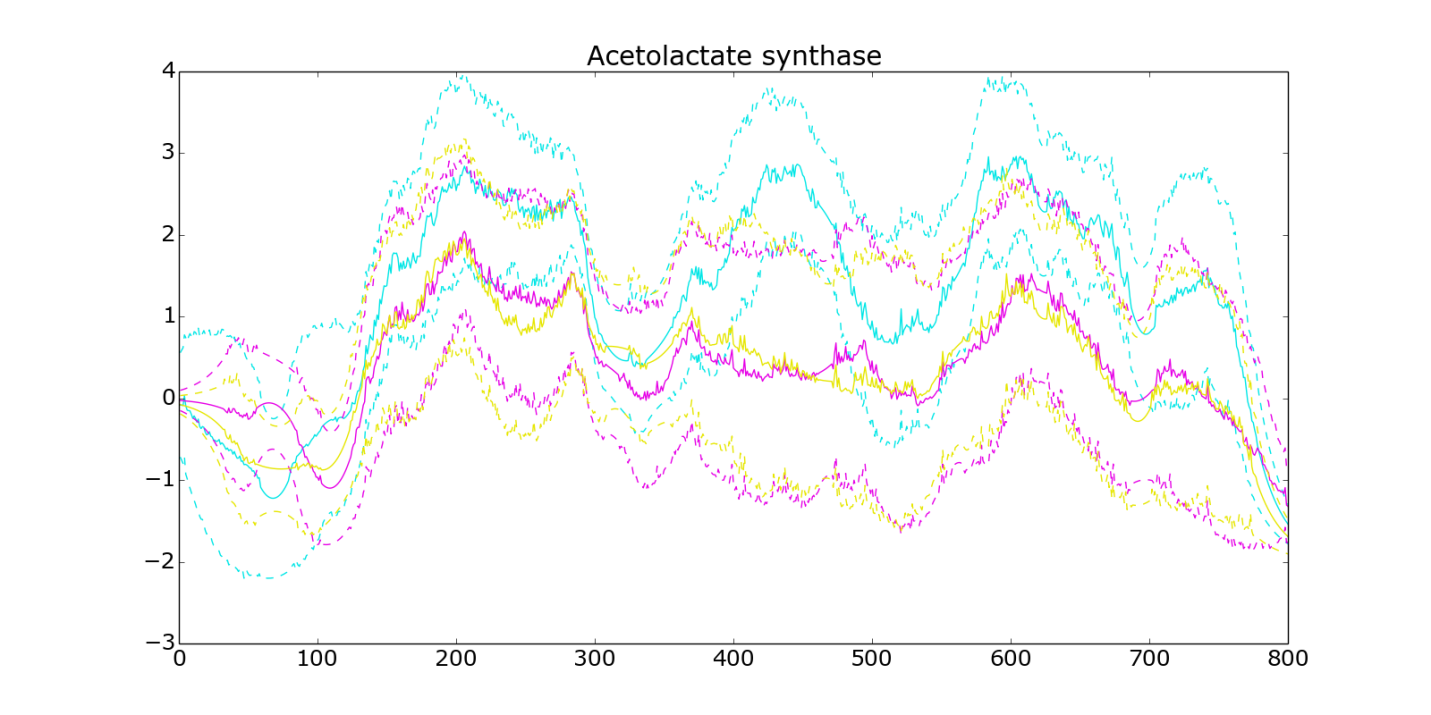 |
| (B) |
| 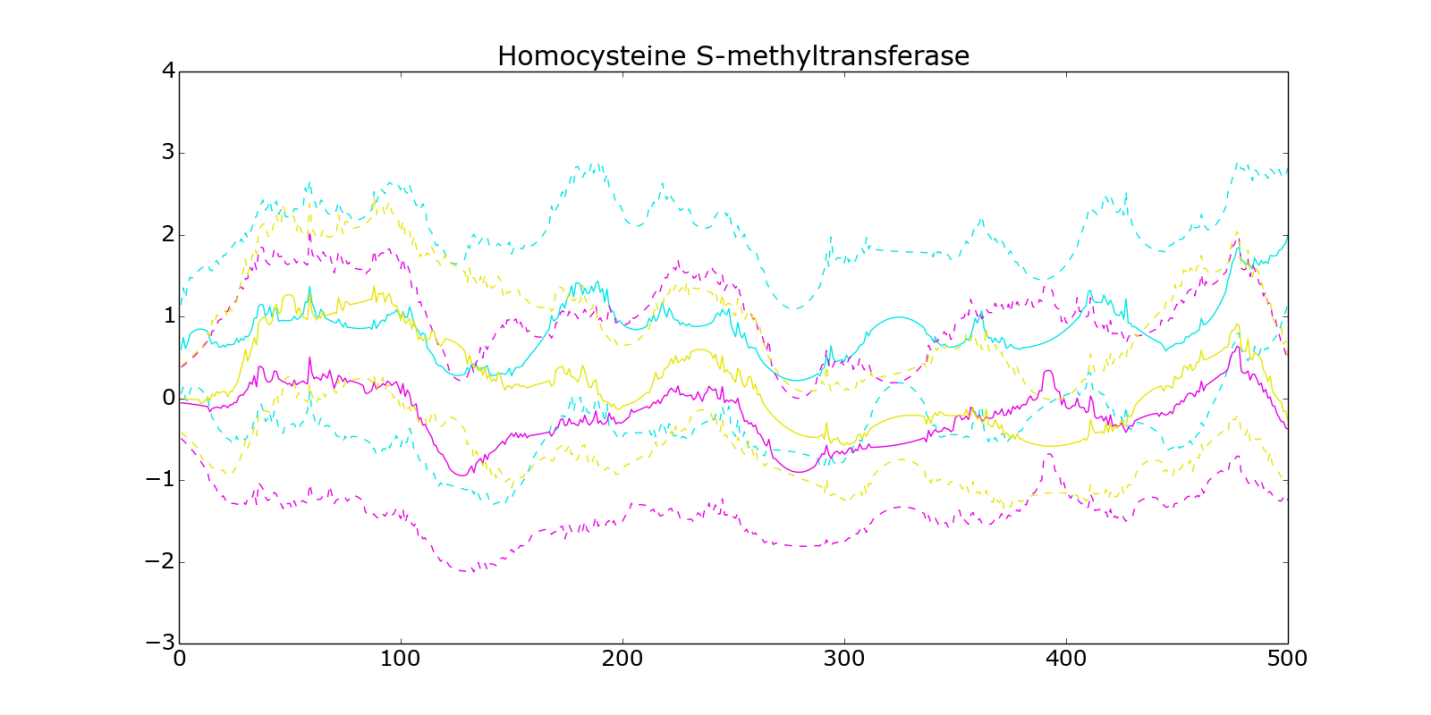 |
| (C) |
| 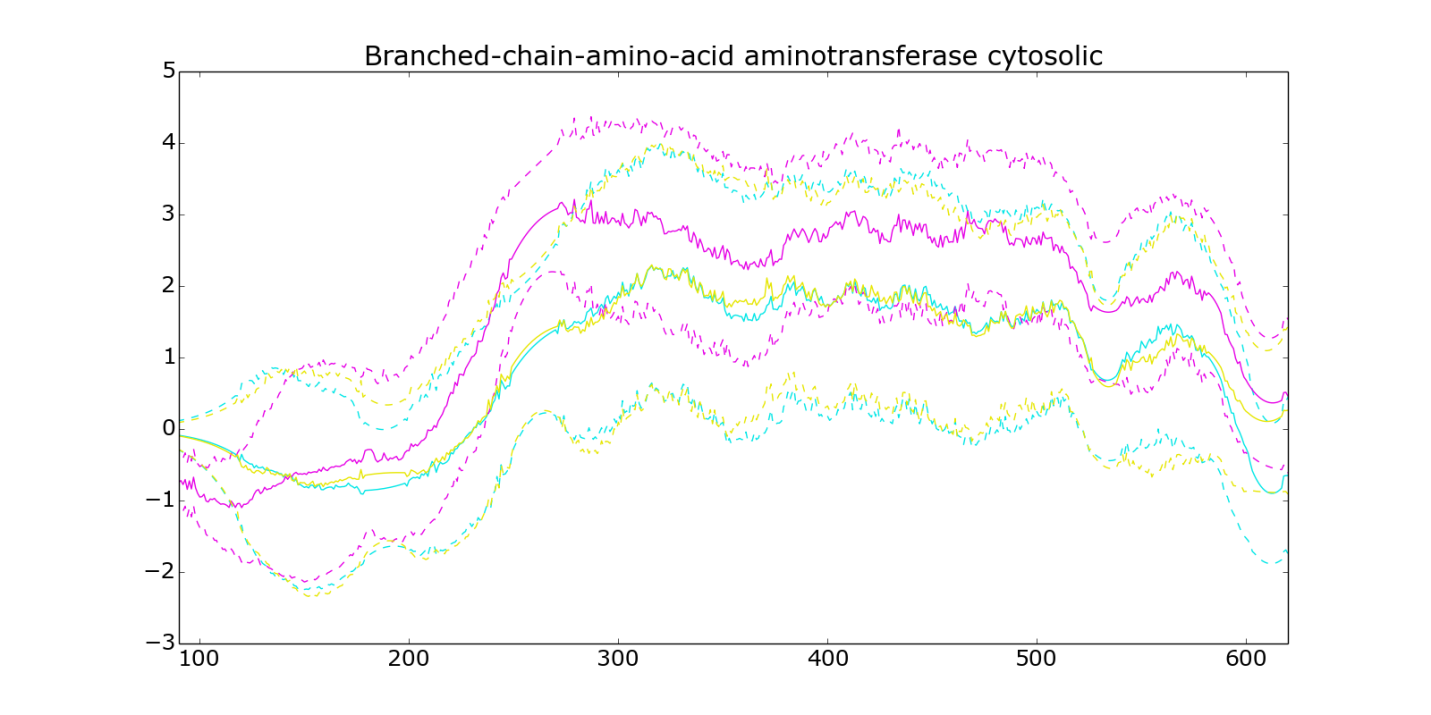 |

**Supplementary Material 6.** *Cont.*

| (D) |
| --- |
| 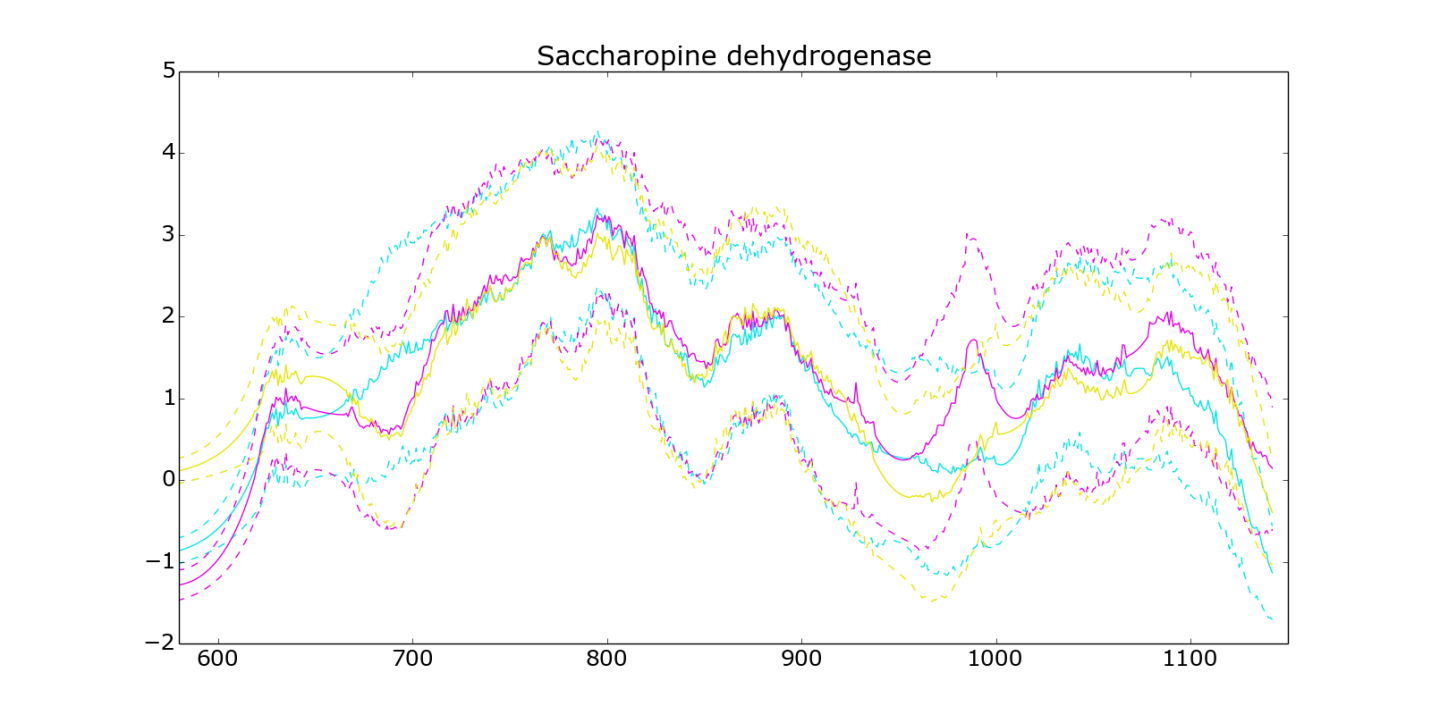 |
| (E) |
| 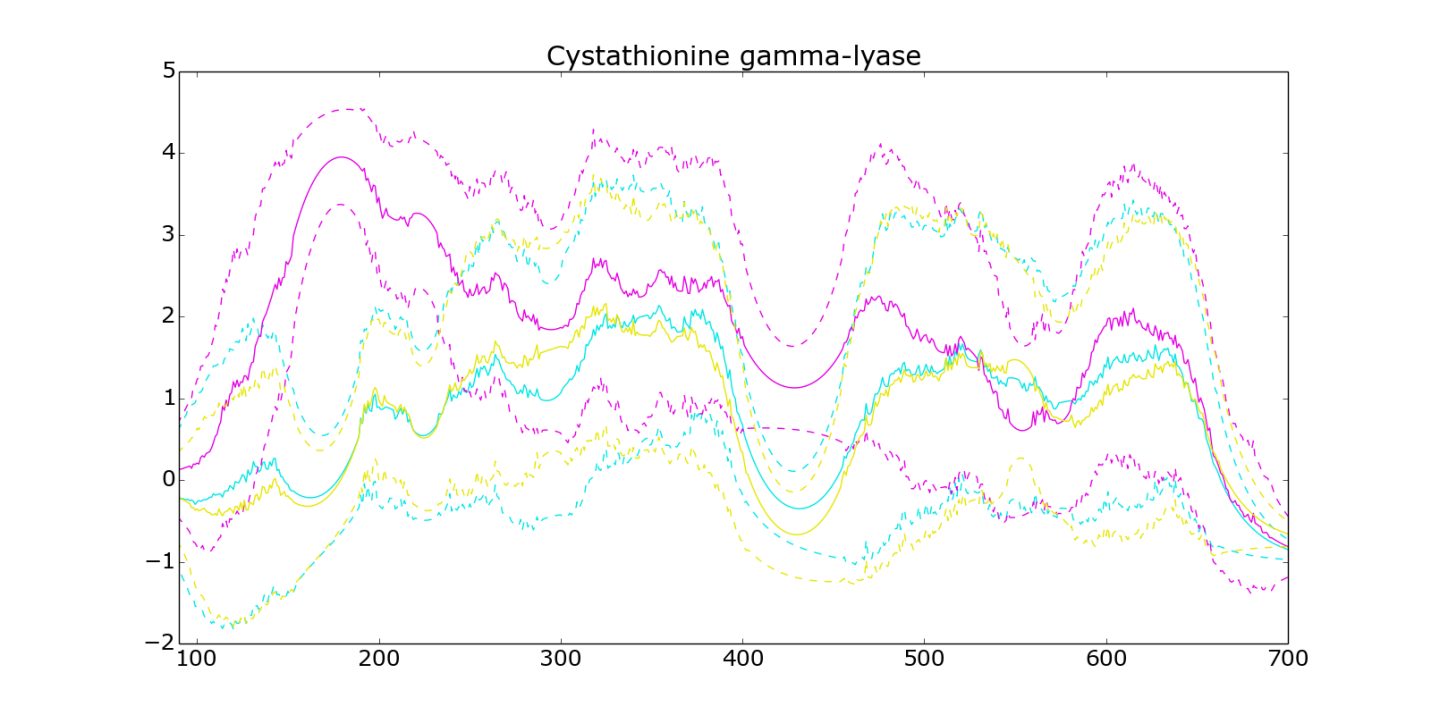 |
| (F) |
| 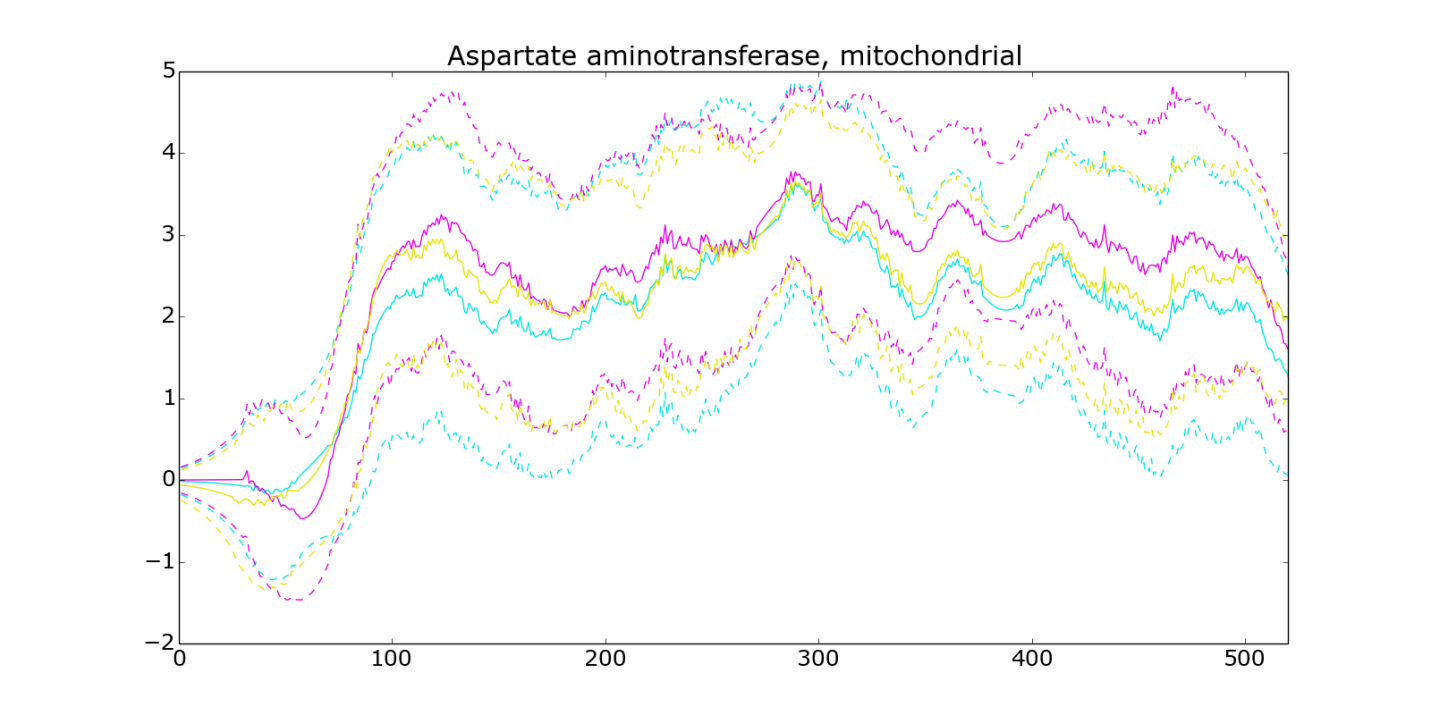 |

**Supplementary Material 6.** *Cont.*

| (G) |
| --- |
| 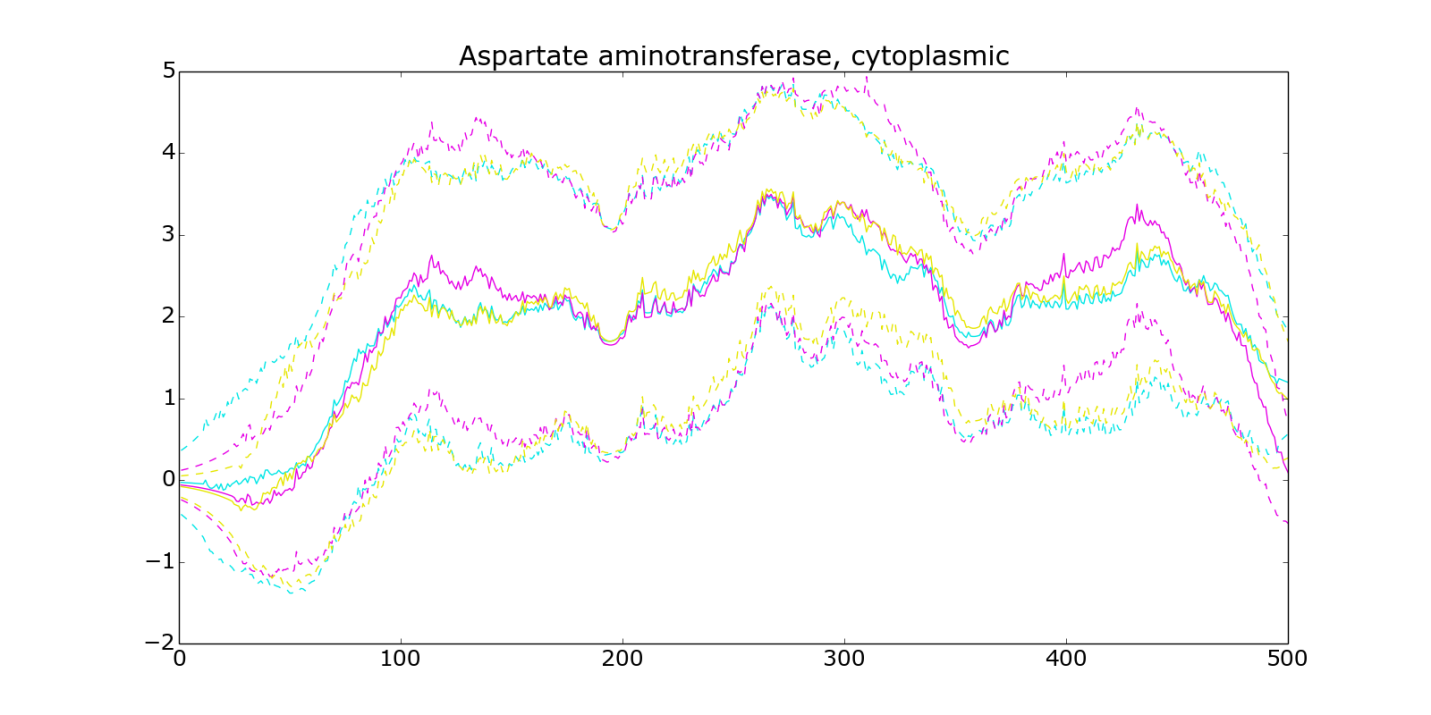 |

**Supplementary Material 7.** Pairwise comparison of highly divergent regions in ALS and BHMT proteins. Alignment of all sequences from the three clades and pairwise comparisons between fm, pf, and pm using the color code defined in Figure 1B. Amino acids that are more conserved between pairwise clades than in all-against-all comparisons are identified by “*”. Alignment gaps were removed in all-against-all comparison.


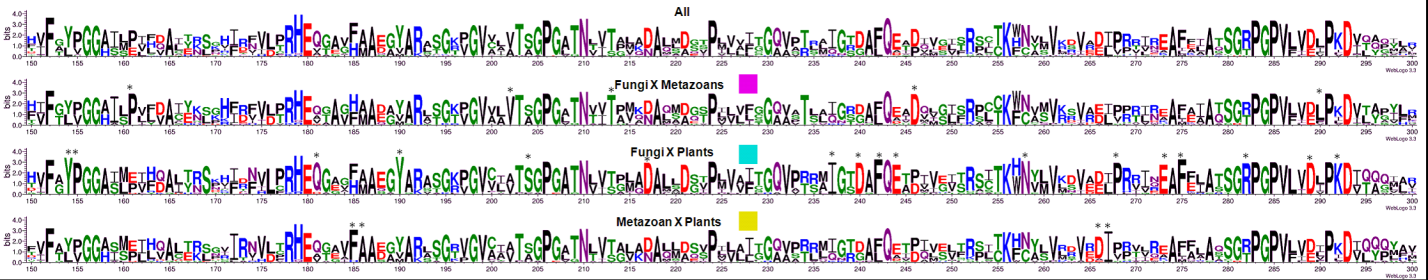


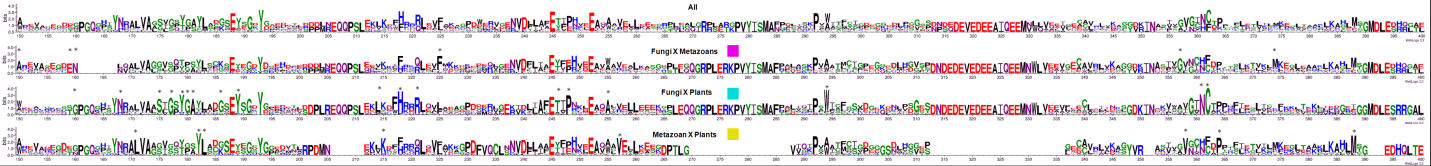


**Supplementary Material 8.** Original PhyML trees for all ReGens. ReGens protein phylogenies performed with PhyML inferred using the Maximum Likelihood method based on the JTT matrix with 500 bootstrap replicates.

| ALS |
| --- |
| 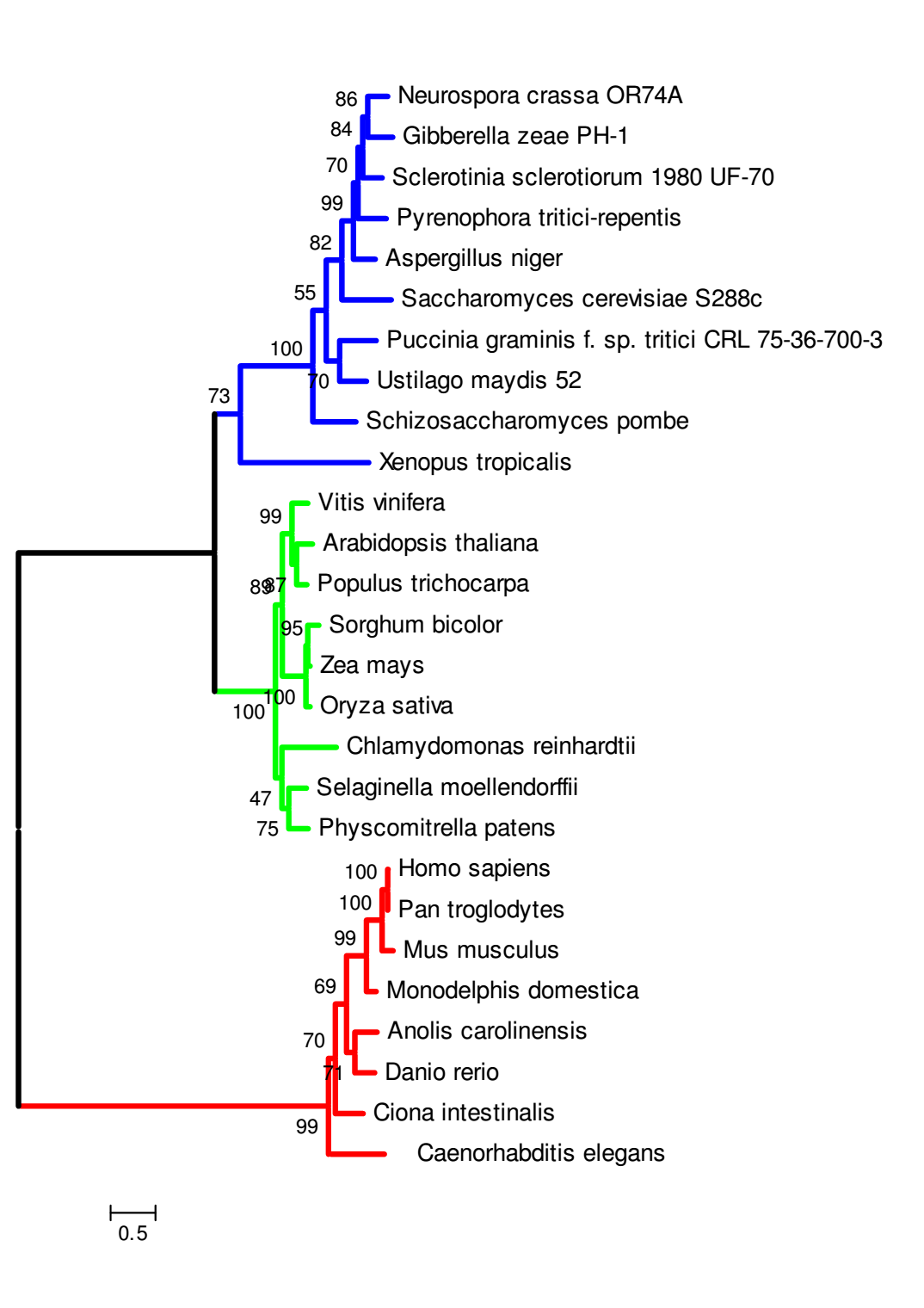 |

**Supplementary Material 8.** *Cont.*

| BHMT |
| --- |
| 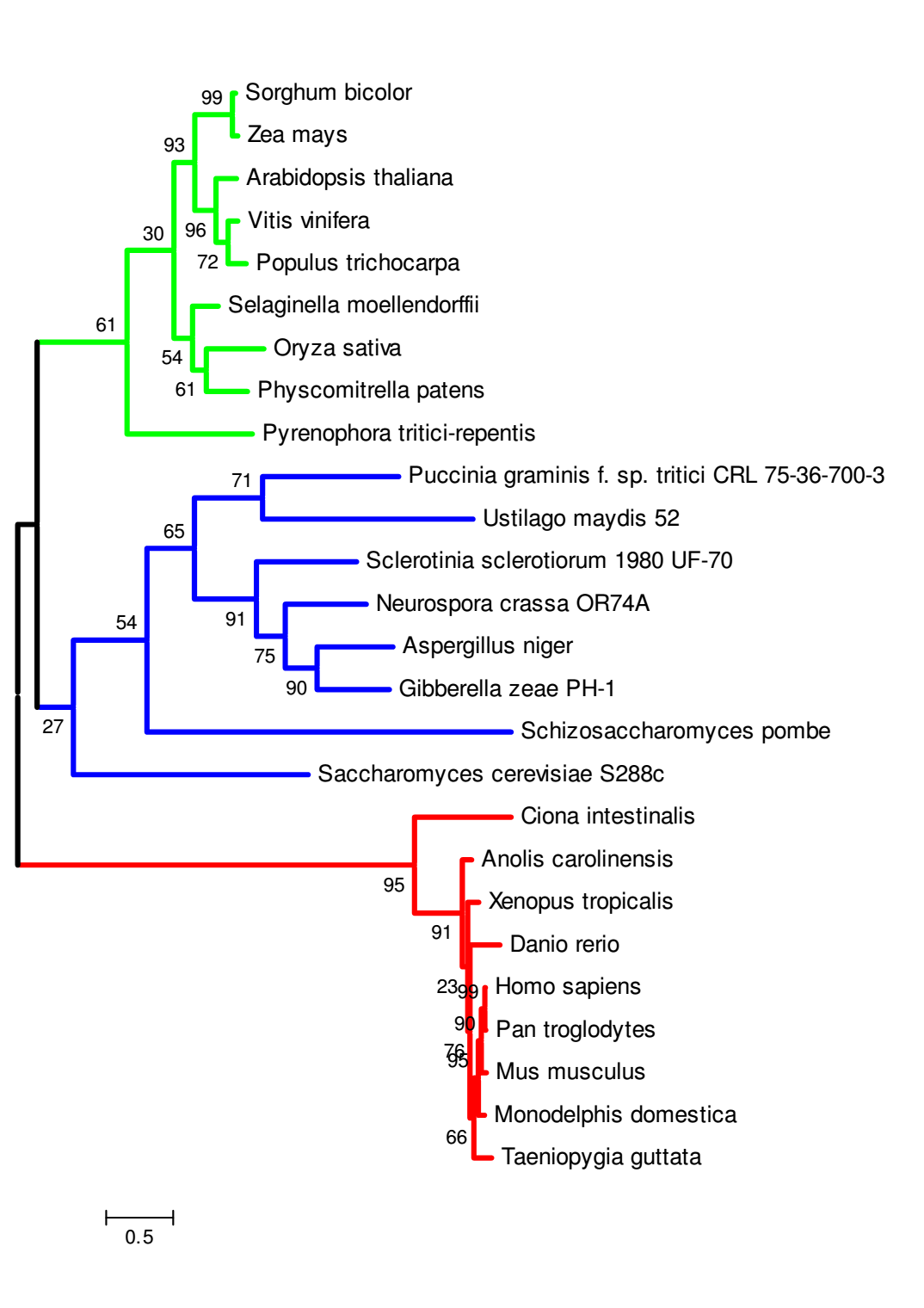 |

**Supplementary Material 8.** *Cont.*

| BCA |
| --- |
| 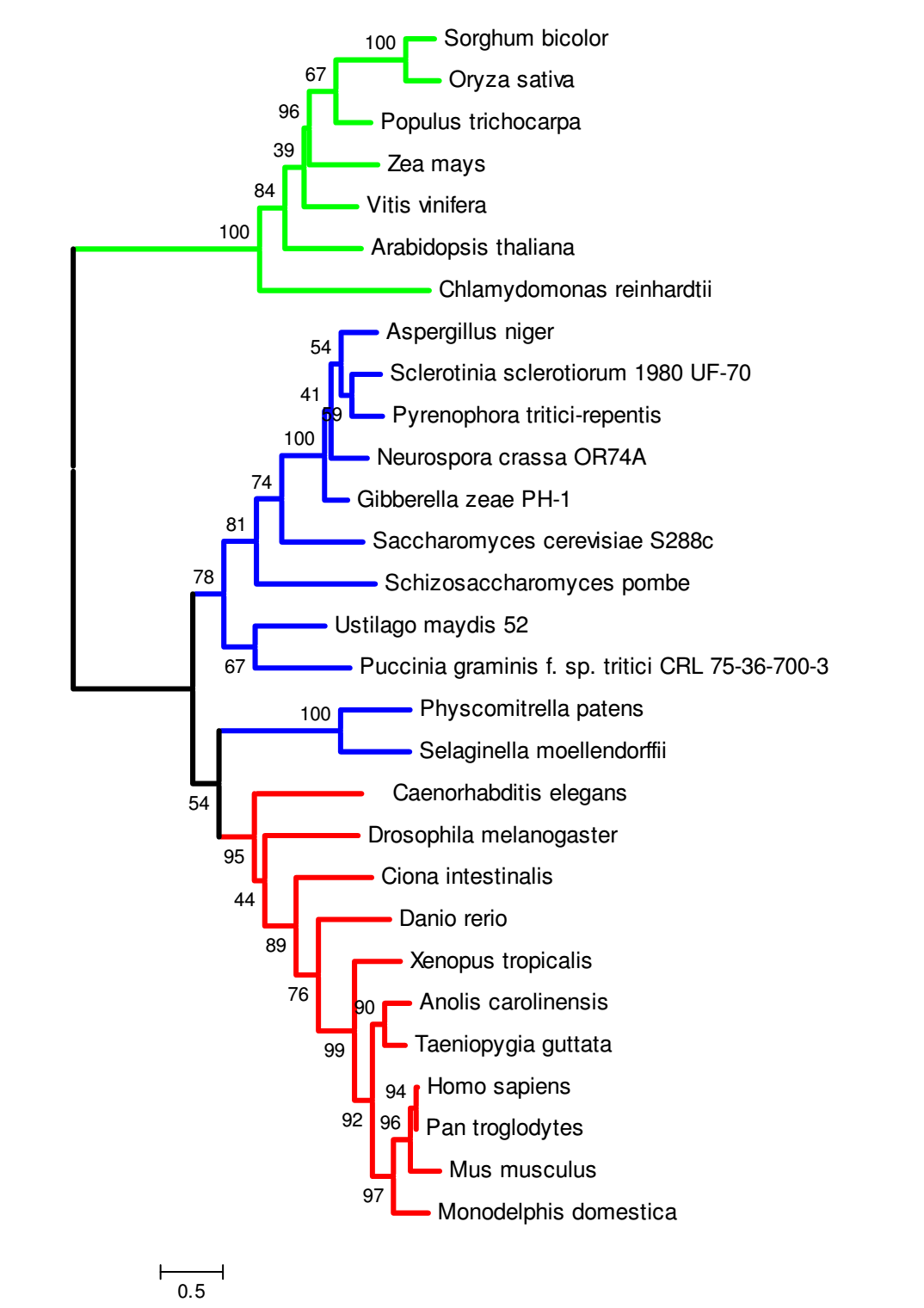 |

**Supplementary Material 8.** *Cont.*

| SD |
| --- |
| 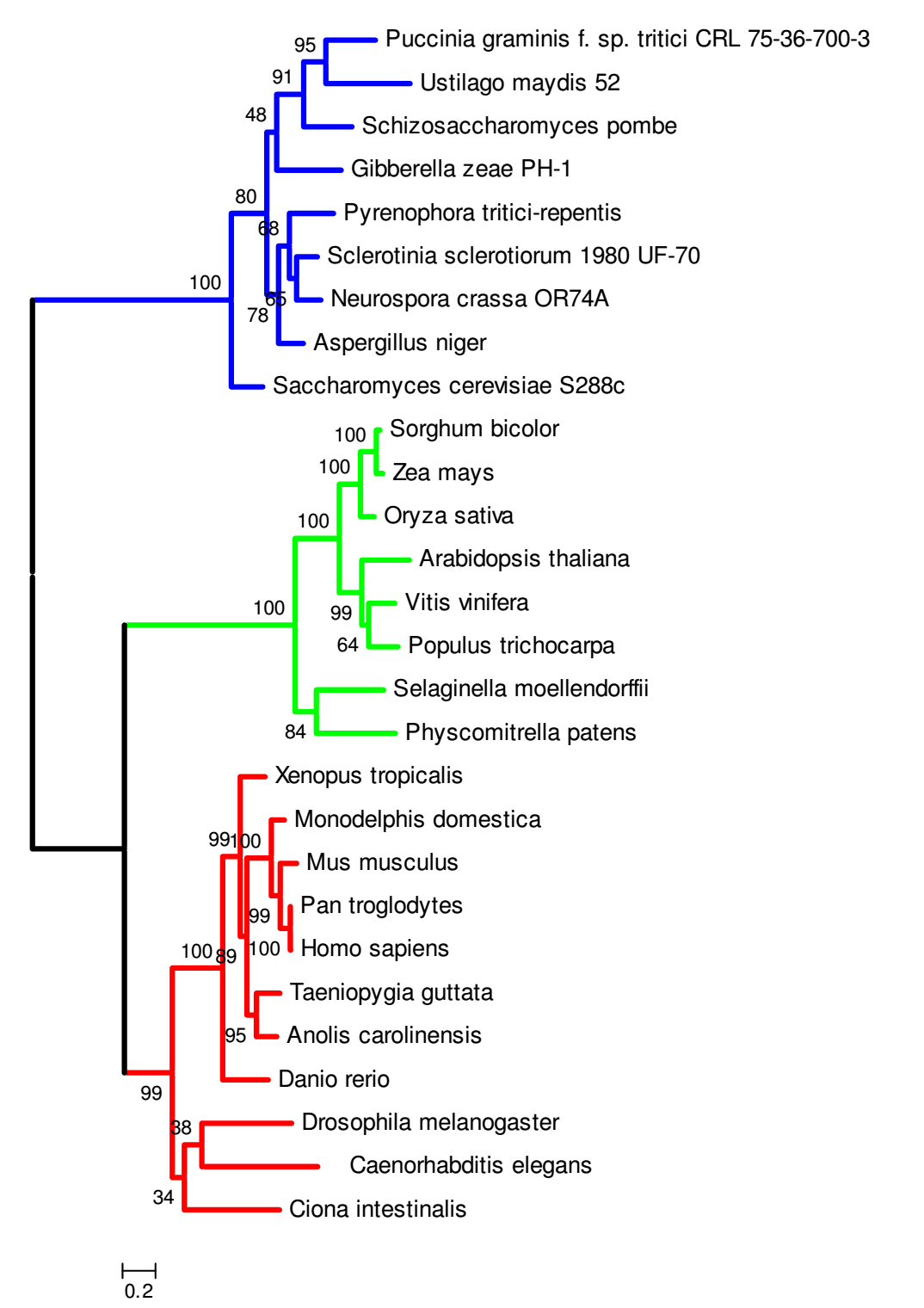 |

**Supplementary Material 8.** *Cont.*

| CTH |
| --- |
| 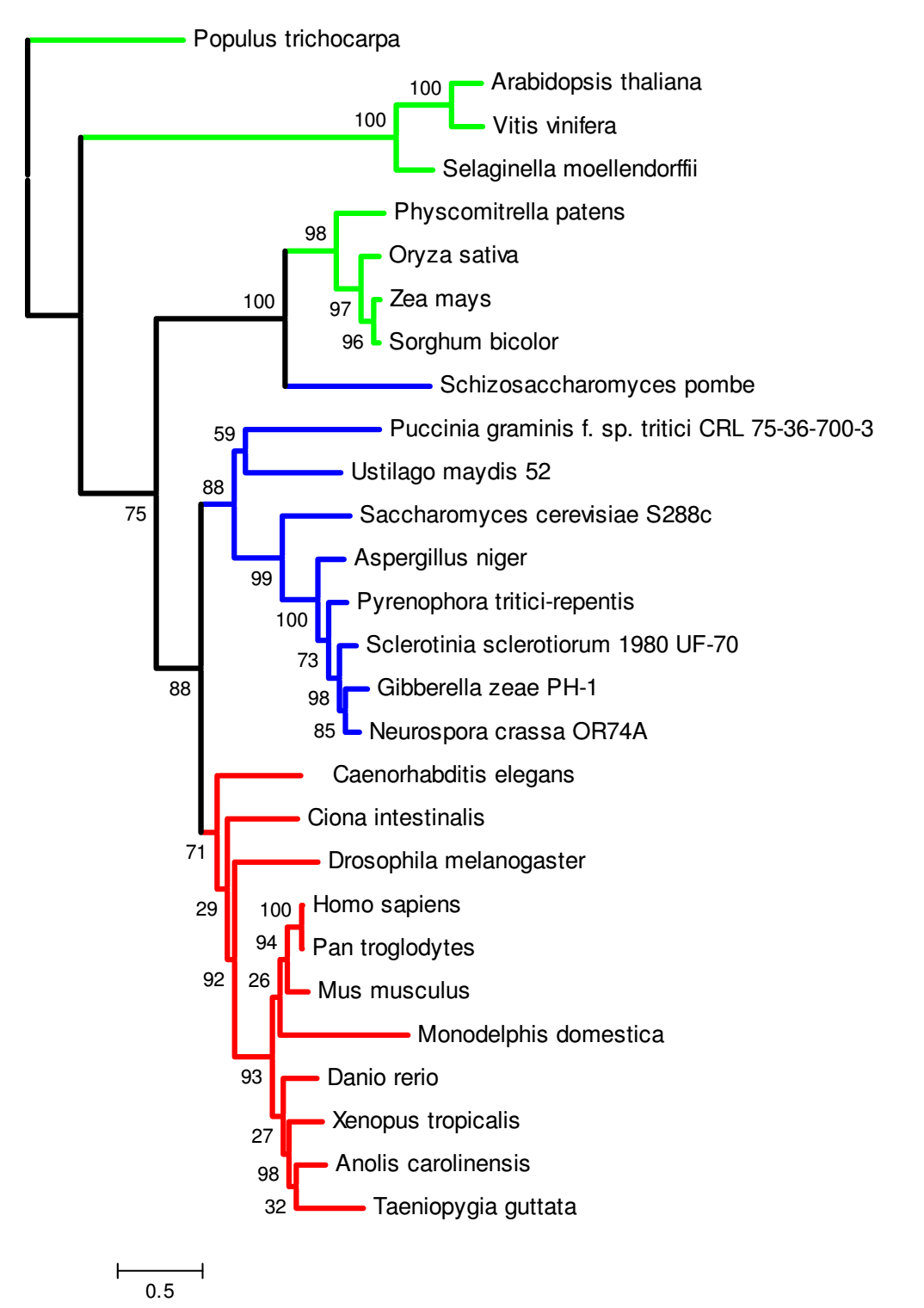 |

**Supplementary Material 8.** *Cont.*

| AATm |
| --- |
| 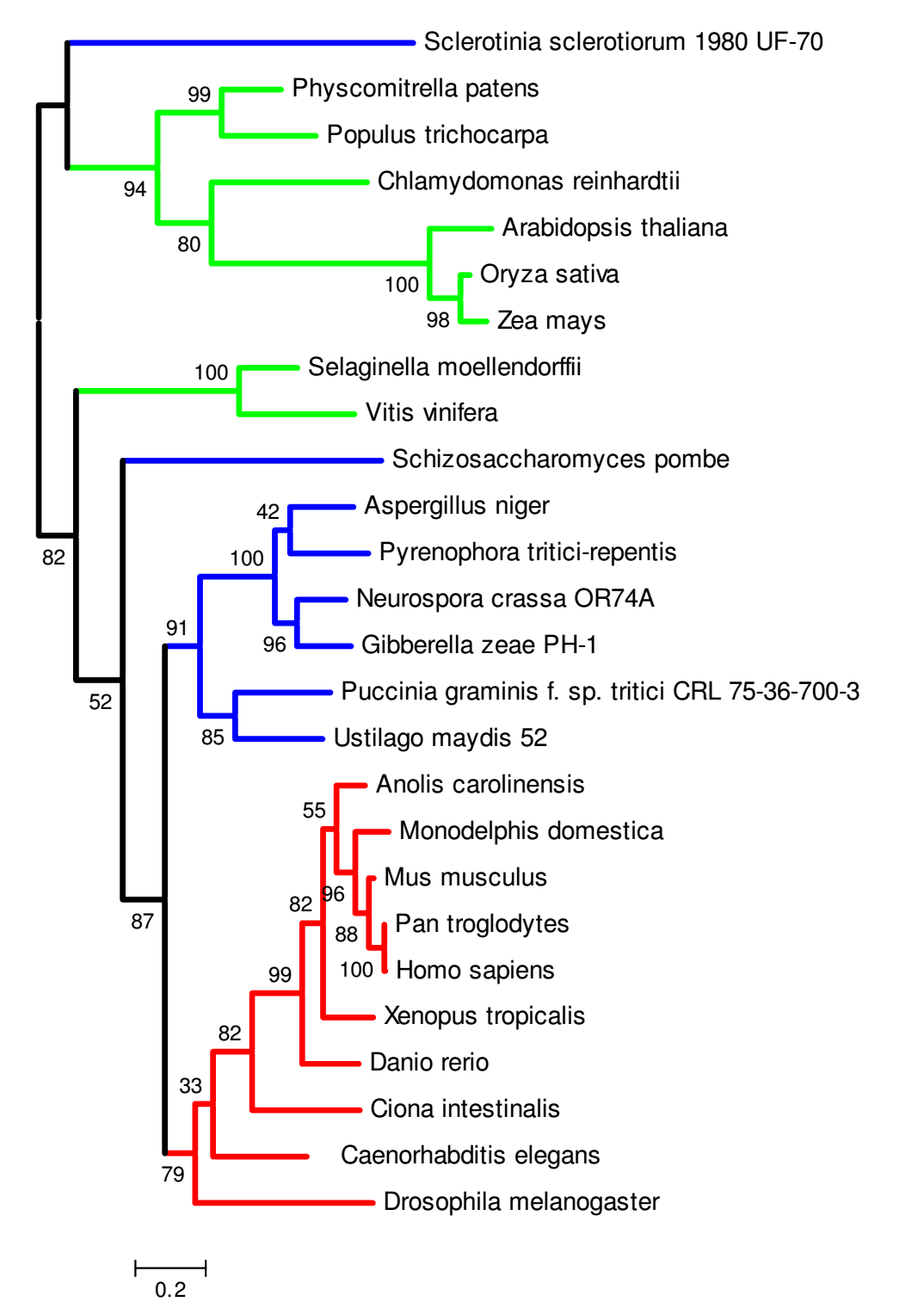 |

**Supplementary Material 8.** *Cont.*

| AATc |
| --- |
| 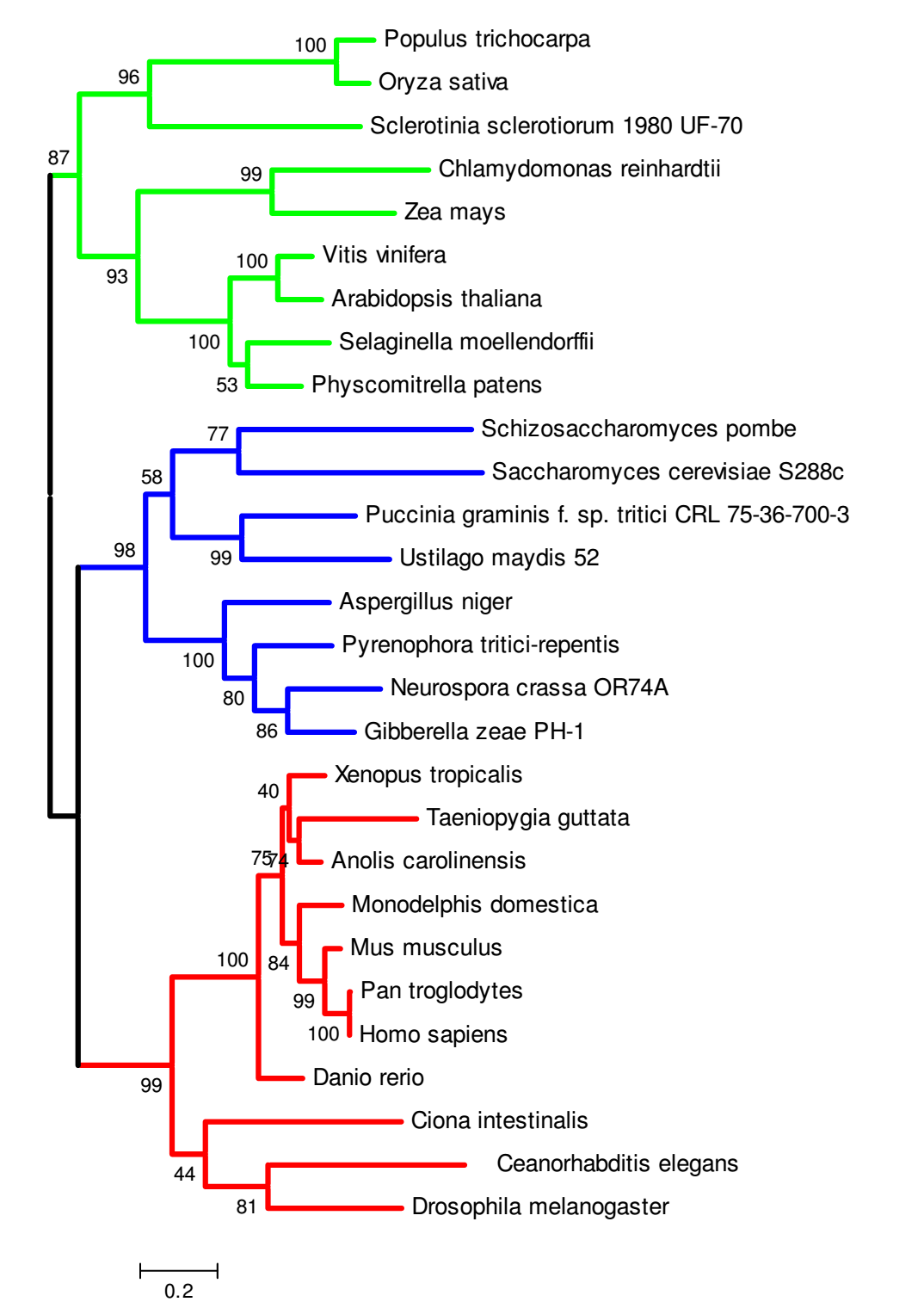 |

© 2014 by the authors; licensee MDPI, Basel, Switzerland. This article is an open access article distributed under the terms and conditions of the Creative Commons Attribution license (http://creativecommons.org/licenses/by/4.0/).
